# Phylogeny, Global Biogeography and Pleomorphism of *Zanclospora*

**DOI:** 10.3390/microorganisms9040706

**Published:** 2021-03-29

**Authors:** Martina Réblová, Miroslav Kolařík, Jana Nekvindová, Andrew N. Miller, Margarita Hernández-Restrepo

**Affiliations:** 1 Department of Taxonomy, Institute of Botany, The Czech Academy of Sciences, 252 43 Průhonice, Czech Republic; 2 Laboratory of Fungal Genetics and Metabolism, Institute of Microbiology, The Czech Academy of Sciences, 142 20 Prague, Czech Republic; mkolarik@biomed.cas.cz; 3 Department of Clinical Biochemistry and Diagnostics, University Hospital Hradec Králové, 500 05 Hradec Králové, Czech Republic; nekvindova@fnhk.cz; 4 Illinois Natural History Survey, University of Illinois Urbana-Champaign, Champaign, IL 61820, USA; amiller7@illinois.edu; 5 Westerdijk Fungal Biodiversity Institute, 3508 AD Utrecht, The Netherlands; m.hernandez@wi.knaw.nl

**Keywords:** Chaetosphaeriales, conidiogenesis, geographic distribution, GlobalFungi, life cycle, molecular systematics, taxonomic novelties, new typification

## Abstract

*Zanclospora* (*Chaetosphaeriaceae*) is a neglected, phialidic dematiaceous hyphomycete with striking phenotypic heterogeneity among its species. Little is known about its global biogeography due to its extreme scarcity and lack of records verified by molecular data. Phylogenetic analyses of six nuclear loci, supported by phenotypic data, revealed *Zanclospora* as highly polyphyletic, with species distributed among three distantly related lineages in Sordariomycetes. *Zanclospora* is a pleomorphic genus with multiple anamorphic stages, of which phaeostalagmus-like and stanjehughesia-like are newly discovered. The associated teleomorphs were previously classified in *Chaetosphaeria*. The generic concept is emended, and 17 species are accepted, 12 of which have been verified with DNA sequence data. *Zanclospora* thrives on decaying plant matter, but it also occurs in soil or as root endophytes. Its global diversity is inferred from metabarcoding data and published records based on field observations. Phylogenies of the environmental ITS1 and ITS2 sequences derived from soil, dead wood and root samples revealed seven and 15 phylotypes. The field records verified by DNA data indicate two main diversity centres in Australasia and Caribbean/Central America. In addition, environmental ITS data have shown that Southeast Asia represents a third hotspot of *Zanclospora* diversity. Our data confirm that *Zanclospora* is a rare genus.

## 1. Introduction

*Zanclospora* [1], typified with *Z. novae-zelandiae*, was established for dematiaceous hyphomycetes observed on plant litter or decaying wood and bark and characterised by setiform conidiophores, discrete phialides arranged in whorls and hyaline, unicellular, non-setulate conidia in slimy masses enveloping the conidiophores [2,3,4,5,6,7,8,9,10]. However, the morphological characters of conidiophores, phialides and conidia vary among species and contribute to the phenotypic heterogeneity of the genus. Conidiophores are simple or branched, occasionally accompanied by setae, branches are fertile, resembling the main stalk with secondary and tertiary branches often developed, or they are sterile and setiform, inserted into the main stalk. Conidiogenous cells are either tightly appressed to the conidiophore in multiple whorls forming a compact fertile zone or divergent in several loose whorls. Phialides possess indistinct or well-defined, flared to tubular collarettes. The conidial shape varies from falcate, obovoid to bacilliform. The teleomorph-anamorph connection has been established for only two species, both with a teleomorph attributed to *Chaetosphaeria*, namely the *Z. brevispora* anamorph of *Ch. brevispora* [11] and *Zanclospora* sp. anamorph of *Ch. lateriphiala* [12].

To date, ten species and two varieties were introduced in *Zanclospora* [1,2,3,4,5,6,7,8,9,10], but little is known about the systematic placement, relationships and global geographical distribution of these taxa. Moreover, the genus is under-represented in culture collections. Using molecular data, Fernández et al. [13] and Hernández-Restrepo et al. [9] confirmed the placement of *Z. iberica* and *Ch. lateriphiala* in the *Chaetosphaeriaceae*. *Zanclospora* is similar to *Cryptophiale* [14,15], *Cryptophialoidea* [16], and *Kionochaeta* [17] in having pigmented conidiophores with a setiform extension, lateral phialides usually arranged in fertile zones and hyaline conidia. Our observations indicate that conidial structures similar to two dematiaceous hyphomycete genera, *Stanjehughesia* [18] and *Phaeostalagmus* [19], can occur on a natural substrate and in culture.

Although there are only a few published records of *Zanclospora*, *Z. novae-zelandiae* seems to be an exception to the rule. In the protologue of *Z. novae-zelandiae* [1], conidia were reported with a relatively large range of lengths, 18–35 μm long. On the other hand, the specimens listed by these authors [1] shared the same morphological diagnostic characters such as colourless disk-like excrescences on the upper setiform part of conidiophores and branches, branched conidiophores and falcate conidia. However, the large range of conidial lengths provided an opportunity to expand the species concept further, and the published morphological profiles of *Z. novae-zelandiae* vary considerably. Several authors [8,20,21,22,23] reported *Z. novae-zelandiae* from different geographical regions with a variable conidial length and introduced other features. The conidiophore wall lacked ornamentation, and some collections contained uniformly unbranched conidiophores. These differences may indicate cryptic species within *Z. novae-zelandiae* or high intraspecific variability. Unfortunately, DNA sequence data of *Z. novae-zelandiae* are not available to provide answers to these hypotheses.

Our knowledge about the biogeography of *Zanclospora* is fragmentary due to the lack of records verified by molecular data and its extreme scarcity. Published data of members of *Zanclospora* suggest a worldwide geographical distribution. Species were recorded from the tropics of Brazil, Brunei, Cuba, India, Ecuador, Ivory Coast, Kenya, Nigeria, Seychelles, Taiwan and Vietnam, but also from the temperate and subtropical climate zones of the Southern and Northern Hemispheres in Japan, New Zealand, South Africa, Spain and the USA [1,2,3,4,5,6,7,8,9,10,11,12,20,21,22,23,24,25,26,27,28]. Almost all published records were from decaying bark and wood, less often from fallen leaves, and were obtained by direct observation on natural substrates. Thus, it is unknown if these fungi also occur in related substrates such as soil or healthy plant tissues (as endophytes), where they remain overlooked due to their slow growth in culture and rarity.

To date, research of geographic distribution patterns of fungi has relied heavily on public nucleotide sequence databases such as NCBI GenBank [29] and UNITE [30], which enable blasting (BLASTn search) [31] against fungal barcodes generated by Sanger technology. Data mining of DNA barcodes is especially helpful for biogeography and diversity studies of abundant and globally distributed taxa e.g., [32]. However, most of the barcode sequences generated so far come from massively parallel sequencing technologies, whose data have been stored in various public repositories, not allowing for easy data mining in multiple studies. As a result, any biogeographic evaluation is laborious and limited to a small number of source datasets [33]. This gap has recently been filled by the creation of the GlobalFungi database of fungal ITS data [34,35] collected from terrestrial biomes of soil, dead or live plant material. Such a tool is particularly useful for studying members of the *Chaetosphaeriaceae*, which are usually less abundant and inhabit substrates covered by GlobalFungi.

This study aims to assess the systematic placement of *Zanclospora* and investigate intraspecific and interspecific variability of its members by using comparative morphology on natural substrates and in culture along with phylogenetic analyses. Other objectives include the description and experimental verification of teleomorph-anamorph connections and anamorphic phenotypes, and the determination of geographical distribution and ecology of species of *Zanclospora*.

## 2. Materials and Methods

### 2.1. Fungal Strains

During our study, we gathered several *Zanclospora* inhabiting decaying plant material in various localities from the south temperate climate zone of New Zealand, and north temperate climate zone of Europe in Portugal and Spain, and North America in the USA. Other specimens were obtained from the Fungarium of the Illinois Natural History Survey (ILLS, Champaign, IL, USA) and New Zealand Fungarium (PDD, Auckland, New Zealand). Holotypes and specimens collected in this study were deposited at CBS, ILLS and PDD (as dried voucher specimens or dried cultures).

Axenic cultures were derived from freshly collected material (see Section 2.2). Additional cultures were obtained from BCCM/MUCL Agro-food and Environmental Fungal Collection (MUCL, Université Catholique de Louvain, Louvain, Belgium), the International Collection of Microorganisms from Plants (ICMP, Auckland, New Zealand) and Westerdijk Fungal Biodiversity Institute (CBS, Utrecht, The Netherlands). Representative strains and ex-type strains isolated from our collections were deposited at CBS and ICMP.

Isolates, their sources and GenBank accession numbers of sequences generated in this study are listed in Table 1. Fungal novelties were registered in MycoBank.

### 2.2. Morphological Analysis

Morphological characteristics, i.e., anamorphic, synanamorphic and teleomorphic, were acquired from fungi growing on natural substrates and in culture. Ascomata, conidiophores and conidia from the natural substrates were rehydrated with tap water and examined with an Olympus SZX12 dissecting microscope (Olympus America, Inc., Melville, NY, USA,). Hand-sectioned ascomata, asci, ascospores and paraphyses, and conidiophores and conidia were mounted in 90% lactic acid, water or Melzer’s reagent. Measurements were taken in Melzer’s reagent. Means ± standard deviation (SD) based on a minimum of 20–25 measurements were given for sizes of asci, ascospores and conidia. Microscopic structures were examined using an Olympus BX51 compound microscope with differential interference contrast (DIC) and phase-contrast (PC) illumination. Images of microscopic structures were captured with an Olympus DP70 camera operated by Imaging Software Cell^D (Olympus). Macroscopic images of colonies were documented using a Canon EOS 77D digital camera with Canon EF 100mm f/2.8L Macro IS USM objective (Canon Europe Ltd., Middlesex, UK) with daylight spectrum 5500K 16W LED lights. All images were processed with Adobe Photoshop CS6 (Adobe Systems, San Jose, CA, USA).

Single and multiple ascospore and conidial isolates were obtained from fresh material with the aid of a single-spore isolator (Meopta, Prague, Přerov, Czech Republic) and incubated on water agar or Modified Leonian’s agar (MLA) [36] at a temperature of 20–25 °C. Strains were inoculated in triplicate on cornmeal dextrose agar (CMD) (17 g of cornmeal agar Oxoid Limited, United Kingdom, Hampshire, 2 g of dextrose, 1 L of distilled water, sterilized for 15 min at 121 °C), MLA, oatmeal agar (OA) [37] and potato-carrot agar (PCA) [38]. Descriptions of colonies were based on 4–6-week-old cultures grown in darkness at 22–23 °C. Strains were also inoculated on cornmeal agar (CMA) [38] and OA with sterile stems of *Urtica dioica* to induce sporulation.

### 2.3. DNA Extraction, Amplification and Sanger Sequencing

Protocols for the DNA extraction and amplification of samples with ILLS and S.M.H. prefixes followed [39,40]. Processing of samples with M.R., ICMP and CBS prefixes followed [41,42]. Other samples were processed according to [9]. Automated sequencing was carried out by Eurofins GATC Biotech Sequencing Service (Cologne, Germany), the WM Keck Center at the University of Illinois Urbana-Champaign (Champaign, IL, USA) and Westerdijk Fungal Biodiversity Institute (Utrecht, The Netherlands). Raw sequence data were analysed using Sequencher v.5.4.6 (Gene Codes Corp, Ann Arbor, MI, USA).

### 2.4. Gene Markers, Sequence Alignments and Phylogenetic Analyses of Fungal Strains

Sequences of six gene markers: ITS1-5.8S-ITS2 (ITS) of the nuclear rRNA cistron, the small subunit 18S ribosomal DNA gene (18S) and the large subunit 28S ribosomal DNA gene (28S) (approximately 1800 base pairs at the 5′-end), domains 5–7 of the second largest subunit of RNA polymerase II (*rpb2*), the intermediate section of the coding region of the translation elongation factor 1-alpha (*tef1-α*) and coding and non-coding regions of beta-tubulin (*tub2*) marked by exons 2−6, were analysed to assess evolutionary relationships of *Zanclospora* and similar fungi. GenBank accession numbers for sequences retrieved from GenBank and published in other studies [9,39,41,42,43,44,45,46,47,48,49,50,51,52,53,54,55,56,57,58,59,60,61,62,63,64,65,66,67,68,69,70,71,72,73,74,75] are listed in Table S1.

Sequences were aligned manually in Bioedit v.7.1.8 [76] and introns were excluded from the alignments. The sequences were combined into four datasets that were partitioned into ITS, 18S, 28S, *rpb2*, *tef1-α* and coding and non-coding regions of *tub2* subsets of nucleotide sites for which we assumed rate heterogeneity. Single-locus data sets were evaluated using PartitionFinder2 [77], implemented in the CIPRES Science Gateway v.3.3 [78,79], to find the best partitioning scheme for our datasets and to select best-fit models under corrected Akaike information criteria. Conflict-free data sets were concatenated, and four alignments (deposited in TreeBASE) were subjected to subsequent phylogenetic analyses. 

Since there are no previous phylogenetic studies on *Zanclospora* and the standard use of particular nuclear loci vary among fungal groups, we conducted four phylogenetic analyses to assess relationships of the genus, based on the preliminary results of the BLASTn search. The phylogenetic analysis of *Zanclospora* and members of the Sordariomycetes were based on 18S, 28S and *rpb2* markers. The relationships within the *Chaetosphaeriaceae* were assessed with the ITS and 28S sequences. The intraspecific relationships of *Zanclospora* were evaluated with ITS, 28S, *tef1-α* and *tub2* genes, and the phylogenetic analysis of two *Zanclospora* strains with affinity to the Xylariales were assessed in the analysis of the combined ITS, 28S, *tef1-α* and *rpb2* sequences.

Phylogenetic reconstructions were performed using Bayesian Inference (BI) and Maximum Likelihood (ML) analyses through the CIPRES Science Gateway v.3.3. ML analyses were conducted with RAXML-HPC v.8.2.12 [80] with a GTRCAT approximation. Nodal support was determined by non-parametric bootstrapping (BS) with 1000 replicates. BI analyses were performed in a likelihood framework as implemented in MrBayes v.3.2.6 [81]. Two Bayesian searches were performed using default parameters. The B-MCMCMC analyses lasted until the average standard deviation of split frequencies was below 0.01 with trees saved every 1000 generations. The first 25% of saved trees, representing the burn-in phase of the analysis, were discarded. The remaining trees were used for calculating posterior probabilities (PP) of recovered branches. The BI and ML phylogenetic trees were compared visually for a topological conflict among supported clades.

Histograms of intraspecific and interspecific distances of *Zanclospora* s. str. were created for each of the four markers (ITS, 28S, *tef1-α,* and *tub2*) used in the phylogenetic analyses in order to illustrate the amount of overlap for each gene. Matrix of pairwise distances was computed with MEGAX [82] using the Kimura two parameters (K2P) model, and the histogram was plotted in GraphPrism 7.03 software (Graphpad Software Inc., LaJolla, CA, USA) using a bin size of 0.001.

### 2.5. Phylogeny of Environmental Sequences and Biogeography

Initially, the interspecies genetic distance (p-dist) was calculated for ITS1 and ITS2 datasets of 12 *Zanclospora* species using MEGAX [82] to obtain sequence similarity thresholds for species delimitation in *Zanclospora*. The obtained value and the limit of full coverage were used for the search in the GlobalFungi v.0.9.6 (release version 1.0) database containing data from 20,000 samples originating from 207 studies [34]. For each taxon, data about occurrence across environmental samples and metadata related to the particular samples (location, substrate, biome, climatic data, pH) were obtained (Table S2). Taxa related to *Zanclospora* were used for comparison, e.g., *Chaetosphaeria minuta*, *Cryptophiale, Cryptophialoidea* and *Kionochaeta* (Table S3).

In order to study *Zanclospora* diversity hidden among environmental sequences, the full-length ITS1 and ITS2 sequences of 12 *Zanclospora* species were blasted against the GlobalFungi database. The sequences with a similarity of 89–100% and full-length coverage were downloaded. The *Zanclospora* genus boundaries were inferred from ML trees of ITS1 and ITS2 sequences computed in Phyml v.3.1 [83] using the GTR model and 500 bootstrap replicates. The same procedure, i.e., blasting, downloading of related sequences and phylogenetic analyses, was performed against sequences deposited in NCBI GenBank and UNITE database. Virtual taxa, consisting of environmental sequences only, were defined as arbitrary phylotypes in the ML phylogenetic trees. Metabarcoding data can contain pseudogenous copies, which may lead to an overestimation of diversity. Thus, GC-content and ITS2 secondary structure stability of obtained sequences were compared as recommended in [84].

## 3. Results

### 3.1. Phylogenetic Analyses

**Figure 1:**
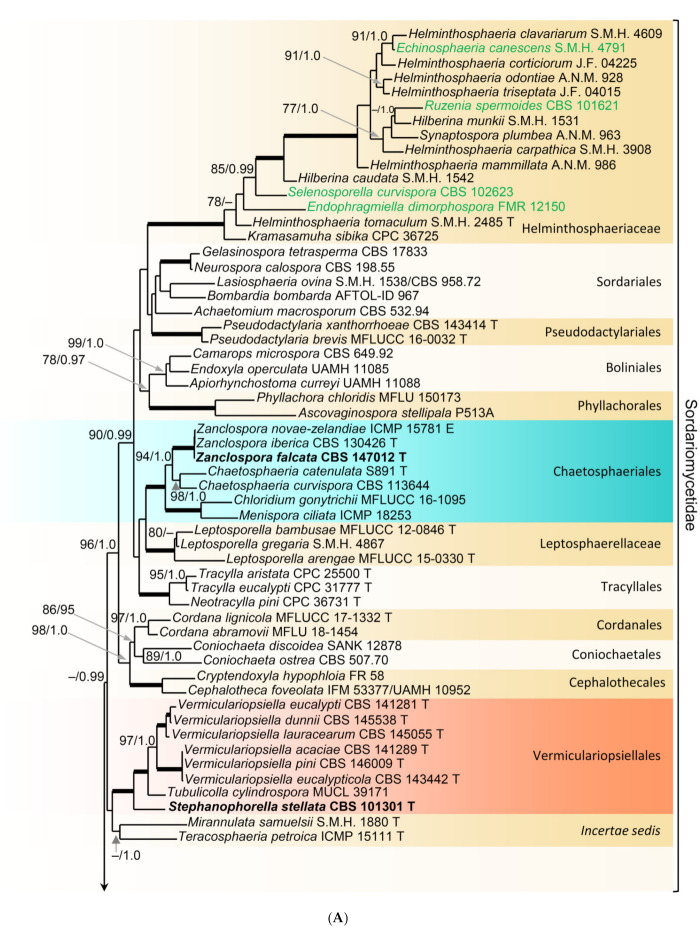

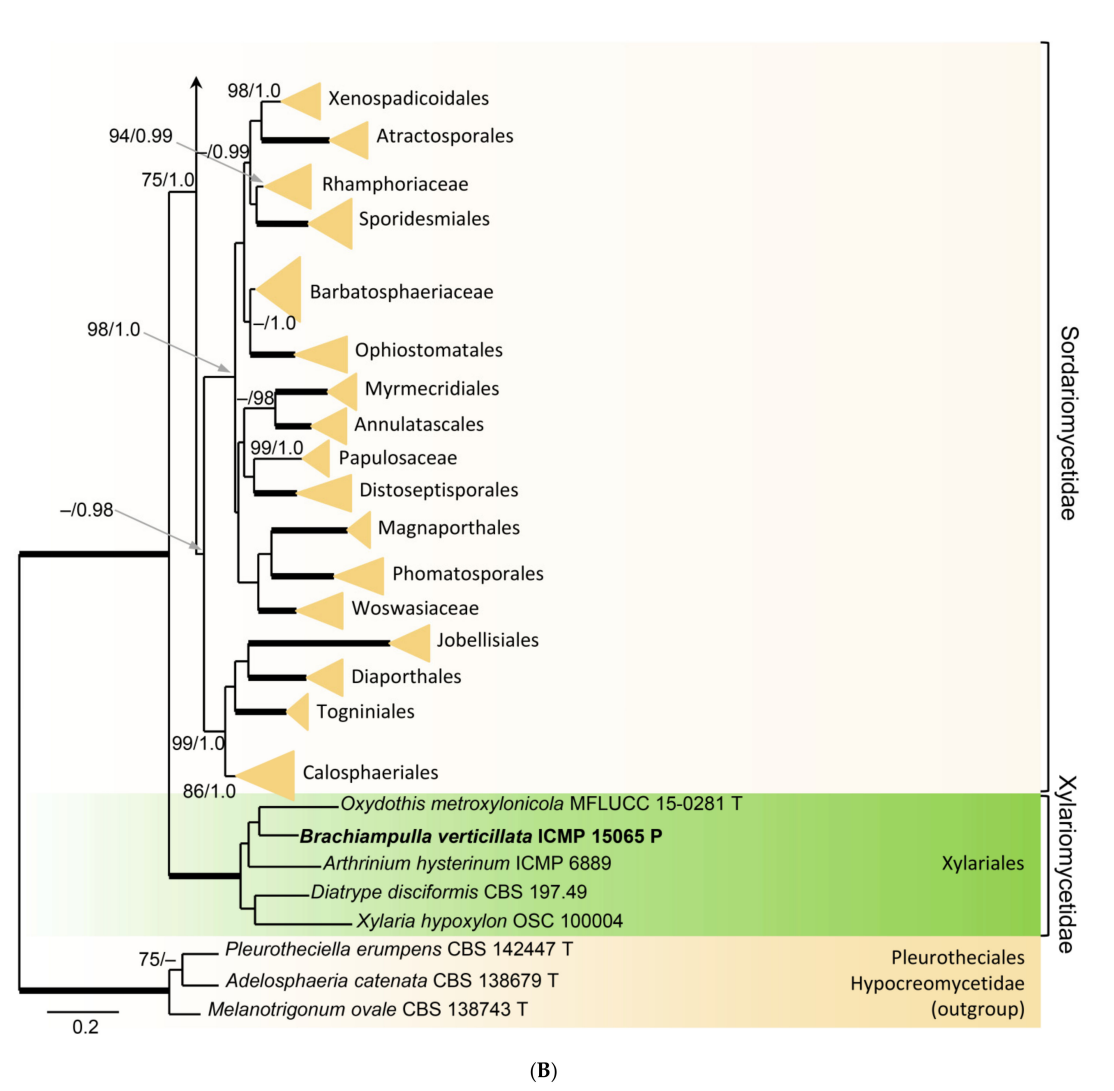
(**A**) Phylogenetic analysis of 18S, 28S and *rpb2* of the Sordariomycetes. Species names given in bold are taxonomic novelties; T, E and P indicate ex-type, ex-epitype and ex-paratype strains. Taxa highlighted in green represent *Selenosporella* and selenosporella-like fungi in the Helminthosphaeriaceae. Thickened branches indicate branch support with maximum likelihood (ML) bootstrapping (BS) = 100%, posterior probabilities (PP) values = 1.0. Branch support of nodes ≥75% ML BS and ≥0.95 PP is indicated above or below branches. (**B**) Phylogenetic analysis of 18S, 28S and *rpb2* of the Sordariomycetes (continued). For legend refer to (**A**).

In order to examine the evolutionary relationships of *Zanclospora* within the Sordariomycetidae, phylogenetic analysis was based on the combined 18S, 28S and *rpb2* sequences of 108 representatives of the Sordariomycetes. *Adelosphaeria catenata*, *Melanotrigonum ovale* and *Pleurotheciella erumpens* (Pleurotheciales, Hypocreomycetidae) served as the outgroup. One hundred-twenty nucleotides (nt) at the 5′-end of 18S, 85 nt at the 5′-end and 483 nt at the 3′-end of 28S were excluded from the alignment because of the incompleteness in the majority of sequences. The full dataset consisted of 4139 characters including gaps (18S = 1634 characters, 28S = 1342, *rpb2* = 1163) and 2213 unique character sites (RAxML). For the BI analysis, the GTR+I+G model was selected for all partitions. The BI and ML trees were not in conflict; the ML tree is shown in Figure 1. The subclass Sordariomycetidae included 30 well-supported clades (≥75% ML BS/≥1.0 PP) representing orders and families and one *incertae sedis* lineage. This subclass was resolved with four major subclades. The first subclade (96/1.0) included ten orders and families with mostly phialidic and tretic conidiogenesis, rarely holoblastic, namely Boliniales, Cephalothecales, Chaetosphaeriales, Coniochaetales, Cordanales, Helminthosphaeriaceae, Leptosphaerellaceae, Phyllachorales, Pseudodactylariales, Sordariales and Tracyllales. The second subclade included Vermiculariopsiellales (100/1.0) and an unsupported clade with *Mirannulata samuelsii* and *Teracosphaeria petroica* (both *incertae sedis*). Species with prevalent holoblastic conidiogenesis, if known, attributed to 13 orders and families, formed a strongly supported subclade (98/1.0), which was inferred as sister to the fourth subclade (99/1.0) containing Calosphaeriales, Diaporthales, Jobellisiales and Togniniales with phialidic conidiogenesis. The Xylariomycetidae were resolved as a strongly supported clade (100/1.0) encompassing five representatives of the Xylariales. *Zanclospora novae-zelandiae* clustered in the Chaetosphaeriales (100/1.0), while *Z. stellata* nested in the Vermiculariopsiellales and was transferred to a new genus *Stephanophorella*. *Zanclospora urewerae* was inferred as a member of the Xylariales (100/1.0) and accommodated in the new genus *Brachiampulla*. A non-type strain of *Selenosporella curvispora* CBS 102623, the generic type, clustered in the Helminthosphaeriaceae.

Relationships of *Zanclospora* with four *Chaetosphaeria*, so far known to produce only a phaeostalagmus-like anamorph or their anamorph is unknown [12,85,86], and other members of the *Chaetosphaeriaceae* were assessed in the phylogenetic analysis of a data set that included ITS and 28S sequences of 89 representative species of the family. *Leptosporella arengae* and *L. bambusae* (Leptosphaerellaceae), and *Tracylla eucalypti* and *T. aristata* (Tracyllaceae) served as an outgroup. Seventy-one nt at the 5′-end and 663 nt at the 3′-end of 28S were excluded from the alignment. 

**Figure 2:**
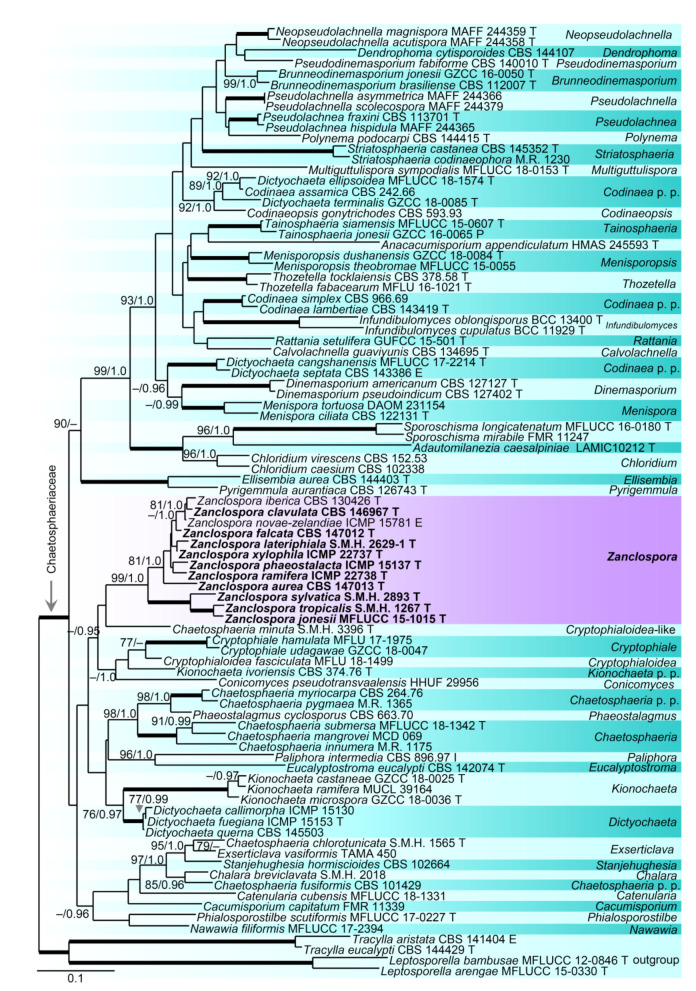
Combined phylogeny using ITS and 28S of members of the *Chaetosphaeriaceae*. Species names given in bold are taxonomic novelties; T, E, I and P indicate ex-type, ex-epitype, ex-isotype and ex-paratype strains. Thickened branches indicate branch support with ML BS = 100%, PP values = 1.0. Branch support of nodes ≥75% ML BS and ≥0.95 PP is indicated above or below branches. Abbreviation: p.p. after a genus name (pro parte).

The alignment had 1722 characters including gaps (ITS = 605 characters, 28S = 1117) and 861 unique character sites (RAxML). For the BI analysis, the GTR+I+G model was selected for both partitions. No conflicts occurred between BI and ML trees; the ML tree is shown in Figure 2. The *Chaetosphaeriaceae* included 47 lineages representing genera or natural groups of species. *Zanclospora* was resolved as a strongly supported monophyletic clade (99/1.0). Four *Chaetosphaeria*, namely *Ch. jonesii*, *Ch. phaeostalacta*, *Ch. sylvatica* and *Ch. tropicalis*, clustered in the *Zanclospora* clade. *Kionochaeta* was shown as paraphyletic, a non-type strain of *K. ramifera* MUCL 39164 [87], the type species of the genus, clustered with two other *Kionochaeta* as a monophyletic lineage (100/1.0), while *K. ivoriensis* nested on a separate branch close to *Cryptophiale* and *Cryptophialoidea*. *Phaeostalagmus* (as *P. cyclosporus* CBS 663.70, the generic type) and *Stanjehughesia* (as *S. hormiscioides* CBS 102664), two hyphomycete genera whose similar phenotypes appear in the life cycle of *Zanclospora*, were resolved as separate lineages.

**Figure 3:**
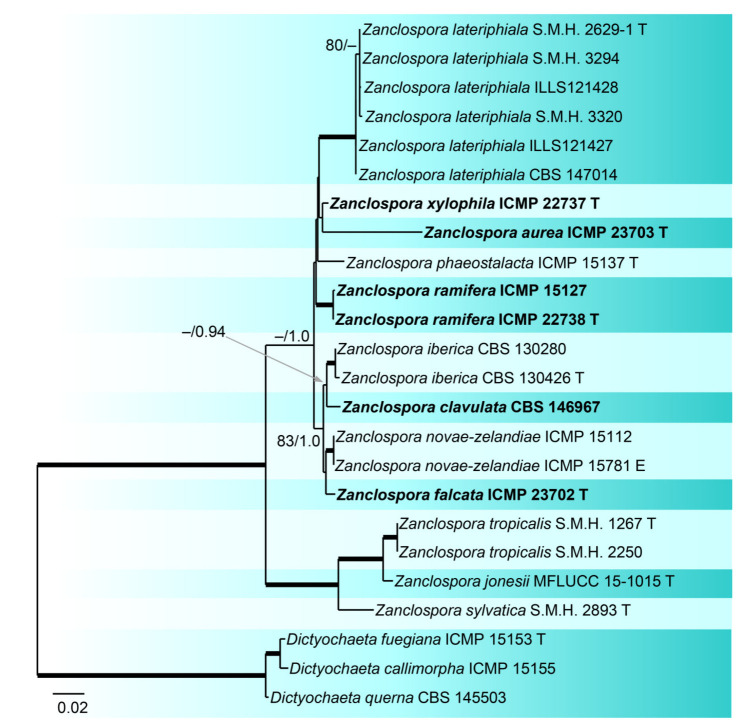
Combined phylogeny of ITS, 28S, *tef1-α* and *tub2* sequences of *Zanclospora*. Names given in bold are new species. T and E indicate ex-type and ex-epitype strains. Thickened branches indicate branch support with ML BS = 100%, PP values = 1.0. Branch support of nodes ≥75% ML BS and ≥0.95 PP is indicated above or below branches.

In order to evaluate relationships among 16 strains of *Zanclospora* and five strains of *Chaetosphaeria*, some of which form a phaeostalagmus-like anamorph in culture, we analysed a data set of the combined ITS, 28S, *tef1-α* and *tub2* sequences. Three *Dictyochaeta* were used as an outgroup to root the tree. The alignment had 4770 characters including gaps (ITS = 487, 28S = 1842, *tef1-α* = 992, *tub2* = 1449) and 729 unique character sites (RAxML). For the BI analysis, the GTR+G model was selected for ITS and *tef1-α*, GTR+I+G for 28S and *tub2* coding region, and GTR+I for the *tub2* non-coding partition. The ML tree is shown in Figure 3. *Zanclospora* was resolved with two subclades containing 12 species. Molecular data confirmed a close relationship among species with the typical *Zanclospora* conidiophores and those exhibiting phaeostalagmus- and stanjehughesia-like morphotypes. The first subclade (–/1.0) comprised nine species including *Z. novae-zelandiae* and five new species, namely *Z. aurea*, Z. *clavulata, Z. falcata*, *Z. ramifera* and *Z. xylophila*, described below. The second subclade (100/1.0) contained three species formerly attributed to *Chaetosphaeria*. The differences between BI and ML trees were in the position of several species. In the BI tree, *Z. aurea* was shown on a separate branch, and *Z. phaeostalacta* and *Z. xylophila* were resolved as sister species.

**Figure 4:**
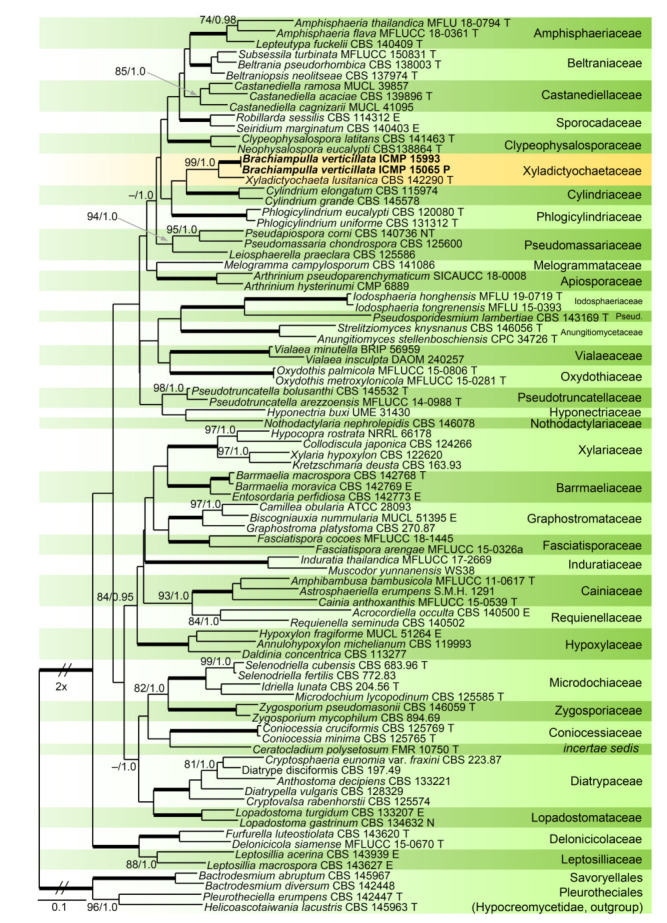
Combined phylogeny of ITS, 28S, *tef1-α* and *rpb2* sequences of selected members of the Xylariales. Species names given in bold are taxonomic novelties; T, E, N and P indicate ex-type, ex-epitype, ex-neotype and ex-paratype strains. Thickened branches indicate branch support with ML BS = 100%, PP values = 1.0. Branch support of nodes ≥ 75% ML BS and ≥ 0.95 PP is indicated above or below branches. Abbreviation: Pseud. (Pseudosporidesmiaceae).

Relationships of *Z. urewerae* were assessed in the phylogenetic analysis of the combined ITS, 28S, *tef1-α* and *rpb2* sequences of 81 representatives of the Xylariales. *Bactrodesmium abruptum* and *B. diversum* (Savoryellaceae), *Helicoascotaiwania lacustris* and *Pleurotheciella erumpens* (Pleurotheciaceae) were used to root the tree. Eighty-five nt at the 5′-end and 925 nt at the 3′-end of 28S were excluded from the alignment. The alignment had 3964 characters including gaps (ITS = 764 characters, 28S = 857, *tef1-α* = 1148, *rpb2* = 1195) and 2307 unique character sites (RAxML). For the BI analysis, the SYM+I+G model was selected for ITS, while the GTR+I+G model was selected for 28S, *tef1-α* and *rpb2* partitions. There were no conflicts between ML and BI trees; the ML tree is shown in Figure 4. The Xylariales included 33 well-supported clades representing families and one *incertae sedis* lineage. *Zanclospora urewerae* was clustered as a sister to *Xyladictyochaeta* of the Xyladictyochaetaceae (99/1.0). Morphologically similar genera and species such as *Selenodriella* (Microdochiaceae) and *Ceratocladium polysetosum* (*incertae sedis*) formed separate lineages.

### 3.2. Barcode Analysis 

Although we lacked enough representatives for species to be able to fully explore the barcoding gap of *Zanclospora* s. str., we used the current four-gene dataset to examine pairwise genetic distances, visualize them and evaluate the species-delimiting ability of each marker (Table S4). The barcoding gap separating intraspecific and interspecific variability of *Zanclospora* was present in all studied markers, with the biggest gap found in *tef1-α*, followed by *tub2*, ITS and 28S. In ITS, the minimal interspecific divergence occurred among species of the *Z. novae-zelandiae* species complex (0.64–1.3%), the next minimum and maximum distances between other species ranged between values 1.3–16.18%. In *tef1-α*, the minimum genetic distance occurred between the sibling species, *Z. falcata* and *Z. novae-zelandiae* (0.52%), but ranged from 1.05 to 5.24% in other species. The situation in *tub2* gene was complicated by various lengths of the sequenced fragments. Nonetheless, the performance of the gene is comparable to *tef1-α* and delimits closely related species; the minimum and maximum interspecific distances ranged between values 1.45–15.14%. The genes *tef1-α*, *tub2* and ITS are recommended as barcodes for *Zanclospora*.

### 3.3. Analysis of Zanclospora Diversity in Environmental Samples, Biogeography and Ecology

For the ITS1, the lowest interspecies distance was 0.012 (*Z. clavulata* vs *Z. novae-zelandiae*). For the ITS2, the lowest distance ranged from 0 (*Z. novae-zelandiae* vs *Z. xylophila*) followed by *Z. novae-zelandiae* vs *Z. iberica* and *Z. xylophila* vs *Z. iberica*, both 0.012. The obtained values showed that for ITS1 and ITS2 there is no generally valid sequence similarity threshold for species delimitation in *Zanclospora*. However, 99–100% sequence similarity was applicable for most of the species and was used for the search in GlobalFungi. The only exceptions were *Z. novae-zelandiae* and *Z. xylophila*, where the criterion of full sequence similarity in ITS2 was used.

**Figure 5:**
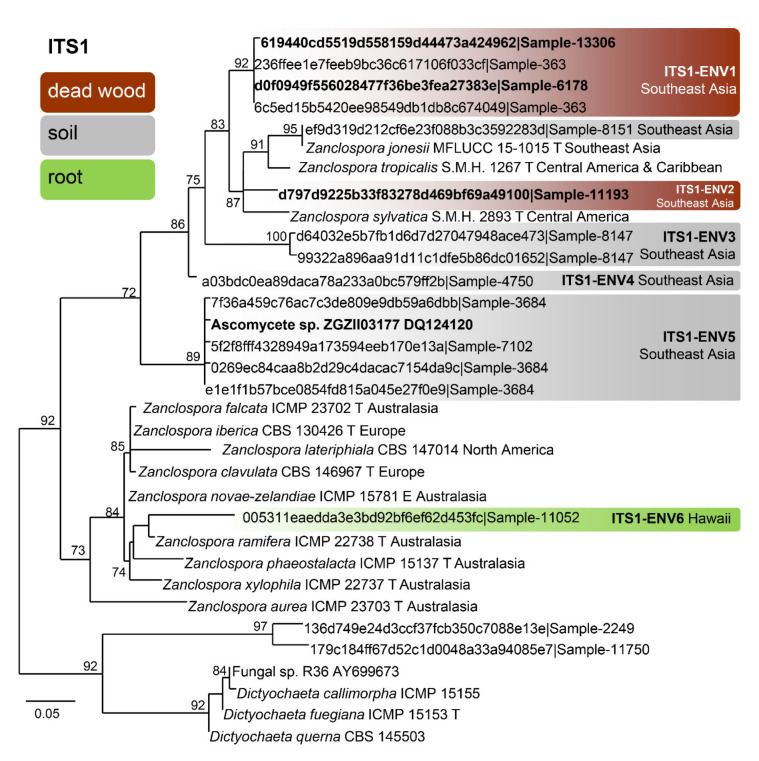
Phylogenetic relationships among *Zanclospora* species and related ITS1 environmental sequences deposited in the GlobalFungi database. The titles of sequences contain the sequence and sample codes taken from GlobalFungi. Branch support (ML) is retained at the nodes. Environmental samples in bold indicate those sequenced for the whole ITS region. Habitat of the environmental sequences is indicated by brown, grey and green boxes corresponding to dead wood, soil or roots.

**Figure 6:**
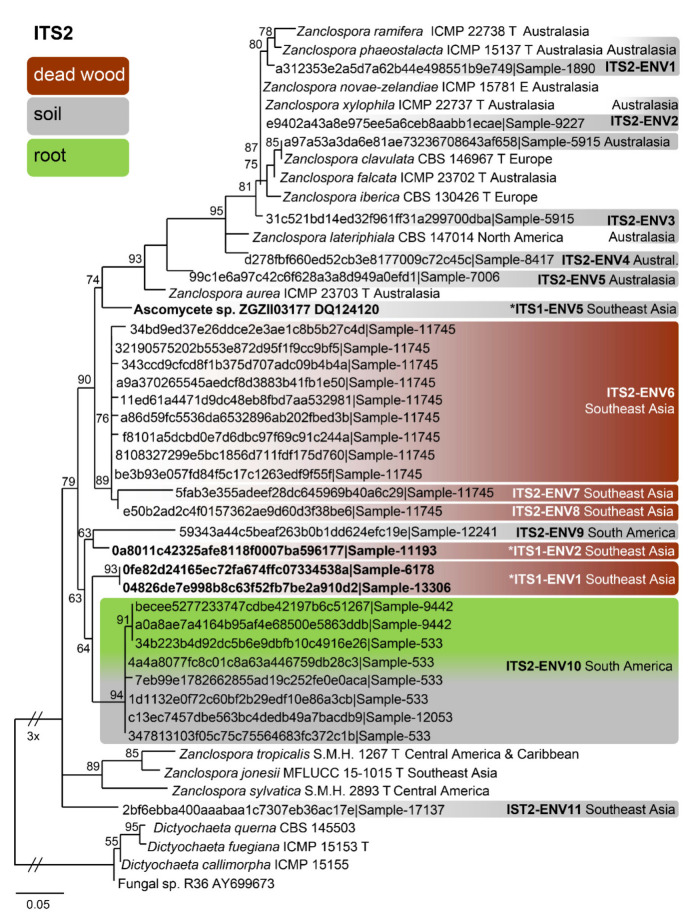
Phylogenetic relationships among *Zanclospora* species and related ITS2 environmental sequences deposited in the GlobalFungi database. The titles of sequences contain the sequence and sample codes taken from GlobalFungi. Branch support (ML) is retained at the nodes. Environmental samples in bold indicate those sequenced for the whole ITS region. Habitat of the environmental sequences is indicated by brown, grey and green boxes corresponding to dead wood, soil or roots. Asterisk (*) indicates phylotypes that can be linked with phylotypes defined by the ITS1 marker.

The BLAST search resulted in 559 unique ITS1 sequences (similarity 89–100% to *Zanclospora* queries, see Section 2.5). The dereplicated dataset had 33 sequences, 185 characters, from which 82 were variable and 16 singletons. The ML tree (Phyml) was rooted in a branch leading to the *Dictyochaeta* clade and is shown in Figure 5. The environmental sequences were clustered into seven phylotypes. Among them, one can be linked with *Z. jonesii*. For the ITS2, 108 unique sequences were found, which resulted in 79 sequences attributable to *Zanclospora*. The dereplicated dataset had 48 sequences, 166 characters, from which 87 were variable and 20 singletons. The ML tree (Phyml) was rooted in a branch leading to the *Dictyochaeta* clade and is shown in Figure 6. The environmental sequences clustered into 15 phylotypes. One was linked with *Z. clavulata*, while the other three contained sequences of the whole ITS region, and thus may be linked with phylotypes defined by the ITS1 marker. The same procedure was applied to data from the NCBI GenBank and UNITE databases and resulted in the single sequence (Ascomycete sp., DQ124120, unpublished), which was linked with phylotype ITS1-ENV5 (Figure 5). The sequence similarity in the ITS2 region has little differentiation power in the *Z. novae-zelandiae* clade. Interestingly, *Z. novae-zelandiae* and *Z. xylophila* share identical ITS2, while they are distinct in ITS1 and other studied genes. The best hit of *Z. novae-zelandiae*/*Z. xylophila* was 99.32% and is considered as ITS2-ENV2 phylotype (Figure 6, Table S3).

**Figure 7:**
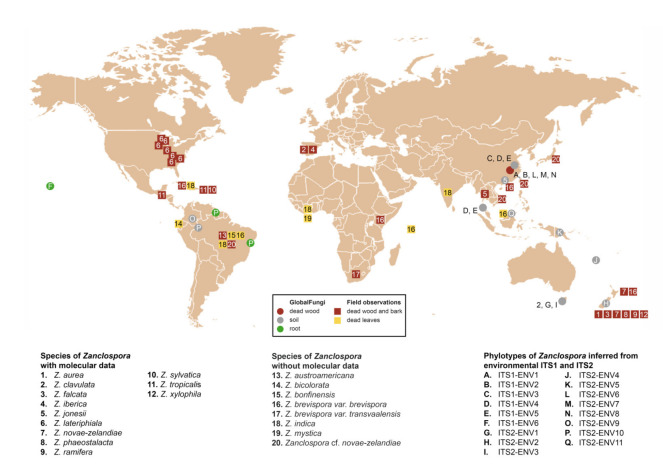
Geographical distribution and substrate affinity of *Zanclospora* species with known ITS sequence data. The map summarizes data from the GlobalFungi database (shown by circles) and field collections verified by sequencing (species 1–12) or based only on published data (species 13–20) (shown by squares). See Tables S2 and S6 for primary data. Each symbol (circle or square) represents a unique sample. The substrates are differentiated by colours. Note that environmental taxa defined by ITS1 sequences can overlap with those from the ITS2 dataset.

Concerning the diversity presented in environmental sequences, only *Z*. *clavulata* and *Z. jonesii* were traced in the GlobalFungi database at the defined similarity level. Another six and 11 phylotypes, respectively, roughly corresponding to the level of species, were identified in ITS1 and ITS2. Biogeography and ecology of newly identified phylotypes inferred from the GlobalFungi database (Tables S2, S3 and S5) and known species (Table S6) are summarised in Figure 5, Figure 6 and Figure 7.

Based on the field records verified or unverified by DNA data, two centres of *Zanclospora* diversity can be identified: South, Central America and Caribbean (*Z. sylvatica*, *Z. tropicalis*, *Z. austroamericana*, *Z. bicolorata*, *Z. bonfinensis*, *Z. brevispora* var. *brevispora*, *Z. indica*, *Z.* cf. *novae-zelandiae*) and Australasia (*Z. aurea*, *Z. falcata*, *Z. novae-zelandiae*, *Z. phaeostalacta*, *Z. ramifera*, *Z. xylophila*, *Z. brevispora* var. *brevispora*), which are followed by regions with lesser diversity such as Africa (*Z. brevispora* var. *brevispora*, *Z. brevispora* var. *transvaalensis*, *Z. mystica*), Southeast Asia (*Z. jonesii*, *Z.* cf. *novae-zelandiae*), Europe (*Z. clavulata*, *Z. iberica*) and North America (*Z. lateriphiala*).

Interestingly, the observed geographical distribution corresponds roughly with the phylogenetic relatedness (Figure 3, Figure 5 and Figure 6). In the four-gene analysis, *Zanclospora* formed two main clades. A clade containing *Z. novae-zelandiae* and related species (Figure 3, node –/1.0) has Australasian distribution, with two species (*Z. clavulata*, *Z. iberica*) known from Europe and one (*Z. lateriphiala*) from the USA. In the second clade, there are clustered two strains from Central America and the Caribbean (*Z. sylvatica*, *Z. tropicalis*) together with a strain from Southeast Asia (*Z. jonesii*). Analyses of the environmental sequences identified another diversity centre in Southeast Asia (China, Malaysia: ITS1-ENV1–5, ITS2-ENV6–8, 11), followed by Australasia (New Caledonia, New Zealand, Papua New Guinea and Tasmania: ITS2-ENV1-5), South America (Brazil, Colombia and French Guiana: ITS2-ENV9, 10) and Hawaii (ITS1-ENV6). The rest of the lineages in the ITS2 tree (Figure 6) are distributed either in Central or South America or Southeast Asia. *Zanclospora* species/phylotypes were represented by only 296 sequence reads out of a total of 6.5 × 10^8^ reads (ITS1 64%, ITS2 36%) deposited in the GlobalFungi database. All hits originated from samples collected below the latitude of 23° N. In Europe (44% of all samples in GlobalFungi) and North America (20%), which are the best-sampled continents in the GlobalFungi database, these fungi were completely missing. The field collections originated mostly from decaying bark and wood or leaf litter in terrestrial, less often in freshwater habitats (Figure 7, Table S6). In the case of the environmental sequences, the most frequently inhabited substrate appeared to be bulk soil, followed by dead wood and roots in the forest and rarely shrubland biome. The median values of mean annual temperature and mean annual precipitation were 16 °C and 2223 mm. The sampling sites belonged to the biomes of temperate or tropical rainforests (Table S2).

### 3.4. Taxonomy

***Zanclospora*** S. Hughes & W.B. Kendr., N. Z. J. Bot. 3: 151. 1965.

Type species: *Zanclospora novae-zelandiae* S. Hughes and W.B. Kendr., N. Z. J. Bot. 3: 152. 1965.

Emended description: Colonies effuse, hairy, golden-yellow, yellow-brown, tawny, reddish-brown or dark brown with white to light straw conidial masses, sometimes accompanied by ascomata. Teleomorph: Ascomata perithecial, non-stromatic, glabrous, papillate. Ostiole periphysate. Ascomatal wall 2–3-layered. Paraphyses disintegrating with age, hyaline, branched, septate. Asci unitunicate, 8-spored, short-stipitate, ascal apex with a non-amyloid, refractive apical annulus. Ascospores transversely septate, hyaline. Anamorph: Conidiophores macronematous, mononematous, erect, setiform, cylindrical to cylindrical-fusiform, septate, brown, paler towards the apex, occasionally dark brown and opaque, apex acute, subacute or obtuse, sterile or developed into a phialide, conidiophores simple or branched; branches setiform, fertile, resemble the main stalk or shorter, sterile inserted into the conidiophore. Conidiogenous cells monophialidic, determinate, sessile, discrete, lateral, appressed to the conidiophore, arise just below the septa, arranged in whorls forming one or two compact fertile regions, occasionally integrated, terminal, at the conidiophore apex, ovoid to lageniform, subhyaline to light brown, smooth, collarettes inconspicuous to short-flared. Macroconidia falcate, almost horseshoe-shaped, obovoid, occasionally bacilliform, straight, gently or strongly curved, aseptate, hyaline, smooth, without setulae or sheaths, accumulating in a slimy mass. Microconidia (formed only in culture) clavate to oblong-clavate, ellipsoidal to fusiform, aseptate, hyaline, smooth. Synanamorphs: phaeostalagmus-like (formed only in culture). Conidiophores semimacronematous or macronematous, mononematous, erect, simple or branched, septate, pigmented, apex fertile. Conidiogenous cells phialidic with a single apical opening, integrated, terminal or discrete, lateral, solitary or in whorls. Conidia ellipsoidal to oblong, aseptate, hyaline, smooth. stanjehughesia-like. Conidiophores macronematous, mononematous, erect, unbranched, occasionally branched, septate, pigmented, straight or sinuous, sterile, tapering towards the base, gradually tapering towards the apex, sterile, rarely with a few lateral, subhyaline, lageniform phialides.

Habitat and geographical distribution: Members of the genus are saprobes on decaying plant material with a worldwide distribution in Northern and Southern temperate, subtropical and tropical climate zones (Figure 7). Although most of the field observations include specimens on decaying wood or fallen leaves, environmental ITS1 and ITS2 sequences attributable to *Zanclospora* originated also from soil and roots (Tables S2 and S5). Moreover, environmental data suggested several new, likely undescribed species lineages from Southeast Asia, Australasia and South America.

Notes: Our *Zanclospora* strains derived from conidia and ascospore isolates exhibit an undescribed morphological variability in anamorphic characteristics that are associated with three anamorphic stages. Sterile or rarely fertile conidiophores resembling euseptate, cylindrical conidia of another hyphomycete *Stanjehughesia* [18] were often associated with ascomata and the typical *Zanclospora* conidiophores. On natural substrates and in culture, *Zanclospora* and stanjehughesia-like conidiophores occur irregularly and independently of each other. The *Zanclospora* conidiophores that arise on agar are usually less complex and significantly reduced in size becoming remarkably similar to *Phaeostalagmus* [19], or they are reduced to single conidiogenous cells. These reduced forms produce microconidia exclusively, compared to the complex *Zanclospora* conidiophores on natural substrates or sterile stems of *U. dioica* in vitro producing macroconidia. There is very little difference between the morphology of a reduced *Zanclospora* conidiophore and what can be called the phaeostalagmus-like morphotype. In the latter, the phialides are lateral, sessile, arranged in verticilli and also disposed terminally, sometimes on short branches, resembling *P. cyclosporus*, the type species of the genus. The phaeostalagmus-like morphotype occurs primarily in species whose axenic culture was derived from ascospores and the *Zanclospora* anamorph is unknown, i.e., *Z. phaeostalacta* ([85], Figures 55–61) *Z. sylvatica* ([12], Figures 203, 205, 206) and *Z. tropicalis* ([12], Figures 221, 222, 224).

**Figure 8:**
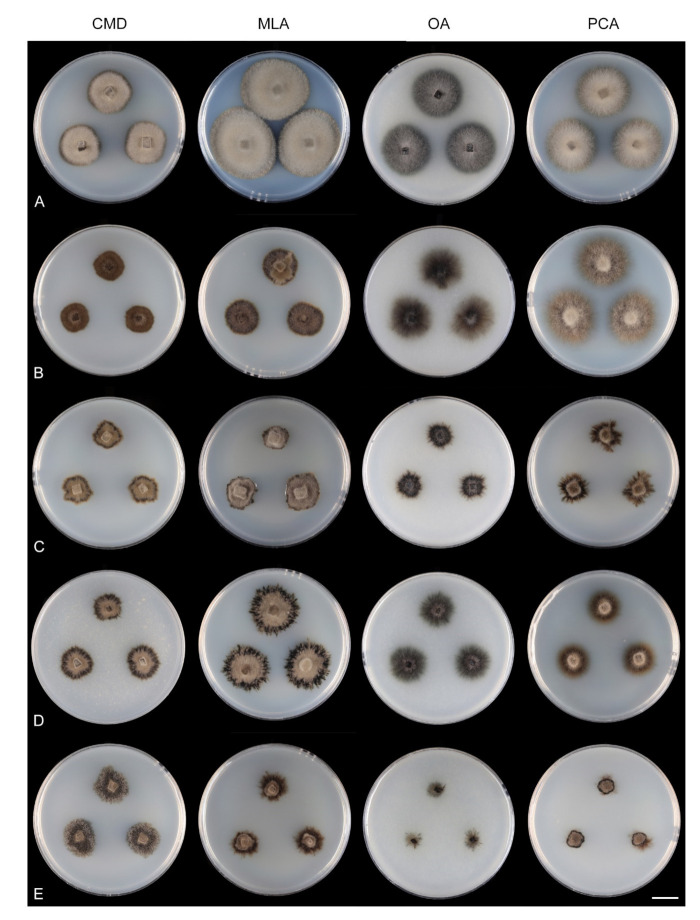
Colony morphology of *Zanclospora novae-zelandiae* species complex on cornmeal dextrose agar (CMD), Modified Leonian’s agar (MLA), oatmeal agar (OA) and potato-carrot agar (PCA) after 4 weeks. (**A**) *Z. clavulata* CBS 146967. (**B**) *Z. falcata* ICMP 23702. (**C**) *Z. iberica* CBS 130426. (**D**) *Z. iberica* CBS 130280. (**E**) *Z. novae-zelandiae* ICMP 15781. Bar: (**A**–**E**) = 1 cm.

**Figure 9:**
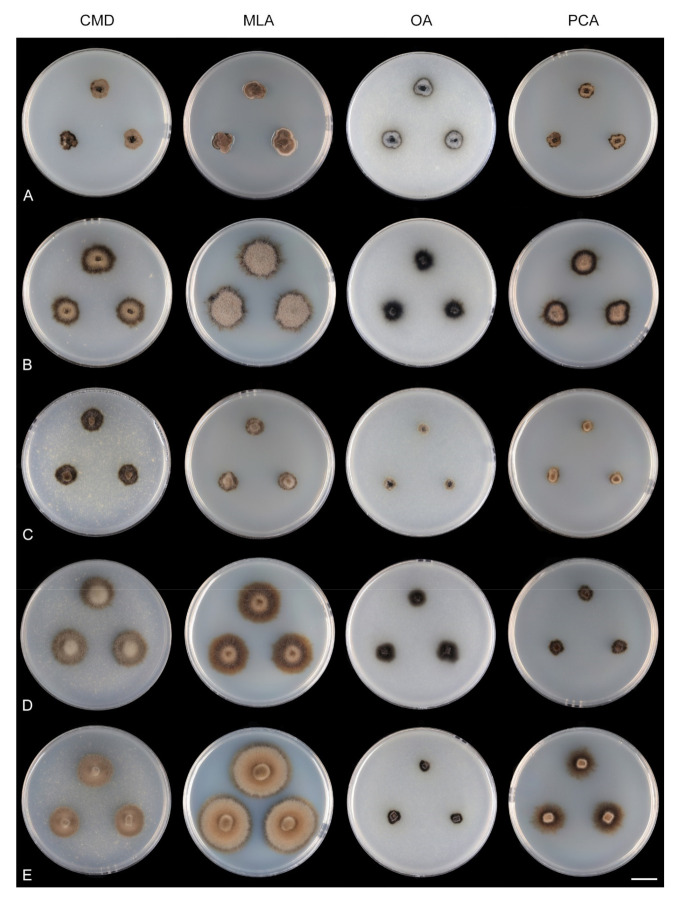
Colony morphology of *Zanclospora* on CMD, MLA, OA and PCA after 4(–6) weeks. (**A**) *Z. aurea* ICMP 23703 (6 weeks). (**B**) *Z. lateriphiala* CBS 147014 (4 weeks). (**C**) *Z. phaeostalacta* ICMP 15137 ex-type (4 weeks). (**D**) *Z. ramifera* ICMP 15127 (4 weeks). (**E**) *Z. xylophila* ICMP 22737. Bar: (**A**–**E**) = 1 cm.

Seventeen species and two varieties are accepted in *Zanclospora*, 12 of which have been verified using molecular data and are presented below. Their colonies on the four growth media are compared in Figure 8 and Figure 9. New teleomorph-anamorph connections have been experimentally confirmed for *Z. aurea*, *Z. falcata*, *Z. novae-zelandiae*, *Z. ramifera* and *Z. xylophila*. Five other species, whose molecular data are unavailable, are provisionally accepted in *Zanclospora* based on morphological similarities, namely *Z. austroamericana* [3], *Z. bicolorata* [10], *Z. bonfinensis* [8], *Z. brevispora* [1] and *Z. mystica* [4]. *Zanclospora indica* [2], *Z. stellata* [6] and *Z. urewerae* [7] are excluded from the genus.

*Zanclospora bonfinensis*, *Z. bicolorata* and *Z. mystica* deviate from other *Zanclospora* in conidiophores that are darker and opaque in the upper setiform part. Moreover, the two former species possess tubular to narrowly wedge-shaped collarettes and bacilliform to suballantoid conidia that are unique in *Zanclospora* and better correspond to *Z. stellata*, transferred to the new genus *Stephanophorella* in this study. The conidiophores of other *Zanclospora* are paler towards the apex, the apex and the rest of the conidiophore have more or less the same colour, conidia are falcate to almost horseshoe-shaped, obovoid, clavate and collarettes are inconspicuous to short-flared.

Preparation of the identification key has proven challenging, mainly due to inconsistencies in the occurrence of teleomorphs, anamorphs and synanamorphs on natural material and in culture. Therefore, a synopsis table with diagnostic features of accepted species of *Zanclospora* is compiled to show the interspecific variability (Table 2).

#### 3.4.1. Accepted Species

**Figure 10:**
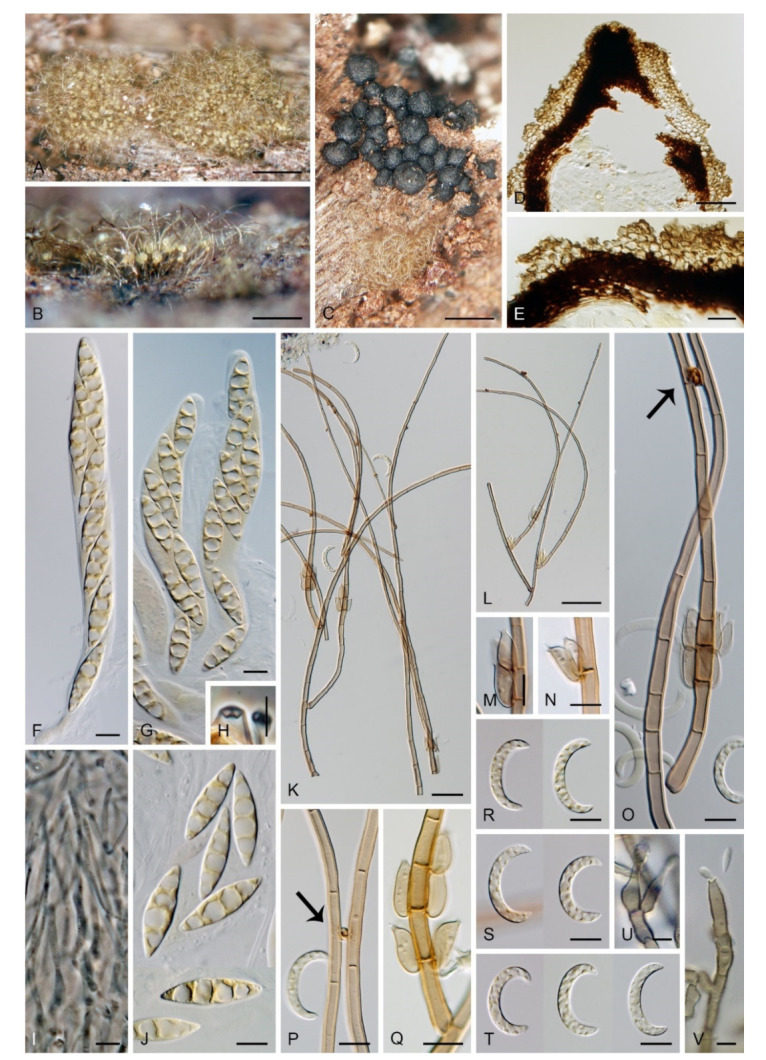
*Zanclospora aurea* (ICMP 23703 ex-type). (**A**,**B**) Colonies. (**C**) Ascomata associated with *Zanclospora* anamorph. (**D**,**E**) Vertical sections of the ascomal wall. (**F**,**G**) Asci. (**H**) Ascal apices with an apical annulus. (**I**) Paraphyses. (**J**) Ascospores. (**K**,**L**,**U**,**V**) Conidiophores. (**M**–**Q**) Conidiophores with phialides, in detail (arrow indicates connectives through with the conidiophores anastomose). (**R**–**T**) Macroconidia. Images: (**A**–**T**) on the natural substrate; (**U**,**V**) on PCA after 4 months. Bars: (**A**–**C**) = 500 μm; (**D**,**L**) = 50 μm; (**E**) = 20 μm; (**F**–**K**,**M**–**T**) = 10 μm; (**U**,**V**) = 5 μm.

***Zanclospora aurea*** Réblová & Hern.-Restr., **sp. nov.** MycoBank MB837797. (Figure 10).

Typus: NEW ZEALAND, West Coast, Buller district, Victoria Forest Park, Rough Creek Road, ca. 4 km S of Inangahua, on decaying wood of a branch, 22 April 2005, M. Réblová M.R. 3515/NZ 808 (holotype PDD 118750, culture ex-type ICMP 23703 = CBS 147013).

Etymology: *Aureus* (L) golden, from *aurum* (gold), referring to the colour of golden-yellow colonies of the anamorph.

Description on the natural substrate: Colonies effuse to cushion-like, golden-yellow, consisting of *Zanclospora* conidiophores and ascomata. Teleomorph: Ascomata 220–320 μm diam, 250–300 μm high, superficial, aggregated, subglobose to conical, papillate, dark brown to black, glabrous, roughened. Ostiole periphysate. Ascomatal wall fragile, three-layered, 48–73 μm thick; outer layer (18–34.5 μm thick) composed of broadly polyhedral to globose, brown to golden-brown cells, a middle layer composed of dark brown, thick-walled, polyhedral cells, an inner layer composed of subhyaline to hyaline, thin-walled, elongated cells. Paraphyses 3–6 μm wide, tapering to 2–2.5 μm, hyaline, branching, septate. Asci 142–185(–192) × 16.5–20.5 μm (mean ± SD = 165.3 ± 16.8 × 18.5 ± 1.1 μm), cylindrical-fusiform, short-stipitate, apically broadly rounded to obtuse, with a non-amyloid apical annulus 4.5–5 μm wide, 2–2.5 μm high. Ascospores 28.5–35.5 × 7–8.5(–9) μm (mean ± SD = 31.9 ± 1.6 × 8.1 ± 0.5 μm), fusiform, 3-septate, not constricted at the septa, hyaline, finely verrucose, 2-seriate or obliquely 1-seriate within the ascus. Anamorph: Conidiophores 478–568 μm long, 4.5–6 μm wide above the bulbose base, 5–6.5 μm wide at the fertile region, erect, setiform, tapering gradually upwards, straight or gently bent, simple or branched, the main stalk often with one to several primary branches curved upwards, secondary and tertiary branches also develop, septate, light brown to golden-brown to dark yellow, thick-walled towards the base, paler and thinner-walled towards the apex, smooth, apex sterile, narrowly rounded. The upper part with several clavate, oval to irregularly shaped outgrowths, 3–4.5 × 5–6 μm, functioning as connecting elements through which the conidiophores anastomose to form a network. Conidiogenous cells monophialidic, 12.5–15.5(–17.5) × 4.5–6.5(–7) μm, tapering to 1–2 μm just below the collarette, discrete, lateral, arranged in groups of 2–5 in 1–3 whorls, ovoid to lageniform, light brown, smooth; collarettes flaring, 1.5–2.5 μm wide, 0.5–1 μm deep. Macroconidia 15–23(–24) × 3–4.5 μm (mean ± SD = 19.1 ± 2.0 × 3.6 ± 0.6 μm), falcate and strongly curved to almost horseshoe-shaped, narrowly rounded at both ends, aseptate, hyaline, smooth, accumulating in white to light yellow mass enveloping the whole fertile region. Synanamorph: Not observed.

Culture characteristics: On CMD colonies 6–7 mm diam, circular, slightly convex, margin entire, velvety, finely furrowed, brown to beige-brown occasionally with a dark brown outer zone of submerged growth, reverse dark brown. On MLA colonies 5–9 mm diam, circular to irregular, pulvinate, margin entire, velvety, furrowed, brown, reverse dark brown. On OA colonies 7–8 mm diam, circular, convex, margin entire, velvety, light grey, olivaceous grey at the margin, light olivaceous pigment diffusing into agar, reverse dark olivaceous-grey. On PCA colonies 5–7 mm diam, circular, slightly convex, margin entire to finely lobate, velvety, furrowed, beige-brown with a dark brown outer zone of submerged growth, reverse dark brown. Sporulation was sparse on MLA and PCA, absent on CMD and OA.

Description on PCA: Colonies effuse, vegetative hyphae subhyaline to light brown, septate, branched, becoming encrusted with age, 2–3.5 μm wide. Conidiophores 52–107 × 3–3.5 μm, semi-macronematous, erect, straight, unbranched, brown, sometimes reduced to single conidiogenous cells. Conidiogenous cells monophialidic, 12–15(–17) × 3–5 μm, tapering to 1.5(–2) μm just below the collarette, discrete, lateral or integrated, terminal, arise singly or in groups of 2–3 in a single whorl just below the septum, lageniform, brown; collarettes 2–2.5 × 1 μm. Macroconidia absent. Microconidia 5–6 × 1.5–2.5 μm (mean ± SD = 5.8 ± 0.5 × 2.0 ± 0.4 μm), clavate, truncate at the basal end, rounded at the apical end, aseptate, hyaline, smooth. Synanamorph: Not observed. Teleomorph: Not observed.

Habitat and geographical distribution: Saprobe on decaying wood, known from New Zealand.

Notes: *Zanclospora aurea* is well-distinguishable from other members of the genus in the golden-yellow colonies formed on the natural substrate, presence of connective elements on conidiophores, and falcate, strongly curved to almost horseshoe-shaped conidia. In vitro, colonies were very slow growing; on MLA they appeared pulvinate of somewhat crustose consistency, while on other media colonies were effuse with a spreading edge. The stanjehughesia-like synanamorph was not observed on the natural substrate or any of the growth media.

**Figure 11:**
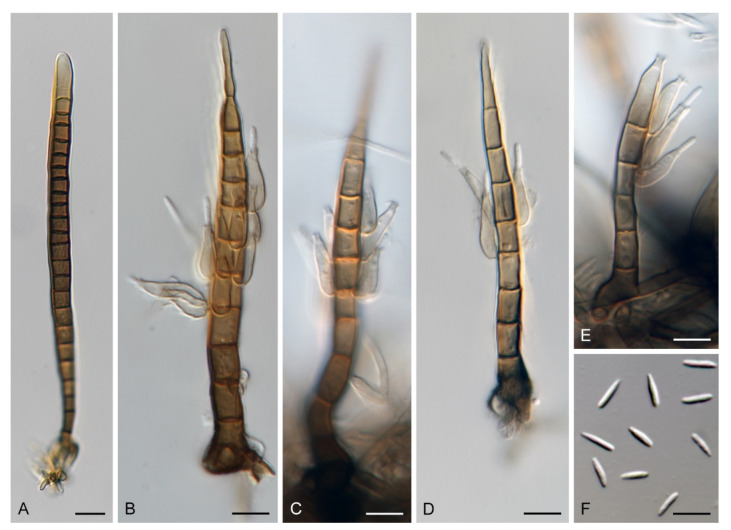
*Zanclospora clavulata* (CBS 146967 ex-type). (**A**) stanjehughesia-like conidiophores. (**B**–**E**) *Zanclospora* conidiophores. (**F**) Microconidia. Images: (**A**–**F**) on CMA with *U. dioica* stems after 4 weeks. Bars: (**A**) = 10 μm; (**B**–**F**) = 5 μm.

***Zanclosporaclavulata*** Réblová & Hern.-Rest., **sp. nov.** MycoBank MB837798. (Figure 11).

Typus: PORTUGAL, Minho Province, Lagoas do Bertiandos protected area, on dead wood of unidentified plant, November 2011, R.F. Castañeda-Ruíz, M. Hernández-Restrepo, J. Gené and J. Mariné-Gené (holotype CBS H-24519 as dried culture, culture ex-type CBS 146967 = FMR 12186).

Etymology: *Clavulate* (Latin) club-shaped, alternative form of *clavate* from *clava* (club), referring to the shape of microconidia.

Culture characteristics: On CMD colonies 15–17 mm diam, circular, slightly raised, margin entire to finely fimbriate, lanose, floccose, grey-brown with an outer dark olivaceous brown zone of submerged growth, reverse dark brown. On MLA colonies 24–27 mm diam, circular, slightly convex, margin entire, lanose, beige-grey with an outer dark grey-brown zone of submerged growth, reverse dark brown. On OA colonies 17–18 mm diam, circular, flat to slightly convex, margin entire to finely fimbriate, sparsely lanose, floccose, grey with an outer olivaceous grey zone of submerged growth, reverse dark olivaceous grey. On PCA colonies 19–20 mm diam, circular, flat to slightly raised, margin finely fimbriate, lanose, light grey-brown with an outer olivaceous brown zone of submerged, reverse brown-grey. Sporulation was moderate on CMA with *U. dioica* stems, absent on CMD, MLA, OA and PCA.

Description on CMA with *U. dioica* stems: Colonies effuse, vegetative hyphae subhyaline to brown, septate, branched, 1–2 μm wide. Anamorph: Conidiophores 33–68 μm long, 3–4 μm wide above the base, 3–4 μm wide at the fertile region, erect, setiform, unbranched, septate, cylindrical, gradually tapering upwards, brown to reddish-brown, smooth, apical cell sterile, subacute or developing into a phialide. Some conidiophores reduced, bearing one to several lateral phialides or reduced to single conidiogenous cells. Conidiogenous cells monophialidic, 7–10 × 2–3 μm, tapering to 0.7–1 μm below the collarette, discrete, lateral, appressed to the conidiophore, arranged in groups of 2–4 in 1–4 whorls, lageniform, pale brown to subhyaline, smooth, collarettes indistinct. Macroconidia absent. Microconidia 3.5–7 × 0.7–1 μm (mean ± SD = 4.0 ± 0.41 × 0.96 ± 0.1 μm), clavate to fusiform, tapering towards the basal end, rounded at the apical end, hyaline, smooth. Synanamorph: stanjehughesia-like. Conidiophores 135–155 μm long, 3–3.5 μm wide above the base, 6–7 μm wide at the midsection, erect, unbranched, septate, cylindrical, tapering towards the base, brown, apical cell rounded. Conidiogenous cells and conidia were not observed. Teleomorph: Not observed.

Habitat and geographical distribution: Saprobe on decaying wood, known from Portugal. Environmental data indicate another occurrence in the soil in Tasmania in the mixed forest biome with *Eucalyptus* sp. as a dominant plant species (Figure 6 and Figure 7) (Table S2).

Notes: *Zanclospora clavulata* is closely related to *Z. falcata*, *Z. iberica* and *Z. novae-zealandiae*. Initially, it was listed under *Z. iberica* [9], but the present four-gene phylogeny revealed it is a separate species (Figure 3). Its wildtype is unknown, so comparison with other species is somewhat limited. The herbarium material, which is no longer available, contained only the stanjehughesia-like synanamorph, but *Zanclospora* and the stanjehughesia-like synanamorphs were formed on CMA with *U. dioica* stems. In vitro, conidiophores of *Z. clavulata* produced only microconidia that are narrower (0.7–1 μm) than in other species (1–2.5 μm). When compared to other *Zanclospora* that exist in culture, it is the fastest-growing species.

**Figure 12:**
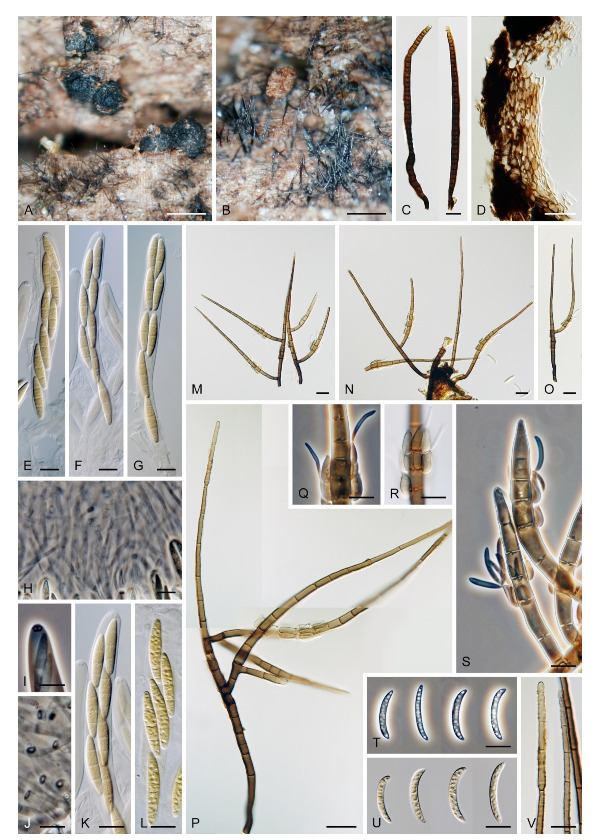
*Zanclospora falcata* (ICMP 23702 ex-type). (**A**) Ascomata associated with *Zanclospora* anamorph and stanjehughesia-like synanamorph. (**B**,**C**) Stanjehughesia-like conidiophores. (**D**) Vertical section of the ascomal wall. (**E**–**G**) Asci. (**H**) Paraphyses. (**I**) Ascal apex with an apical ring. (**J**) Empty asci with apical rings. (**K**,**L**) Ascospores. (**M**–**P**) Conidiophores. (**Q**–**S**) Conidiogenous cells. (**T**,**U**) Macroconidia. (**V**) Conidiophore apices covered with excrescences. Images: (**A**–**V**) on the natural substrate. Bars: (**A**,**B**) = 250 μm; (**C**,**D**,**M**–**P**) = 20 μm; (**E**–**L**,**Q**–**V**) = 10 μm.

**Figure 13:**
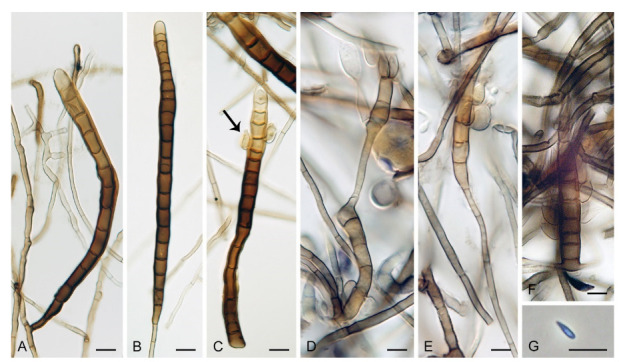
*Zanclospora falcata* (ICMP 23702 ex-type). (**A**–**C**) Stanjehughesia-like conidiophores (arrow indicates phialides). (**D**–**F**) Reduced *Zanclospora* conidiophores. (**G**) Microconidia. Images: (**A**–**G**) on MLA after 4–8 weeks. Bars: (**A**–**F**) = 10 μm; (**G**) = 5 μm.

***Zanclospora falcata*** Réblová & Hern.-Restr., **sp. nov.** MycoBank MB837799. (Figure 12 and Figure 13).

Typus: NEW ZEALAND, West Coast, Westland district, Mount Aspiring National Park, Haast, Roaring Billy Falls Walk, on decaying wood, 22 March 2005, M. Réblová M.R. 3297/NZ 567 (holotype PDD 118746, culture ex-type ICMP 23702 = CBS 147012).

Etymology: *Falcate* (Latin) from *falx* (sickle) meaning shaped like a sickle, and -*ate* (resembling), referring to the shape of conidia.

Description on the natural substrate: Colonies effuse, hairy, ochre-brown to brown, consisting of stanjehughesia-like and *Zanclospora* conidiophores and ascomata. Teleomorph: Ascomata 210–250 μm diam, 230–290 μm high, semi-immersed to superficial, solitary or in small groups, conical, papillate, dark brown to black, glabrous. Ostiole periphysate. Ascomatal wall fragile, two-layered, 31–41 μm thick; an outer layer composed of dark brown, thick-walled, polyhedral cells, an inner layer composed of subhyaline to hyaline, thin-walled, elongated, compressed cells. Paraphyses 3–5 μm wide, tapering to 2–2.5 μm, hyaline, branching, anastomosing, septate. Asci (104–)112–125(–132) × 11–13.5 μm (mean ± SD = 118.5 ± 7.5 × 12.3 ± 0.9 μm), cylindrical-clavate, short-stipitate, apically broadly rounded, with a non-amyloid apical annulus 3–3.5 μm wide, 1.5(–2) μm high. Ascospores 23.5–28.5(–30) × 4.5–5.5 μm (mean ± SD = 26.3 ± 1.2 × 4.8 ± 0.3 μm), fusiform, 3-septate, not constricted at the septa, hyaline, smooth, 2-seriate or obliquely 1-seriate within the ascus. Anamorph: Conidiophores 210–350 (–500) μm long, 5–7.5 μm wide above the base, 6–8 μm wide at the fertile region, erect, setiform, tapering gradually upwards, straight or gently bent, simple or branched, the main stalk often with several primary branches curved upwards at almost a right angle, secondary and tertiary branches also developed, septate, brown to reddish-brown and thick-walled towards the base, light brown to yellow-brown and thinner-walled towards the apex, the upper part of the main stalk and branches ornamented with numerous colourless excrescences, apex sterile, subacute. Conidiogenous cells monophialidic, 9–12.5 × 4–5.5 μm, tapering to 1–1.5 μm just below the collarette, discrete, lateral, arranged in groups of 2–5 in 1–3 whorls, ovoid to lageniform, subhyaline to light brown, smooth; collarettes 1.5–2.5 μm wide, 1–1.5 μm deep. Macroconidia 18–24.5 × 2.5–3 μm (mean ± SD = 21.2 ± 2.1 × 2.8 ± 0.2 μm), falcate, curved, hyaline, smooth, accumulating in white to yellow mass enveloping the whole fertile region. Synanamorph: stanjehughesia-like. Conidiophores 155–270 μm long, (4–)5–6.5 μm wide above the base, (9–)11–13 μm wide at the midsection, erect, unbranched, straight or bent, septate, cylindrical to cylindrical-fusiform, tapering towards the base, gradually tapering towards the apex, dark brown to reddish-brown, pale brown to yellow-brown towards the apex, rounded apically. Conidiogenous cells and conidia were not observed.

Culture characteristics: On CMD colonies 11–12 mm diam, circular, flat, margin entire to weakly fimbriate, velvety-lanose, dark grey-brown, reverse of the same colour. On MLA colonies 13–14 mm diam, circular, flat, margin fimbriate, velvety-lanose, aerial hyphae with numerous colourless exudates, dark grey-brown, reverse black. On OA colonies 18–20 mm diam, circular, flat, margin fimbriate to rhizoidal, slightly subsurface, sparsely lanose becoming cobwebby, hyphae towards the periphery decumbent, grey-brown to dark brown, reverse dark olivaceous grey to black. On PCA colonies 22–25 mm diam, circular, flat, slightly convex centrally, margin fimbriate, lanose, somewhat floccose, beige to light brown, aerial hyphae with numerous minute colourless exudates, reverse dark grey-brown to black. Sporulation was moderate on MLA and PCA, absent on CMD and OA.

Description on PCA: Colonies effuse, vegetative hyphae brown, septate, branched, 2–4 μm wide. Anamorph: Conidiophores 80−120 μm long, 5−6(−7.5) μm wide above the base, 7−8 μm wide at the fertile region, reduced and less complex than on the natural substrate, unbranched, light brown to reddish-brown, setiform, tapering towards the apex, smooth, sometimes apex develops into a phialide. Conidiogenous cells monophialidic, 8–9 × 3.5–4.5 μm, tapering to 1.5–2 μm just below the collarette, discrete, lateral, arranged in groups of 2–4 in 1–3 whorls, or terminal; collarettes 1.5–2 μm wide, 0.5–1 μm deep. Macroconidia absent. Microconidia 5−6 × 1(–1.5) μm (mean ± SD = 5.4 ± 0.5 × 1.1 ± 0.2 μm), clavate to oblong-clavate, hyaline, smooth. Synanamorph: stanjehughesia-like. Conidiophores as on the natural substrate, 210–310 μm long, 4.5–6.5 μm wide above the base, 8.5–11 μm wide at the midsection, occasionally with 1–2 lateral phialides. Conidia not observed. Teleomorph: Not observed.

Other material examined: NEW ZEALAND, West Coast, Buller district, Victoria Forest Park, Reefton, Big River Inanganua track; on decaying wood of *Nothofagus* sp., 6 March 2003, M. Réblová MR 2723/NZ 224B (PDD 119365).

Habitat and geographical distribution: Saprobe on decaying wood, known from New Zealand.

Notes: *Zanclospora falcata* can be confused with *Z. novae-zelandiae*, especially in characters of conidiophores, conidia and ascospores. Although both species share apical, setiform parts of conidiophores and branches ornamented with numerous colourless excrescences and falcate conidia, *Z. falcata* differs from *Z. novae-zelandiae* with shorter, more strongly curved conidia and shorter conidiophores of the stanjehughesia-like synanamorph. We observed that the excrescences may be absent on some conidiophores in a single collection of *Z. falcata*. Their teleomorphs are comparable, the asci of *Z. falcata* tend to be shorter, (104–)112–125(–132) × 11–13.5 μm vs (120–)126–139(–148) × 11–12(–13) μm, than the asci of *Z. novae-zelandiae*. For a full comparison of both species see notes of *Z. novae-zelandiae* and Table 2.

*Zanclospora falcata* is also similar to *Z. lateriphiala*, especially in anamorphic characters, and represents its counterpart in the Southern Hemisphere. Both species have a similar size of conidiophores and conidia but differ in conidiophore ornamentation, which is lacking in *Z. lateriphiala*. Besides, both species are well distinguishable in the size of asci and ascospores, which are smaller in *Z. lateriphiala*. In the four-gene analysis (Figure 3), *Z. falcata*, *Z. lateriphiala* and *Z. novae-zelandiae* were resolved as separate species.

**Figure 14:**
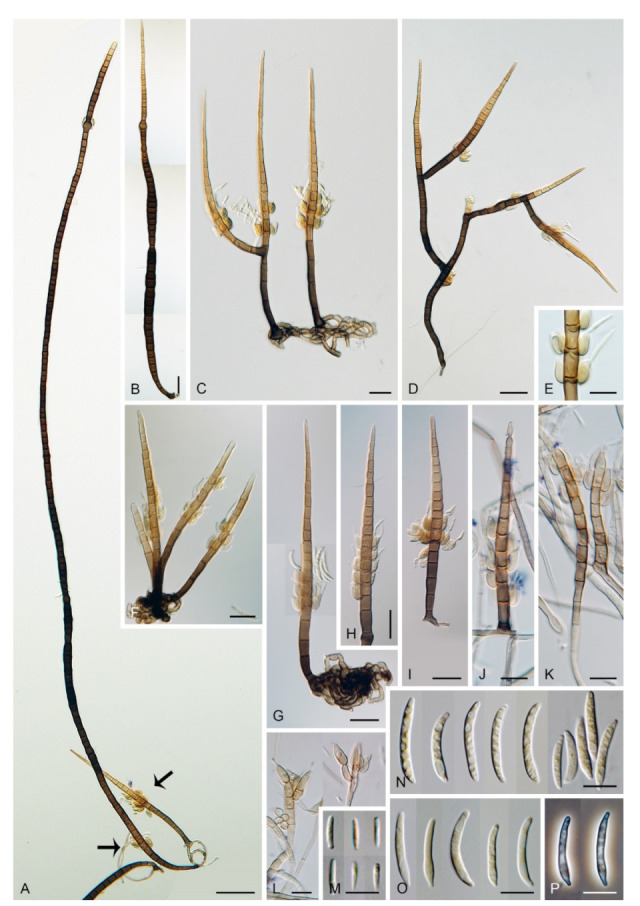
*Zanclospora iberica*. (**A**) Stanjehughesia-like and *Zanclospora* conidiophores (arrows indicate phialides). (**B**) Stanjehughesia-like conidiophore. (**C**,**D**,**F**–**I**) *Zanclospora* conidiophores. (**E**) Conidiogenous cells, in detail. (**J**–**L**) Reduced and less complex *Zanclospora* conidiophores. (**M**) Microconidia. (**N**–**P**) Macroconidia. Images: (**A**–**I**,**N**–**P**) on OA with *U. dioica* stems after 3 months; (**J**–**M**) on OA after 3 months; (**A**–**D**,**I**, **K**–**M**,**O**,**P**) from CBS 130426 (ex-type); (**E**–**H**,**J**,**N**) from CBS 130280. Bars: (**A**–**D**,**F**–**I**) = 20 μm; (**J**–**P**) = 10 μm.

***Zanclospora iberica*** Hern.-Restr., J. Mena & Gené, Stud. Mycol. 86: 85. 2017. (Figure 14).

Culture characteristics: On CMD colonies 10–12 mm diam, circular, slightly convex, margin fimbriate, velvety-lanose, cobwebby towards the margin, grey-brown centrally, dark brown at the margin, reverse dark brown. On MLA colonies 12–20 mm diam, circular, slightly convex, margin fimbriate or weakly undulate, lanose, floccose at the margin, grey-brown, dark brown at the margin, reverse dark brown to nearly black. On OA colonies 10–14 mm diam, circular, flat, margin fimbriate, lanose, floccose, cobwebby towards the margin, dark brown to olivaceous brown, reverse dark grey to nearly black. On PCA colonies 11–13 mm diam, circular, flat or slightly convex, margin fimbriate to rhizoidal, velvety-lanose, beige centrally, dark brown to russet towards the margin, with a dark brown outer zone of submerged growth, reverse dark brown-grey. Sporulation was abundant on OA with *U. dioica* stems, absent on CMD, MLA, OA and PCA.

Description on OA with *U. dioica* stems: Colonies effuse, hairy, vegetative hyphae semi-immersed, hyaline, subhyaline to brown, septate, branched, 1.5–3.5 μm wide. Anamorph: Conidiophores arising from mycelium on *Urtica* stems 128–318 μm long, 4.5–6 μm wide above the base, 6.5–9 μm wide at the fertile region, erect, setiform, tapering gradually upwards, straight or gently bent, branched or simple, the main stalk usually with one or several primary branches curved upwards, secondary and tertiary branches may also develop, septate, dark brown and thick-walled towards the base, light brown to yellow-brown and thinner-walled towards the apex, smooth, apical cell sterile, subacute. Conidiophores arising from mycelium on agar 52–96 μm long, 3.5–4.5 μm wide, 4–5 μm wide at the fertile region, less complex, unbranched, sometimes reduced to single conidiogenous cells. Conidiogenous cells monophialidic, 8–12.5 × 4.5–6.5 μm, tapering to 1–2.5 μm, discrete, lateral, sometimes percurrently elongating, arranged in groups of 2–5 in 1–3(–5) whorls, ovoid to lageniform, subhyaline to light brown, smooth; collarettes indistinct, 1.5–2 × 0.5(–1) μm. Macroconidia (12.5–)15.5–25 × 2–3 μm (mean ± SD = 20.2 ± 2.2 × 2.4± 0.3 μm), falcate, slightly obtuse at the basal end, narrowly rounded at the apical end, aseptate, sometimes inflated near the base, hyaline, smooth. Microconidia (5–)6–10.5 × 1.5–2 μm (mean ± SD = 8.2 ± 1.6 × 1.6 ± 0.2 μm), clavate to oblong-clavate, straight or gently curved, aseptate, hyaline, smooth. Synanamorph: stanjehughesia-like. Conidiophores 340–660(–920) μm long, (3.5–)4.5–6 μm wide above the base and 9–14 μm wide at the midsection, erect, sinuous, septate, sometimes inflated, sterile, rarely with 1–2 lageniform phialides near the base, tapering towards the base, gradually tapering towards the tip, dark brown, light brown to yellow-brown towards the apex, apical cell bluntly or narrowly rounded. Conidia not observed. Teleomorph: Not observed.

Material examined: SPAIN, Asturias, Picos de Europa National Park, La Molina, on dead wood of unidentified plant, July 2010, M. Hernández-Restrepo, J. Mena-Portales & J. Guarro (holotype CBS H-22995, culture ex-type CBS 130426 = FMR 11584). SPAIN, Galicia, Los Ancares Natural reserve, plant debris, 12 Aug. 2010, M. Hernández-Restrepo, J. Mena-Portales & J. Guarro (culture CBS 130280 = FMR 11022 = IMI 500756).

Habitat and geographical distribution: The species is a saprobe on decayed plant material and is so far known only from Spain ([9], this study).

Notes: For additional description and illustrations refer to Hernández-Restrepo et al. [9]. Although conidiophores similar to *Stanjehughesia* were the only anamorphic structure observed on the natural substrate, *Zanclospora* and stanjehughesia-like (Figure 14A,B) conidiophores were observed on *Urtica* stems in vitro. The *Zanclospora* conidiophores matched well the protologue but varied in size. Those which developed from mycelium on agar were smaller and less complex (Figure 14J–L) than those formed directly on *Urtica* stems (Figure 14C–I). The smaller conidiophores and single conidiogenous cells produced only microconidia.

**Figure 15:**
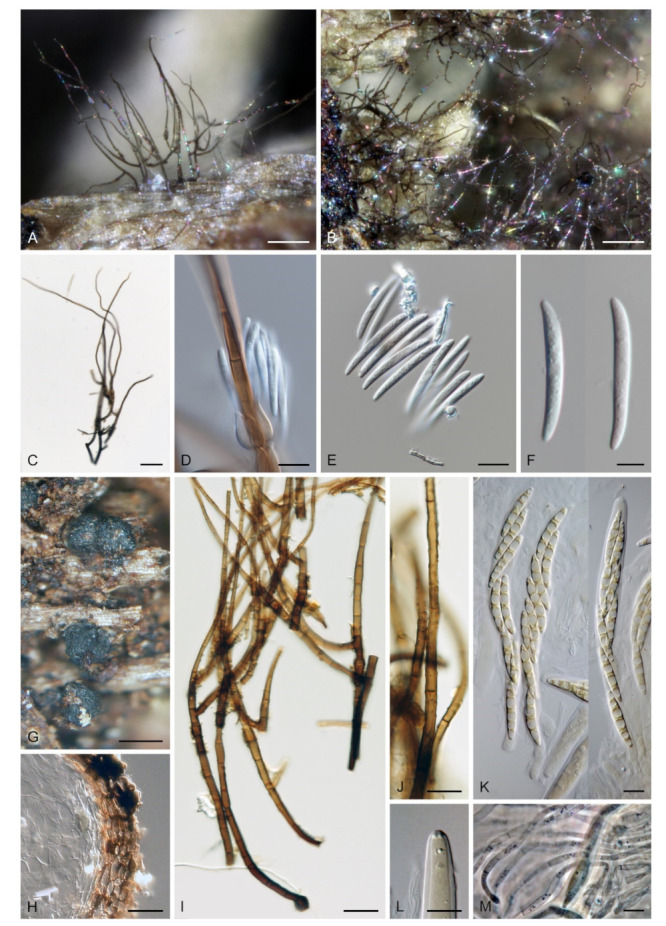
*Zanclospora novae-zelandiae*. (**A**–**C**,**I**) Conidiophores. (**D**) Phialides with conidia. (**E**,**F**) Conidia. (**G**) Ascomata. (**H**) Vertical section of the ascomal wall. (**J**) Anastomosing conidiophores with excrescences, in detail. (**K**) Asci. (**L**) Ascal apex. (**M**) Paraphyses. Images: (**A**–**M**) on the natural substrate; (**A**–**F**) from PDD 20737 (holotype); (**G**–**M**) from ICMP 15112. Bars: (**A**,**B**) = 100 μm; (**C**) = 50 μm; (**D**,**E**,**J**–**M**) = 10 μm; (**F**) = 5 μm; (**G**) = 250 μm; (**H**) = 20 μm; (**I**) = 25 μm.

**Figure 16:**
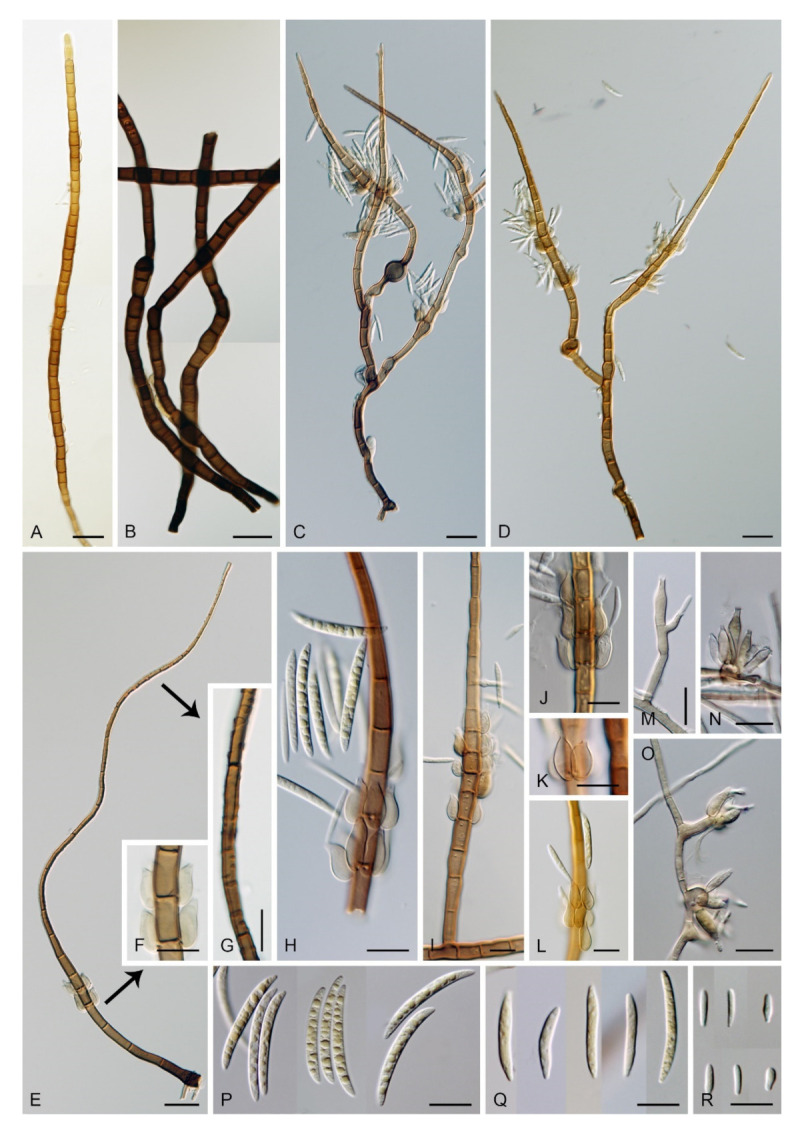
*Zanclospora novae-zelandiae*. (**A**,**B**) Stanjehughesia-like conidiophores. (**C**–**E**) *Zanclospora* conidiophores. (**F**,**H**–**L**) Conidiophores and branches with phialides in whorls. (**G**) Apical, setiform part of the conidiophore with excrescences, in detail. (**M**–**O**) Single phialides or less complex conidiophores with phialides in verticillate arrangement. (**P**,**Q**) Macroconidia. (**R**) Microconidia. Images: (**A**,**C**,**D**,**I**–**L**,**Q**) on CMA with *U. dioica* stems after 3 months; (**B**,**E**–**H**,**P**) on the natural substrate; (**M**–**O**,**R**) on MLA after 6 weeks; (**A**,**C**,**D**,**I**–**L**,**M**–**O**,**Q**,**R**) from ICMP 15781 (ex-epitype); (**B**,**E**–**H**,**P**) from PDD 80663. Bars: (**A**–**E**) = 20 μm; (**F**–**R**) = 10 μm.

*Zanclospora iberica* appears to be most similar to *Z. novae-zelandiae*. Because the wildtype of *Z. iberica* is unknown, both species can only be compared in culture. The sterile apical part of the conidiophore stalk and branches is smooth in both species. However, it is ornamented with disk-like excrescences in *Z. novae-zelandiae* on the natural substrate (Figure 15J and Figure 16E,F). In the absence of this diagnostic character, the species are practically indistinguishable when grown in culture. The size of their macroconidia overlaps significantly, i.e., (12.5–)15.5–25 × 2–3 μm (this study) and 12.5–22 × 2–3 μm *fide* Hernández-Restrepo et al. [9] in *Z. iberica* vs 16–24 × (1.5–)2–3 μm in *Z. novae-zelandiae* (ICMP 15781 ex-epitype strain). On the natural substrate, the macroconidia of *Z. novae-zelandiae* tend to be longer, 24–28.5 × (2–)2.5–3 μm in the epitype (PDD 80663), 23.5–39 × 2.5–3.5 μm in the holotype (PDD 20727). Thus, we expect that also macroconidia of *Z. iberica* may be longer in natural conditions. In comparison to *Z. novae-zelandiae*, the conidiophores of *Z. iberica* appeared to be less flexuous, and conidia were usually slightly inflated towards the basal end.

***Zanclospora jonesii*** (R.H. Perera, Maharachch. & K.D. Hyde) Réblová, A.N. Mill. & Hern.-Restr., **comb. nov.** MycoBank MB837800.

Basionym: *Chaetosphaeria jonesii* R.H. Perera, Maharachch. & K.D. Hyde, Mycosphere 7: 1311. 2016.

Habitat and geographical distribution: The species is a saprobe on decorticated wood, known so far from Asia in Thailand [86]. Environmental data confirm another occurrence in the soil in Guangdong province, southeastern China, in the forest biome with *Schima superba* and *Michelia macclurei* as the dominant plant species (Figure 5 and Figure 7) (Table S2).

Notes: For description and illustrations, see Perera et al. [86]. The *Zanclospora* conidiophores were not observed, the stanjehughesia-like conidiophores, probably mistaken for setae, occurred on ascomata and the nature substrate around them (see Discussion). In the four-gene phylogenetic tree (Figure 3), *Z. jonesii* clustered as a sister to *Z. tropicalis*. Both species share cylindrical ascospores bent near the basal end, but differ in their size; the ascospores of *Z. jonesii* are shorter and narrower, 16.2–17.7 × 2.8–3.6 μm *fide* Perera et al. [86] than those of *Z. tropicalis*, 19–26 × 3.2–6.3 μm *fide* Fernández and Huhndorf [12]. 

***Zanclospora lateriphiala*** (F.A. Fernández & Huhndorf) Réblová, A.N. Mill. & Hern.-Restr., **comb. nov.** MycoBank MB837801.

Basionym: *Chaetosphaeria lateriphiala* F.A. Fernández & Huhndorf, Fung. Divers. 18: 24. 2005.

Culture characteristics: On CMD colonies 11–14 mm diam, circular, raised, margin fimbriate, lanose, cobwebby at the margin, colony centre beige, dark brown at the margin, later with a distinct dark brown outer zone of submerged growth, reverse dark brown to nearly black. On MLA colonies 15–17 mm diam, circular, convex, margin fimbriate, lanose, floccose, aerial mycelium with numerous minute colourless exudates, beige, brown at the margin, reverse dark brown. On OA colonies 8–9 mm diam, circular, flat, margin fimbriate, cobwebby becoming mucoid, smooth towards the periphery, mainly comprising of submerged mycelium, olivaceous black, reverse black. On PCA colonies 10–11 mm diam, circular, flat, margin fimbriate to rhizoidal, lanose, floccose, beige-brown centrally, with a prominent dark brown to russet outer zone of the submerged growth, reverse dark brown. Sporulation was absent on all media.

Material examined: USA, Wisconsin, Green Co., New Glarus State Park, 13 Sep. 1995, on decaying wood (20 cm log), S.M. Huhndorf S.M.H. 1546-1 (culture CBS 147014). USA, Illinois, Forman Cypress Swamp, NW of Belknap, Johnson County, Illinois, on submerged decayed wood, 9 April 1969, J.L. Crane 80-69 (ILLS43049). USA, Illinois, Jackson Hollow, Pope County, on decayed wood, 9 Apr. 1969, J.L. Crane 25-69 (ILLS35030). *Ibid.*, J. L. Crane 27-69 (ILLS34732).

Habitat and geographical distribution: Saprobe on decorticated wood, known from North America in the USA (Illinois, Indiana, North Carolina, Wisconsin) [12].

Notes: For description and illustrations, see Fernández and Huhndorf [12]. According to these authors, repeated transfers of mycelium in vitro resulted in the loss of the typical *Zanclospora* conidiophores; they were reduced to single conidiogenous cells or whorls of cells on hyphae reminiscent of *Phaeostalagmus* and produced smaller, clavate conidia. It is in agreement with our observations in other *Zanclospora*, conidiophores formed on agar are generally less complex and produce only microconidia. *Zanclospora lateriphiala* is similar to *Z. falcata* but differs in the smooth setiform part of conidiophores and branches and smaller asci and ascospores. The stanjehughesia-like synanamorph has not been yet reported for *Z. lateriphiala*. For a full comparison with other species, see Table 2.

Three collections of *Z. novae-zelandiae* on decaying wood (ILLS34732, ILLS35030, ILLS43049), reported by Schoknecht and Crane [21] from the USA, have been revised. Based on the published description, the conidia (15.5–23 × 2.3–3.3 μm) and overall characteristics of conidiophores and phialides matched those of *Z. falcata* and *Z. lateriphiala*. However, the ornamentation of conidiophores was not given. Examination of these collections revealed that the upper setiform parts of conidiophores and branches are smooth and the conidiophores are more robust unlike those of *Z. falcata*. These collections represent *Z. lateriphiala*, which is common in this region (Figure 7).

***Zanclospora novae-zelandiae*** S. Hughes & W.B. Kendr., N. Z. J. Bot. 3: 152. 1965. (Figure 15 and Figure 16).

Typification: NEW ZEALAND, Westland, Lake Ianthe, Pukekura, on decaying bark and wood of *Weinmannia racemosa*, 8 April 1963, S. Hughes (holotype PDD 20737). NEW ZEALAND, North Canterbury, Selwyn district, Arthur’s Pass National Park, on decaying wood and bark of *Nothofagus solandri* var. *cliffortioides* × *fusca*, 29 September 2004, J.A. Cooper JAC9132 (epitype MBT 394463 designated here, PDD 118975 as dried culture on CMA with *U. dioica* stems) (PDD 80663 voucher, culture ex-epitype ICMP 15781).

Description on the natural substrate: Colonies effuse, hairy, golden-brown to ochre-brown to light brown, consisting of stanjehughesia-like and *Zanclospora* conidiophores and ascomata. Teleomorph: Ascomata 230–270 μm diam, 250–300 μm high, superficial, basally immersed, solitary or in small groups, conical, papillate, dark brown to black, glabrous. Ostiole periphysate. Ascomatal wall fragile, two-layered, 28–35 μm thick; outer layer composed of dark brown, thick-walled, polyhedral cells, an inner layer composed of subhyaline to hyaline, thin-walled, elongated, compressed cells. Paraphyses 3–4.5 μm wide, tapering to 1.5–2.5 μm, hyaline, branching, anastomosing, septate. Asci (120–)126–139(–148) × 11–12(–13) μm (mean ± SD = 132.9 ± 4.6 × 11.9 ± 0.6 μm), cylindrical-clavate, short-stipitate, apically broadly rounded to obtuse, with a non-amyloid apical annulus 3–3.5 μm wide, ca. 2 μm high. Ascospores 25–29.5(–31) × 4–5 μm (mean ± SD = 27.9 ± 1.5 × 4.6 ± 0.3 μm), fusiform, 3-septate, not constricted at the septa, hyaline, smooth, 2-seriate or obliquely 1-seriate within the ascus. Anamorph: Conidiophores 360–450 μm long, 5.5–7 μm wide above the base, 5.5–6.5 μm wide at the fertile region, erect, setiform, tapering gradually upwards, straight or bent, simple or sparsely branched, the main stalk with primary branches curved upwards at the almost right angle, secondary and tertiary branches develop, septate, dark-brown to reddish-brown and thick-walled towards the base, light brown to yellow-brown and thinner-walled towards the apex, the upper part of the main stalk and branches with colourless excrescences, apex subacute, sterile. Conidiogenous cells monophialidic, 10–13 × (3.5–)4–4.5 μm, tapering to 1–1.5 μm just below the collarette, discrete, lateral, arranged in groups of 2–5 in 1–3 whorls, ovoid to lageniform, subhyaline to light brown, smooth; collarettes usually indistinct, ca. 1.5 μm wide, ca. 1 μm deep. Macroconidia 24–28.5 × (2–)2.5–3 μm (mean ± SD = 26.4 ± 1.4 × 2.5 ± 0.2 μm), falcate to somewhat cymbiform, gently curved, aseptate, hyaline, smooth, filled with numerous drops, accumulating in a whitish to yellow mass enveloping the whole fertile region. Synanamorph: stanjehughesia-like. Conidiophores 364–675 μm long, 4.5–5 μm wide above the base, 8–10.5 μm wide at the midsection, sometimes inflated in the middle, erect, unbranched, straight or slightly sinuous, septate, cylindrical to cylindrical-fusiform, tapering towards the base, gradually tapering towards the apex, dark brown to reddish-brown, darkest near the base, apical cell light brown to yellow-brown, rounded. Conidiogenous cells and conidia were not observed.

Culture characteristics: On CMD colonies 11–12 mm diam, circular to irregular, flat, margin fimbriate, velvety-lanose at the centre becoming cobwebby towards the margin, colony centre olivaceous brown, olivaceous beige at the margin, reverse dark brown. On MLA colonies 11–12 mm diam, circular, slightly convex, margin fimbriate, velvety, cobwebby at the margin, colony centre beige, dark brown at the margin, reverse dark brown. On OA colonies 6–7 mm diam, circular to irregular, flat, margin fimbriate to rhizoidal, cobwebby centrally, smooth towards the periphery, dark olivaceous brown, light brown pigment diffusing into the agar, reverse brown. On PCA colonies 5–7 mm diam, circular to irregular, flat, margin fimbriate, velvety, sometimes mucoid at the centre, beige-brown, with a dark brown outer zone of submerged growth, reverse dark brown. Sporulation was absent on CMD and OA, moderate on PCA and MLA, abundant on CMA with *Urtica* stems.

Description on CMA with *U. dioica* stems: Colonies effuse, vegetative hyphae subhyaline to light brown, smooth, semi-immersed, branched, septate, 1.5–3 μm wide. Anamorph: Conidiophores arising from *Urtica* stems 277–380 μm long, 4.5–5.5(–6) μm wide above the base, 6.5–8 μm wide at the fertile region, septate, branched, smooth, apex subacute. Conidiophores arising from mycelium on agar 20–45 × 2–2.5 μm, less complex, subhyaline to light brown, simple or branched, septate, occasionally reduced to single conidiogenous cells or a whorl of several phialides. Conidiogenous cells monophialidic, (8.5–)9–13(–14.5) × 2.5–3.5 μm, tapering to 1.5–2 μm, discrete, lateral, arranged in groups of 2–5 in 1–4(–5) whorls; collarettes 1.5–2 μ wide, ca. 0.5 μm deep. Macroconidia 16–24 × (1.5–)2–3 μm (mean ± SD = 19.4 ± 2.0 × 2.4 ± 0.3 μm), falcate, straight or gently curved, slightly truncate at the basal end, tapering towards the apical end, aseptate, hyaline, smooth. Microconidia 5–8 × 1.5–2 μm (mean ± SD = 6.9 ± 1.2 × 1.7 ± 0.2 μm), clavate to oblong-clavate, straight or gently curved, aseptate, hyaline, smooth. Synanamorph: stanjehughesia-like. Conidiophores as on the natural substrate, 605–800 μm long, 5–5.5 μm wide above the base, 6.5–7.5 μm wide at the midsection. Conidiogenous cells and conidia were not observed. Teleomorph: Not observed.

Other material examined: NEW ZEALAND, West Coast, Buller district, Victoria Forest Park, Black Points ca. 1.5 km SE of Reefton, Murray Creek track, on decaying wood and the inner side of the bark of *Nothofagus* sp., 21 February 2003, M. Réblová and K.A. Seifert M.R. 2589/NZ 54 (PDD 119105, culture ICMP 15112).

Habitat and geographical distribution: *Zanclospora novae-zelandiae* is a saprobe on decaying wood and bark of *Libocedrus bidwi*, *Nothofagus fusca*, *N. solandri* var. *cliffortioides × fusca*, *N. truncata*, *Oenocarpus* sp., *Weinmannia racemosa* and other unidentified hosts. It is known from Brazil, Canada, India, Japan, New Zealand, Taiwan, USA and Vietnam ([1,8,20,22,23,88,89,90], this study).

Notes: Examination of the holotype of *Z. novae-zelandiae* and three collections tentatively identified as this species originating from New Zealand (PDD 80663, PDD 118746, PDD 119105), and comparison of their DNA sequences, revealed two separate species lineages. It confirmed our suspicion that it may contain cryptic species, based on different morphological profiles of *Z. novae-zelandiae* in the literature. Although two collections, PDD 80663 and PDD 118746, fit into the protologue of *Z. novae-zelandiae* [1], they differed mainly in the size of conidia that did not overlap and the size of the associated stanjehughesia-like conidiophores. Hughes and Kendrick [1] introduced *Z. novae-zelandiae* with conidiophores 155–550(–750) μm long and conidia 18–35 × 1.6–2.6 μm, citing a relatively wide range of conidial lengths. The examination of the holotype of *Z. novae-zelandiae* PDD 20737 revealed a fungus with conidia 23.5–39 × 2.5–3.5 μm (mean = 29.1 × 3.1 μm), conidiophores 350–520 μm long, the stanjehughesia-like conidiophores were not observed. The specimen PDD 80663 had conidia 24–28.5 × (2–)2.5–3 μm, the stanjehughesia-like synanamorph was present with conidiophores 364–675 μm long. The other specimen PDD 118746 had shorter conidia 18–24.5 × 2.5–3 μm, yet within the range given in the original description, and shorter stanjehughesia-like conidiophores 155–270 μm long. Both collections are otherwise highly similar and their conidiophores have distinct colourless excrescences on the apical setiform part. In the four-gene analysis, they were shown as separate, though closely related lineages (Figure 3). Given their distinct conidial and synanamorph morphology accompanied by molecular data, they are treated as sibling species. The specimen PDD 80663 (culture ICMP 15781) is used to interpret the holotype of *Z. novae-zelandiae*. Unfortunately, its herbarium material is largely depauperate. Therefore, a dried culture on CMA with *Urtica* stems (Figure 16) is selected to serve as the epitype. The specimen PDD 118746 is introduced as a new species, *Z. falcata*. The third collection PDD 119105 is *Z. novae-zelandiae*; it contained mature ascomata associated with a somewhat aged colony of *Zanclospora* conidiophores with mostly disintegrated phialides and absent conidia (Figure 15G–M). The stanjehughesia-like conidiophores were not observed. The teleomorph of *Z. novae-zelandiae* is reported for the first time.

It is challenging to distinguish *Z. novae-zelandiae* from *Z. iberica*. In culture, when grown on *Urtica* stems, both species are similar in characters of conidia, phialides and smooth conidiophores. For comparison of both species and discussion, see notes to *Z. iberica*.

In addition to New Zealand, *Z. novae-zelandiae* was also reported from other geographical areas (Figure 7). However, all of these collections lacked the ornamentation of the conidiophore wall, or this character was not mentioned in the description [8,20,21,22,23]. Besides, the size of conidia varied among these collections and corresponded to the long-spored *Z. novae-zelandiae* s. str., the newly segregated short-spored *Z. falcata*, both from New Zealand, but also to *Z. lateriphiala* from North America. Examination of three collections identified as *Z. novae-zelandiae* by Schoknecht and Crane [21] from the USA with smooth conidiophores and conidia 15.5–23 × 2.3–3.3 μm revealed they represent *Z. lateriphiala*. The specimen of *Z. novae-zelandiae* recorded from Brazil by Almeida et al. [8] has conidia significantly shorter (10–16.5 × 1–2 μm) than *Z. falcata*, *Z. lateriphiala* and *Z. novae-zelandiae*, and likely represents another cryptic species in the *Z. novae-zelandiae* species complex. Interestingly, Mel’nik et al. [23] recorded a specimen of *Z. novae-zelandiae* from Vietnam with exclusively unbranched, smooth conidiophores and conidia 22–24(–26) × 2–2.4 μm. Although we are aware of inconsistencies in the published phenotypes of *Z. novae-zelandiae*, these records are listed above but need to be verified. We present the first molecular data of *Z. novae-zelandiae*; however, more concentrated sampling is required to assess its global geographical distribution. We should also consider the possibility that the species is endemic to New Zealand.

***Zanclosporaphaeostalacta*** (Réblová) Réblová, A.N. Mill. & Hern.-Rest., **comb. nov.** MycoBank MB837802.

Basionym: *Chaetosphaeria phaeostalacta* Réblová, Stud. Mycol. 50: 183. 2004.

Culture characteristics: On CMD colonies 8–10 mm diam, circular, slightly convex, margin weakly fimbriate, velvety, dark beige-brown, reverse dark brown. On MLA colonies 7–8 mm diam, circular, slightly convex, margin entire, velvety-lanose, dark beige-brown, darker at the margin, reverse dark brown. On OA colonies 4–5 mm diam, circular, flat, margin entire to weakly fimbriate, lanose, olivaceous beige, reverse dark brown. On PCA colonies 4–5 mm diam, circular, convex, margin entire to weakly fimbriate, lanose, beige-brown with a dark brown outer zone of submerged growth, reverse dark brown. Sporulation absent on all media; moderate on PCA after prolonged incubation.

Material examined: NEW ZEALAND, West Coast, Westland district, Ross, Totara River valley, Totara forest, on decorticated wood of a branch, 7 March 2003, M. Réblová MR 2735/NZ 237 (holotype PDD 78274, culture ex-type ICMP 15137 = CBS 114554).

Habitat and geographical distribution: Saprobe on decaying wood, known from New Zealand [85].

Notes: For description and illustrations, refer to Réblová [85]. The *Zanclospora* or stanjehughesia-like conidiophores have not been observed in this species; only an anamorph of the phaeostalagmus-like morphotype was formed when grown in culture. It is characterised by pigmented, macronematous to semi-macronematous conidiophores with phialides arranged laterally, singly or in verticilli, or terminal on short branches producing ellipsoidal, slightly apiculate microconidia. Among other *Zanclospora*, *Z. phaeostalacta* possesses one of the largest (28–)30–38(–40) × 5–6(–8) μm, 5–7-septate ascospores, while other members of the genus have ascospores with a maximum of five septa and usually up to 31 μm long, except for *Z. aurea* with ascospores 28.5–35.5 μm long.

**Figure 17:**
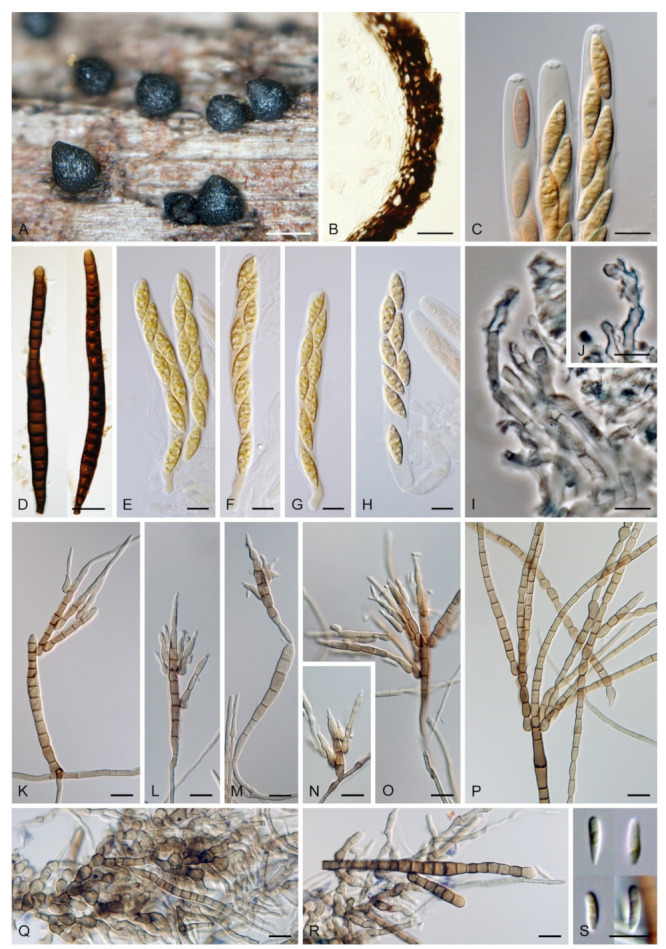
*Zanclospora ramifera* (ICMP 22738 ex-type). (**A**) Ascomata. (**B**) Vertical section of the ascomal wall. (**C**) Ascal apices with apical rings. (**D**) Stanjehughesia-like conidiophores. (**E**–**H**) Asci with ascospores. (**I**) Paraphyses. (**J**) Ascogenous hyphae. (**K**–**P**) *Zanclospora* conidiophores. (**Q**,**R**) Mycelium with stanjehughesia-like conidiophores. (**S**) Microconidia. Images: (**A**–**J**) on natural substrate; (**K**–**S**) on MLA after 8 weeks. Bars: (**A**) = 250 μm; (**B**,**D**) = 20 μm; (**C**,**E**–**R**) = 10 μm; (**S**) = 5 μm.

***Zanclosporaramifera*** Réblová & Hern.-Rest., **sp. nov.** MycoBank MB837803. (Figure 17).

Typus: NEW ZEALAND, West Coast, Westland district, Westland Tai Poutini National park, Lake Matheson, on decaying wood associated with *Lentomitella magna*, 13 April 2005, M. Réblová M.R. 2961/NZ 781B (holotype PDD 118747, culture ex-type ICMP 22738 = CBS 147101).

Etymology: *Ramus* (L) branch, *fero* (L) carry or bear, referring to the branched conidiophores.

Description on the natural substrate: Colonies effuse, hairy, dark brown, composed of ascomata and the stanjehughesia-like conidiophores. Teleomorph: Ascomata 220–250 μm diam, 260–300 μm high, superficial, solitary or in small groups, conical, papillate, dark brown to black, glabrous. Ostiole periphysate. Ascomatal wall fragile, two-layered, 25–30 μm thick; outer layer composed of dark brown, thick-walled, polyhedral cells, an inner layer composed of subhyaline to hyaline, thin-walled, elongated, compressed cells. Paraphyses 3–5.5 μm wide, tapering to ca. 2.5 μm, hyaline, branching, anastomosing, septate. Asci 98–125 × (10.5–)11–12.5 μm (mean ± SD = 109.0 ± 6.7 × 12.0 ± 0.5 μm), 8-spored, cylindrical-clavate, short-stipitate, apically broadly rounded to obtuse, with a non-amyloid apical annulus 3.5–4.5 μm wide, 1.5–2 μm high. Ascospores 17–24(–25.5) × 5.5–7 μm (mean ± SD = 20.5 ± 2.3 × 6.3 ± 0.4 μm), fusiform, 3-septate, not constricted at the septa, hyaline, smooth, 2-seriate or partly obliquely 1-seriate within the ascus. Anamorph: Not observed. Synanamorph: stanjehughesia-like. Conidiophores 155–195 μm long, 4–5 μm wide above the base, 10.5–12(–15) μm wide at the midsection, erect, growing sparsely near the ascomata, unbranched, slightly sinuous, septate, cylindrical to cylindrical-fusiform, tapering towards the base, dark reddish-brown, apical cell paler, rounded. Conidiogenous cells and conidia were not observed.

Culture characteristics: On CMD colonies 12–13 mm diam, circular, convex, margin entire to weakly fimbriate, lanose, somewhat floccose, beige at the centre, dark beige to brown towards the margin, reverse dark brown. On MLA colonies 15–16 mm diam, circular, slightly convex centrally, margin weakly fimbriate, lanose, cobwebby towards the periphery, zonate, beige at the centre, with dark brown to dark reddish-brown middle zone and paler brown outer zone, reverse brown. On OA colonies 6–8 mm diam, circular, flat, margin entire, smooth to cobwebby, dark olivaceous grey with an outer zone of a similar colour of submerged growth, reverse dark brown. On PCA colonies 5–6 mm diam, circular, flat, margin weakly fimbriate, cobwebby, brown with a dark brown outer zone of submerged growth, reverse dark brown. Sporulation was abundant on MLA, absent on CMD, OA and PCA.

Description on MLA: Colonies effuse, vegetative hyphae subhyaline to light brown, septate, branched, 2–3.5 μm wide. Anamorph: Conidiophores (30–)55–235 μm long, 2–3.5 μm wide above the base, 3.5–6 μm wide at the fertile region, sometimes reduced to single conidiogenous cells, cylindrical-fusiform, erect, simple or branched, with several primary branches, secondary and tertiary branches often develop, sometimes longer than the main stalk, septate, straight or slightly bent, light brown to light reddish-brown, smooth, apical cell developed into a phialide or sterile, apex rounded, smooth; the fertile region is situated in the upper or middle part of the conidiophore. Conidiogenous cells monophialidic, 7–12.5 × 2.5–4.5μm, tapering to 1–1.5 μm, discrete, lateral, arise just below the septa, appressed to the conidiophore, arranged singly or in groups of 2–3 in 1–3 whorls, lageniform, in older cultures percurrently elongating, light brown to subhyaline, smooth; collarettes indistinct. Macroconidia absent. Microconidia 5–6 × 1.5 μm (mean ± SD = 5.5 ± 0.5 × 1.5 ± 0.2 μm), clavate to oblong-clavate, straight or gently curved, tapering towards the basal end, rounded at the apical end, hyaline, smooth. Synanamorph: stanjehughesia-like. Conidiophores as on the natural substrate, 53–154 μm long, 3–3.5 μm wide above the base and 3.5–7.5 μm wide at the midsection, occasionally branched. Conidiogenous cells and conidia were not observed. Teleomorph: Not observed.

Other material examined: NEW ZEALAND, West Coast, Grey district, Victoria Forest Park, Lake Christabel track, Palmer’s Hut ca. 18 km SW of Springs Junction on an unpaved road, on decaying wood of *Nothofagus* sp., 1 March 2003, M. Réblová M.R. 2680/NZ 176 (culture ICMP 15127).

Habitat and geographical distribution: Saprobe on decaying wood of *Nothofagus* sp. and another unidentified host, known from New Zealand.

Notes: The *Zanclospora* conidiophores were formed only in culture, though less complex, and produced only microconidia. *Zanclospora lateriphiala* closely resembles *Z. ramifera*, but differs in slightly shorter (95–113 μm) asci, narrower (4.5–6 μm) ascospores and characters of the *Zanclospora* anamorph in vitro [12]. When grown in culture, the conidiophores of *Z. lateriphiala* are unbranched and produce fusiform to oblong-clavate macroconidia (11–18 × 4–4.5 μm), unlike the branched conidiophores with microconidia of *Z. ramifera*.

***Zanclosporasylvatica*** (F.A. Fernández & Huhndorf) Réblová, A.N. Mill. & Hern.-Rest., **comb. nov.** MycoBank MB837804.

Basionym: *Chaetosphaeria sylvatica* F.A. Fernández & Huhndorf, Fung. Diver. 18: 38. 2005.

Habitat and geographical distribution: Saprobe on decaying wood, known from the Caribbean (Puerto Rico) [12].

Notes: For description and illustrations, refer to Fernández and Huhndorf [12]. In the four-gene phylogeny, *Z. sylvatica* was inferred as a sister to *Z. jonesii* and *Z. tropicalis*. *Zanclospora sylvatica* forms only the phaeostalagmus-like synanamorph *in vitro*, the stanjehughesia-like and *Zanclospora* conidiophores were not observed. The species is characterised by symmetrical, 3-septate, fusiform ascospores in contrast to the asymmetrical ascospores of *Z. jonesii* and *Z. tropicalis*.

***Zanclospora tropicalis*** (F.A. Fernández & Huhndorf) Réblová, A.N. Mill. & Hern.-Rest., **comb. nov.** MycoBank MB837805.

Basionym: *Chaetosphaeria tropicalis* F.A. Fernández & Huhndorf, Fungal Diversity 18: 40. 2005.

Habitat and geographical distribution: Saprobe on decaying wood, known only from the Caribbean (Puerto Rico) and Central America (Costa Rica) [12].

Notes: For description and illustrations, refer to Fernández and Huhndorf [12]. A synanamorph similar to *Phaeostalagmus* has been observed in culture [12], but stanjehughesia-like and *Zanclospora* conidiophores have not been yet reported for this species. There is a striking resemblance between *Z. tropicalis* and *Z. jonesii* [86] in ascospores that are cylindrical and bent at the lower end. However, the ascospores of *Z. tropicalis* are longer and wider (19–26 × 3.2–6.3 μm vs 16.2–17.7 × 2.8–3.6 μm). A detailed comparison can be found in the notes to *Z. jonesii*.

**Figure 18:**
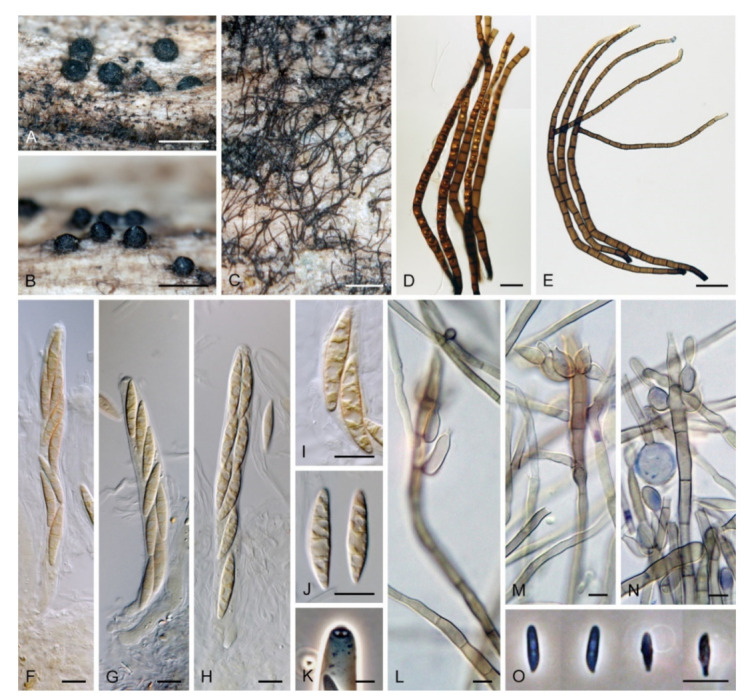
*Zanclospora xylophila* (ICMP 22737 ex-type). (**A**,**B**) Ascomata. (**C**–**E**) Stanjehughesia-like conidiophores. (**F**–**H**) Asci with ascospores. (**I**,**J**) Ascospores. (**K**) Ascal apex with an apical annulus. (**L**–**N**) Reduced *Zanclospora* conidiophores. (**O**) Microconidia. Images: (**A**–**D**) on the natural substrate; (**E**–**M**) on PCA after 8 weeks. Bars: (**A**,**B**) = 500 μm; (**C**) = 250 μm; (**D**,**L**,**M**) = 20 μm; (**E**–**K**) = 10 μm.

***Zanclospora xylophila*** Réblová & Hern.-Rest., **sp. nov.** MycoBank MB837808. (Figure 18).

Typus: NEW ZEALAND, West Coast, Westland district, Mount Aspiring National Park, Historical Bridle track, on decaying wood of a branch of *Nothofagus* sp., 31 March 2005, M. Réblová M.R. 3429/NZ 710 (holotype PDD 118748, culture ex-type ICMP 22737).

Etymology: *Xýlo* (Greek) wood, -*philous* (Greek) having an affinity, preference, from *philéō* (love), referring to wood, which the fungus inhabits.

Description on the natural substrate: Colonies effuse, hairy, dark brown, consisting of stanjehughesia-like conidiophores and ascomata. Teleomorph: Ascomata 190–230 μm diam, 220–280 μm high, superficial, solitary or in small groups, subglobose to broadly conical, papillate, dark brown to black, glabrous. Ostiole periphysate. Ascomatal wall two-layered, 30–40 μm thick; outer layer composed of dark brown, thick-walled, polyhedral cells, an inner layer composed of subhyaline to hyaline, thin-walled, polyhedral to elongated, compressed cells. Paraphyses 3–4 μm wide, hyaline, branching, anastomosing, septate. Asci 107–130(–141) × 12.5–14(–14.5) μm (mean ± SD = 120.5 ± 11.6 × 13.1 ± 0.8 μm), cylindrical-clavate, short-stipitate, apically obtuse, with a non-amyloid apical annulus 3.5–4 μm wide, 1.5–2 μm high. Ascospores 23–28(–31) × (4–)4.5–5.5(–6) μm (mean ± SD = 25.2 ± 1.4 × 5.2 ± 0.3 μm), fusiform, straight or slightly inequilateral, 3–5-septate, not constricted at the septa, hyaline, smooth, 2-seriate or obliquely 1-seriate within the ascus. Anamorph: Not observed. Synanamorph: stanjehughesia-like. Conidiophores 180–382 μm long, 5–6 μm wide above the base, 7.5–10 μm wide at the midsection, erect becoming decumbent, sinuous, unbranched, occasionally unilaterally branched, usually bent in the lower half, septate, cylindrical to cylindrical-fusiform, tapering towards the base, brown, dark brown to almost opaque at the base, paler towards the apex, apical cell light yellow-brown to subhyaline, rounded. Conidiogenous cells and conidia were not observed.

Culture characteristics: On CMD colonies 11–14 mm diam, circular, convex, margin entire, lanose, floccose, brown, darker towards the margin, reverse dark brown. On MLA colonies 21–24 mm diam, circular, slightly convex, margin entire to weakly fimbriate, lanose, cobwebby at the margin, light brown to reddish-brown at the centre, beige-brown towards the margin, with a dark brown outer zone of submerged growth, reverse dark brown. On OA colonies 4–5 mm diam, irregular, flat, margin entire, smooth to cobwebby, dark brown due to submerged growth and lack of aerial mycelium, light yellow-brown pigment diffusing into the agar, reverse dark brown. On PCA colonies 11–13 mm diam, circular, flat, margin fimbriate, lanose, floccose becoming cobwebby towards the periphery, beige at the centre, dark olivaceous brown towards the margin, reverse dark brown. Sporulation was moderate on PCA, absent on CMD, MLA and OA.

Description in PCA culture: Colonies effuse, vegetative hyphae subhyaline to light brown, septate, branched, becoming lightly encrusted, 2.5–4 μm wide, some hyphae monilioid. Anamorph: Conidiophores 70–130 × 3–4.5 μm, erect, light brown, unbranched, septate, significantly reduced. Conidiogenous cells monophialidic, 9–15 × (2.5–)3–4.5(–5) μm, tapering to 1.5–2 μm just below the collarette, integrated, terminal or discrete, laterally arranged singly or in a whorl; collarette 2–3 × 1.5–2 μm. Macroconidia absent. Microconidia (5–)6–8 × 1.5–2 μm (mean ± SD = 6.6 ± 1.0 × 1.9 ± 0.2 μm), clavate to oblong-clavate, obtuse at the base, straight or gently curved, aseptate, hyaline, smooth. Synanamorph: Not observed. Teleomorph: Not observed.

Other material examined: NEW ZEALAND, West Coast, Westland district, Kokatahi, Lake Kaniere, Dorothy Falls Road, on decaying wood of a branch, 12 Apr. 2005, M. Réblová M.R. 3492/NZ 780 (PDD 118749).

Habitat and geographical distribution: Saprobe on decaying wood of *Nothofagus* sp. and other unidentified hosts, known from New Zealand.

Notes: The *Zanclospora* conidiophores are formed only when grown in culture and in a reduced form. *Zanclospora xylophila* is similar to *Z. phaeostalacta* [85] in ascospores, but the latter species differs in having 5–7-septate and larger [(28–)30–38(–40) × 5–6(–8) μm] ascospores.

#### 3.4.2. Doubtful and Excluded Species

This section includes species retained in *Zanclospora* based on morphology but not verified by molecular DNA data, as well as species excluded or transferred to other genera on the basis of molecular evidence and/or morphological data.

***Zanclospora austroamericana*** B. Sutton & Hodges, Nova Hedwigia 26: 522. 1975.

Habitat and geographical distribution: Saprobe on the bark of *Eucalyptus propinqua*, known from Brazil [3].

Notes: For description and illustrations, see Sutton and Hodges [3]. This species closely resembles other species in the genus with typical *Zanclospora* conidiophores and falcate conidia. It can be distinguished in having simple conidiophores with conidiogenous cells confined to two separate regions.

***Zanclosporabicolorata*** R.F. Castañeda, M. Villav. & D. Sosa, Mycotaxon 135: 896. 2020.

Habitat and geographical distribution: Saprobe on decaying leaves of an unidentified plant, known from Ecuador [10].

Notes: For description and illustrations, see Villavicencio et al. [10]. *Zanclospora bicolorata* has conidiogenous cells disposed of more or less in the middle of the simple, setiform conidiophores with suballantoid conidia, similar to *Z. bonfinensis*. However, their conidiophores differ in colour and ornamentation from conidiophores of other members of the genus. The conidiophores are pale brown or brown at the base becoming dark reddish-brown to dark brown and almost opaque toward the apex. The conidiophores are smooth-walled in *Z. bicolorata*, while *Z. bonfinensis* has the conidiophores smooth at the base, becoming verrucose at the apex [8,10].

***Zanclospora bonfinensis*** D.A.C. Almeida, Gusmão & M.F.O. Marques, Mycosphere 4: 685. 2013.

Habitat and geographical distribution: Saprobe on decaying leaves of unidentified dicotyledonous plant, known from Brazil [8].

Notes: For description and illustrations, see Almeida et al. [8]. For comparison, see comment under *Z. bicolorata* and Table 2. 

***Zanclosporabrevispora*** S. Hughes & W.B. Kendr., New Zealand Journal of Botany 3: 156. 1965.

Habitat and geographical distribution: Saprobe on the bark of *Nothofagus solandri* var. *cliffortioides*, leaves of *Cocus nucifera* and on dead twigs and leaves of other unidentified substrates. It is known from Brazil, Cuba, New Zealand and South Africa [1,5,8,28].

Notes: For description and illustrations, see Hughes and Kendrick [1]. Two varieties were described under this species, *Z. brevispora* var. *brevispora* [1] and var. *transvaalensis* [5]. They are distinguished mainly by the number of conidiogenous cells and conidial characters. *Zanclospora brevispora* var. *brevispora* has more conidiogenous cells and curved somewhat smaller conidia [1,5]. This species fits well in the concept of *Zanclospora*, however further studies are needed to resolve, whether it is one or two species.

***Zanclosporaindica*** Subram. & Vittal, Can. J. Bot. 51: 1132. 1973.

Habitat and geographical distribution: Saprobe on dead leaves of *Chamaecrista desvauxii*, *Croton* sp., *Gymnosporia emarginata*, *Nectandra coriacea* and dead leaves and stems of other unidentified plants. It is known from Cuba, Brazil, India and Ivory Coast [2,8,25,91,92,93].

Notes: For description and illustrations, see Subramanian and Vittal [2]. This species deviates from the generic concept of *Zanclospora* in the morphology of the conidiogenous cells and the way they are inserted on the conidiophore. The phialides of *Z. indica* are broadly lageniform, extend into a narrow neck and elongate percurrently to form a secondary phialide. The collarette is well-defined. In addition, some of the secondary phialides are illustrated in a lateral position on the primary phialide suggesting a sympodial elongation. The phialides diverge from the conidiophore and are arranged in several whorls below the transverse septa. Other *Zanclospora* differs from *Z. indica* in having phialides with an indistinct collarette; they are tightly appressed to the conidiophore and arranged in compact fertile zones. Based on comparative morphology, *Z. indica* is not accepted in the genus.

***Zanclospora mystica*** Zucconi & Rambelli, Micologia Italiana 11: 51. 1982.

Habitat and geographical distribution: Saprobe on leaf litter, known only in Ivory Coast [4].

Notes: *Zanclospora mystica* differs from other *Zanclospora* in acute, dark brown, opaque, sterile branches inserted in the fertile zone among the phialides [4]. A similar pattern was observed in *Kionochaeta ramifera* [17,87], which differs from *Z. mystica* in a conidiogenous apparatus consisting of a compactly arranged series of subhyaline branches bearing conidiogenous cells. In other *Zanclospora* with branched conidiophores, lateral branches are similar to the main stalk and bear additional fertile zones (*Z. aurea*, *Z. falcata*, *Z. iberica*, *Z. novae-zelandiae*, *Z. lateriphiala*) or terminate into a monophialide (*Z. ramifera*). Similar branches described in *Z. mystica* also occur in *Z. stellata* (= *Stephanophorella stellata*, this study), but they are disposed at the conidiophore apex above the fertile zone.

***Brachiampulla*** Réblová & Hern.-Restr., **gen. nov.** MycoBank MB836364.

Type species: *Brachiampulla verticillata* (B. Sutton & Hodges) Réblová & Hern.-Restr.

Etymology: *Brachium* (Latin) arm, branch, *ampulla* (Latin) bottle, referring to divergent lageniform conidiogenous cells with a long extension resembling branches.

Description: Colonies effuse, dark brown, whitish-brown when sporulating, hairy. Mycelium mostly superficial, hyphae branched, septate, pigmented. Anamorph: Conidiophores macronematous, mononematous, erect, setiform, unbranched, pigmented. Conidiogenous cells polyphialidic, indeterminate, ampulliform to lageniform, elongating percurrently and sympodially, discrete, lateral, arranged singly or in whorls, or terminal, integrated. Conidia hyaline, aseptate, aggregated in slimy heads. Teleomorph: Not observed.

**Figure 19:**
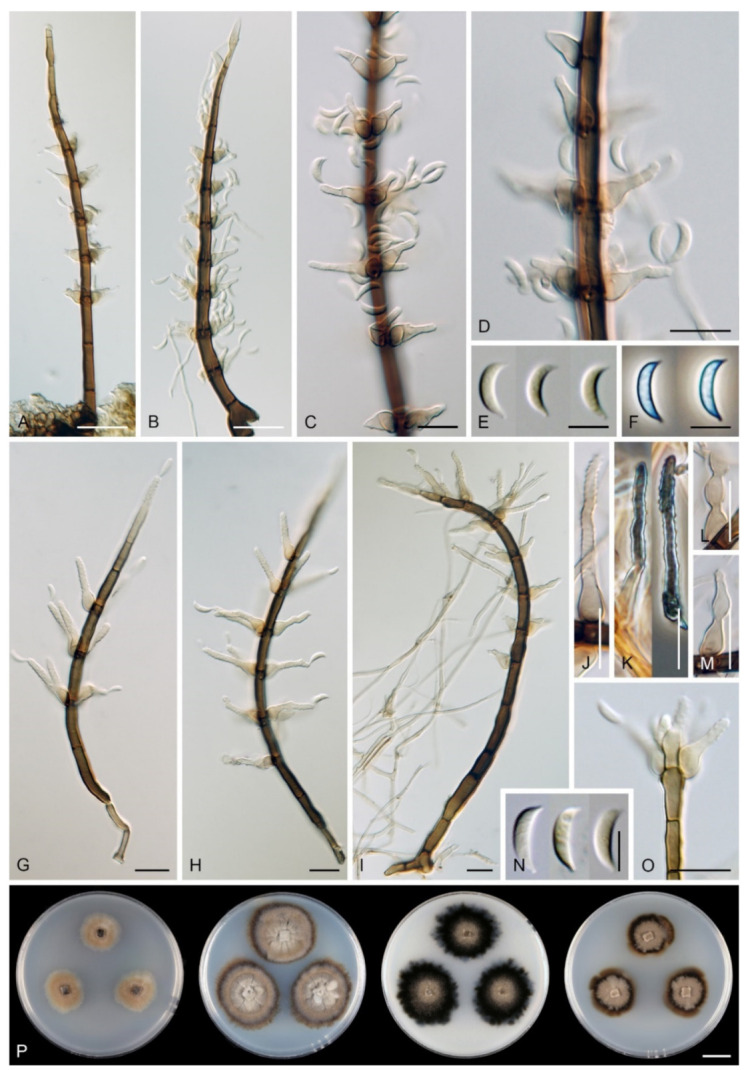
*Brachiampulla verticillata*. (**A**–**C**,**G**–**I**) Conidiophores. (**D**,**J**–**M**) Details of conidiogenous cells. (**E**,**F**,**N**) Conidia. (**O**) Apical part of the conidiophore. (**P**) Colonies on CMD, MLA, OA and PCA after 4 weeks (from left to right). Images: (**A**–**F**,**P**) on the natural substrate (ICMP 15993); (**G**–**O**) on PCA after 8 weeks (ICMP 15065 ex-paratype). Bars: (**A**,**B**) = 20 μm; (**C**–**D**,**G**–**N**) = 10 μm; (**E**,**F**,**O**) = 5 μm; (**P**) = 1 cm.

***Brachiampullaverticillata*** (B. Sutton & Hodges) Réblová & Hern.-Restr., **comb. nov.** MycoBank MB837809. (Figure 19).

Basionym: *Selenosporella verticillata* B. Sutton & Hodges, Nova Hedwigia 29: 602. 1978 (1977).

Synonym: *Zanclospora urewerae* J.A. Cooper [as “ureweri”], N. Z. J. Bot. 43: 344. 2005.

Description on the natural substrate: Colonies effuse, hairy, dark brown, whitish-brown when sporulating. Anamorph: Conidiophores 120−173 × 4−5.5(–6) μm, erect, single or in fascicles, cylindrical to subulate, base bulbose, setiform, straight or gently bent, unbranched, septate, dark brown towards the base, paler towards the apex, smooth, apical cell subhyaline to light brown, often fertile. Conidiogenous cells polyphialidic, 11−16 × 4−6 μm, venter 6–8 µm long, the upper part above venter sympodially extending 4.5−9.5 × 1.5−2 μm, with numerous lateral openings with minute collarettes, indeterminate, ampulliform to lageniform, discrete, lateral, arranged singly or in groups of 2−5 in whorls below the transverse septa, or integrated, disposed of terminally, light brown towards the base, subhyaline towards the apex, smooth, diverging from the conidiophore. Conidia 6.5−8 × 1.5−2 μm (mean ± SD = 7.2 ± 0.4 × 1.8 ± 0.2 μm), lunate, hyaline, tapering at both ends, acute apically, with a basal scar, smooth, aggregated in white slimy heads. Teleomorph: Not observed.

Culture characteristics: On CMD colonies 14–15 mm diam, circular, flat, margin finely fimbriate, cobwebby to sparsely floccose becoming mucoid towards the margin, amber-beige, with a light beige outer zone of submerged growth, reverse light brown. On MLA colonies 24–25 mm diam, circular, convex centrally, flat margin, margin finely fimbriate, lanose, floccose, furrowed at the centre, zonate, beige with a beige-brown intermediate zone, dark brown to russet at the margin, reverse dark brown. On OA colonies 23–25 mm diam, circular, flat, margin subsurface, lobate, lanose, floccose becoming mucoid towards the margin, beige-brown with a black outer zone of submerged growth, reverse black. On PCA colonies 15–17 mm diam, circular, slightly raised, margin entire, lanose, floccose becoming cobwebby to locally mucoid at the margin, beige, dark brown at the margin with a russet outer zone of submerged growth, reverse dark brown. Sporulation was abundant on CMD, OA (restricted to the inoculation block and the centre of the colony), moderate on PCA, absent on MLA.

Description on PCA: Colonies effuse, hairy, vegetative hyphae subhyaline to brown, septate, branched, 1.5–3 μm wide. Anamorph: Conidiophores, conidiogenous cells and conidia as on the natural substrate. Conidiophores 105−168 × 3.5−4.5 μm, apical cell fertile, terminated into a phialide or a whorl of several phialides. Conidiogenous cells polyphialidic, 13.5−25 × 4−6 μm, venter 6–8 μm long, the upper part above venter sympodially extending (6−)7−17.5 × 1.5−2.5 μm, occasionally with 1–2(–3) percurrent elongations, discrete, lateral, arranged singly or in groups of 2−3(−4) in whorls or integrated, terminal. Conidia 6.5−9.5 × 1.5−2 μm (mean ± SD = 7.9 ± 1.0 × 2.0 ± 0.2 μm), lunate, hyaline, aseptate. Teleomorph: Not observed.

Material examined: NEW ZEALAND, Bay of Plenty, Gisborne, Te Urewera protected area, Lake Waikaremoana, Ngamoko track (-38.76254867, 177.1541956), on a dead leaf of *Nothofagus fusca*, 11 May 2001, J.A. Cooper JAC8191 (holotype of *Z. urewerae* PDD 76621). NEW ZEALAND, Bay of Plenty, Aongetete Lodge (-37.67386195, 175.9152742), on a dead leaf of *Weinmannia racemosa*, 9 May 2003, J.A. Cooper JAC8609 (paratype of *Z. urewerae* PDD 76612, ex-paratype culture ICMP 15065). NEW ZEALAND, Manawatū-Whanganui, Erua Forest (-39.25690416, 175.326371), 4 Apr. 2005, on incubated dead leaf from a bog, J.A. Cooper JAC9549 (PDD 80888, culture ICMP 15993).

Habitat and geographical distribution: Saprobe on fallen leaves of *Eucalyptus* sp., *Nothofagus fusca*, *Weinmannia racemosa* and other unidentified hosts in New Zealand and the USA, Hawaii [7,94].

Notes: Although the species was described with conidiogenous cells with a single phialidic opening [7], the examination of the paratype, other herbarium material and living cultures revealed that the conidiogenous cells are polyphialidic, indeterminate, the upper part is sympodially elongating and contains numerous openings within minute collarettes. The holotype PDD 76621 of *Z. urewerae* did not contain any herbarium material, only a dried culture. A personal note on the holotype of *Z. urewerae* by J.A. Cooper, dated 2 July 2010, was posted on the website of the PDD herbarium and reads as: “A subsequent examination of conidiogenous cells under SEM suggests this is *Selenosporella*”.

Based on a detailed comparison of the revised material of *Z. urewerae* and the description and illustration of *Selenosporella verticillata* [94], we consider both species identical. Therefore, a new genus *Brachiampulla* is proposed for *S. verticillata* in the Xyladictyochaetaceae, and *Z. urewerae* is reduced to synonymy under the former species. *Brachiampulla verticillata* resembles *S. acicularis* [95] and *S. aristata* [96] in the morphology of conidiogenous cells with minute phialidic openings formed after sympodial elongation. On the other hand, highly similar *Selenosporella* species characterised by ampulliform, polyblastic, sympodially elongating conidiogenous cells arranged in whorls and unicellular, hyaline conidia but with holoblastic conidiogenesis include *S. curvispora* [97], the type species of *Selenosporella*, and also *S. nandiensis* [98] and *S. setosa* [99].

***Stephanophorella*** Réblová & Hern.-Restr., **gen. nov.** MycoBank MB836363.

Type species: *Stephanophorella stellata* (M. Calduch, Gené & Guarro) Réblová & Hern.-Restr.

Etymology: *Stephanos* (Greek) crown, -*phora* (Greek) bearing, from *pherein* (to bear), referring to a group of short branches resembling a crown at the conidiophore apex, -*ella*, diminutive, used as a name-forming suffix.

Description: Colonies hairy, blackish. Mycelium is mostly immersed. Anamorph: Conidiophores macronematous, mononematous, setiform, pigmented, with setiform, sterile branches inserted into the main stalk in a crown-like fashion. Conidiogenous cells phialidic, determinate, ampulliform to lageniform, with a tubular collarette, discrete, lateral, appressed to the conidiophore, arranged in whorls forming a compact fertile zone. Conidia hyaline, aseptate, slimy. Teleomorph: Not observed. (Partially adapted from Calduch et al. [6].)

**Figure 20:**
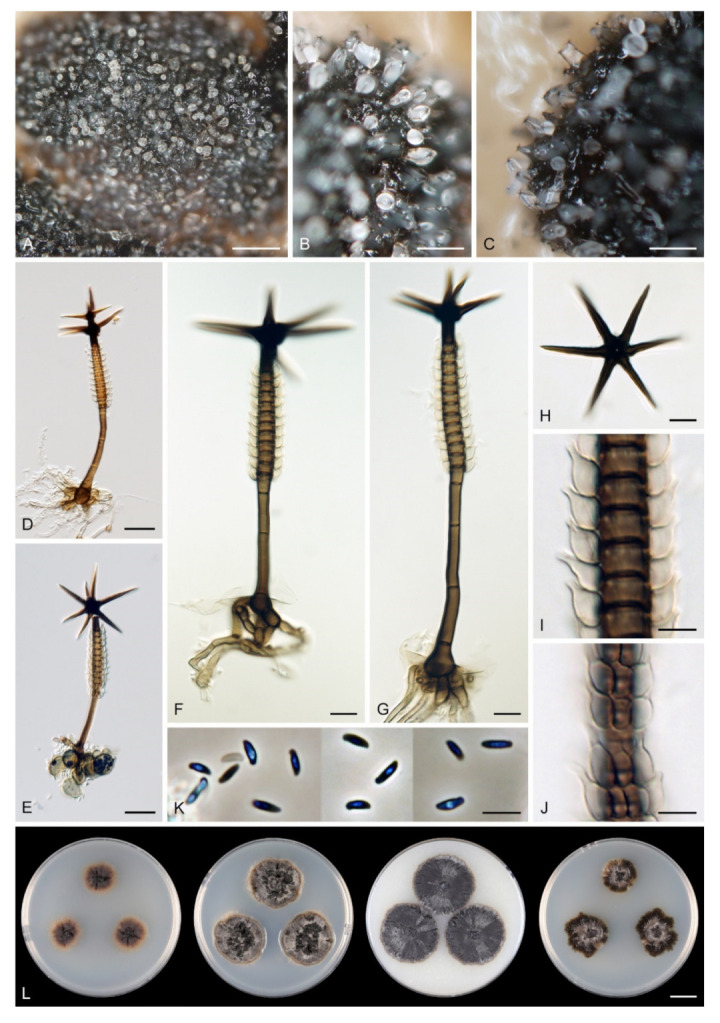
*Stephanophorella stellata* (CBS 101301 ex-type). (**A**–**C**) Colonies with conidiophores, in detail. (**D**–**G**) Conidiophores. (**H**) Branches arranged in a stellate fashion. (**I**,**J**) Conidiogenous cells. (**K**) Conidia. (**L**) Colonies on CMD, MLA, OA and PCA after 4 weeks (from left to right). Images: (**A**–**K**) on CMA after 6 weeks. Bars: (**A**) = 500 μm; (**B**,**C**) = 200 μm; (**D**,**E**) = 20 μm; (**F**–**H**) = 10 μm; (**I**–**K**) = 5 μm; (**L**) = 1 cm.

***Stephanophorella******stellata*** (M. Calduch, Gené & Guarro) Réblová & Hern.-Restr., **comb. nov.** MycoBank MB837811. (Figure 20).

Basionym: *Zanclospora stellata* M. Calduch, Gené & Guarro, Mycologia 94: 131. 2002.

Culture characteristics: On CMD colonies 14–15 mm diam, circular, raised, margin fimbriate, cobwebby, floccose becoming mucoid, deeply furrowed, dark brown, russet towards the periphery, beige at the margin, reverse dark brown. On MLA colonies 20–21 mm diam, circular, convex, margin entire, velvety to cobwebby, floccose, mucoid centrally, deeply furrowed, irregularly folded, dark brown, beige-brown towards the margin, with a dark amber-brown outer zone, reverse brown-black. On OA colonies 20–22 mm diam, circular, raised, margin lobate, mucoid, locally cobwebby to flaky, with shallow irregular folds especially at the margin, dark grey to almost black with olivaceous grey zones, reverse dark olivaceous grey. On PCA colonies 13–16 mm diam, circular, convex, flat towards the periphery, margin subsurface, fimbriate, cobwebby, floccose, furrowed centrally, smooth towards the periphery, beige-brown with a dark brown to dark amber-brown outer zone of submerged growth, reverse black. Sporulation was abundant on CMA, absent on CMD, MLA, OA and PCA.

Description on CMA: Colonies pulvinate, dark brown to black, whitish due to conidial masses on sporulating conidiophores and also covering the surface, vegetative hyphae hyaline, subhyaline to brown, branched, septate, 1.5–3 μm wide. Anamorph: Stroma well-developed at the surface of the colony, consisting of globose to polyhedral, thick-walled, subhyaline to olivaceous-brown cells, ca. 10.5–22 μm diam; cells sometimes arranged in monilioid strands. Conidiophores 125–158 μm long, 4–5.5 μm wide above the base, 6.5–8 μm wide at the fertile region, erect, setiform, pale brown at the midsection and towards the base, dark brown and opaque at the apex, septate, cylindrical to cylindrical-fusiform, base bulbose 2.5–3.5 μm wide with several rhizoids, apex smooth, sterile, acute, with up to six dark brown, opaque, acute, setiform branches arranged in a stellate formation in one or two levels at the apex. Conidiogenous cells monophialidic, 6–8.5 × 3–4 μm, tapering to ca. 1 μm below the collarette, ampulliform to lageniform, tightly appressed to the conidiophore, arranged in groups of 4–6 in 10–15 whorls just below septa; collarette flared, narrowly wedge-shaped to tubular, 1.5–2 × 2–3 μm. Conidia 3–4.5 × (0.5–)1–1.5 μm (mean ± SD = 3.4 ± 0.3 × 1.2 ± 0.1 μm), clavate to oblong-clavate to suballantoid, tapering towards the basal end, rounded at the apical end, with a conspicuous excentric inflated scar at the base, straight or inequilateral, hyaline, aseptate, smooth. Teleomorph: Not observed.

Material examined: NIGERIA, Cross River State, Mammo Forest, on unidentified dead fallen leaves, 2 Jun. 1997, M. Calduch, J. Guarro and A. M. Stchigel (culture ex-type CBS 101301 = FMR 6481).

Habitat and geographical distribution: The species is a saprobe on plant debris, known so far from Africa in Nigeria [6].

Notes: For additional description and illustrations, see Calduch et al. [6]. *Stephanophorella stellata* resembles *Zanclospora* in setiform conidiophores, lateral, determinate phialides arranged along the midsection in a fertile zone and hyaline conidia, but differs in phialides with a well-defined collarette and a crown of setiform branches at the conidiophore apex.

## 4. Discussion

### 4.1. Morphology, Interspecific Variability and Life History of Zanclospora

Our phylogenetic analysis of the combined 18S, 28S and *rpb2* sequences has provided strong support for the recognition of *Zanclospora* as a polyphyletic genus, with species distributed among three distantly related evolutionary lineages in the Sordariomycetes (Figure 1). The core of the genus, including *Z. novae-zelandiae*, clustered in the *Chaetosphaeriaceae* (Chaetosphaeriales), *Z. stellata* is positioned in the Vermiculariopsiellaceae (Vermiculariopsiellales), and *Z. urewerae* is nested in the Xyladictyochaetaceae (Xylariales).

Within the *Chaetosphaeriaceae*, *Zanclospora* is resolved as a well-supported monophyletic clade (Figure 2). It is a holomorphic genus encompassing 17 species and two varieties. Cultivation studies, morphological comparisons on natural substrates and phylogenetic analysis of four markers (ITS, 28S, *tef1-α* and *tub2*) of 21 strains representing 12 species revealed unknown pleomorphism in *Zanclospora.* Our barcoding gap analysis showed that the barcodes widely used in Ascomycota (i.e., ITS, *tef1-α* and *tub2*) are applicable for species delimitation in *Zanclospora*.

Three different conidiophore morphotypes hitherto considered unrelated, occur in the life cycle of several members of the genus. They include the anamorph with the typical *Zanclospora* conidiophores and phaeostalagmus- and stanjehughesia-like synanamorphs. Based on novel molecular and phenotypic data, the generic concept of *Zanclospora* is emended to include teleomorphic and anamorphic characters. Ten *Zanclospora* species have known teleomorph-anamorph connections, which were either experimentally established ([12], this study) or estimated, based on the juxtaposition of both morphs [11].

The diagnostic characters of *Zanclospora* include erect, pigmented, setiform conidiophores that bear one to several whorls of pale brown, sessile monophialides arising just below the septa. The phialides are appressed to the conidiophore; they form a compact fertile zone and produce hyaline, aseptate conidia without setulae. Macroconidia form only on natural conditions and vary in shape from falcate, horseshoe-shaped, obovoid to bacilliform, whereas microconidia form only in culture and are clavate to oblong-clavate, ellipsoidal to fusiform. However, the *Zanclospora* anamorphs growing on agar or natural substrate/*Urtica* stems in culture, providing semi-natural conditions, differ in size, overall complexity and appearance of conidiophores and also in conidial morphology. The synanamorph similar to *Phaeostalagmus* [19] occasionally occurs when the species is grown in culture. It shares with *Zanclospora* the arrangement of lateral phialides in whorls on the conidiophore, or they can be disposed of in a verticillate fashion on short branches. To a certain extent, it may represent a simplified *Zanclospora* under in vitro conditions. A similar analogy can be found between *Zanclospora* and the stanjehughesia-like synanamorph. The latter forms dark brown, multiseptate conidiophores, which are wider and several times longer than those of *Zanclospora*, but remain sterile, rarely with one or two lateral phialides (Figure 14A). Although conidiophores of this synanamorph are strikingly reminiscent of conidia of *Stanjehughesia* (conidiophores are absent, reduced to conidiogenous cells), a dematiaceous hyphomycete segregated from *Sporidesmium* [18], they represent a different structure with a different function. The stanjehughesia-like synanamorph has been frequently observed on natural substrates as well as in culture. *Phaeostalagmus* and *Stanjehughesia* form separate lineages in the *Chaetosphaeriaceae* tree (Figure 2).

De Hoog [100] addressed plasticity and variation in conidiogenesis of yeast-like fungi and distinguished synanamorphs into two basic categories, i.e., pleoanamorphy dependent on environmental conditions and spontaneous pleoanamorphy that exists under identical environmental conditions. The phaeostalagmus-like synanamorph was observed only when grown in culture. We assume that it may represent the group of synanamorphs that are influenced by environmental conditions.

The identification of *Zanclospora* became challenging because of the high degree of variability in the anamorphic morphology and their irregular presence on the natural substrate and in culture. This is especially true in cases when the *Zanclospora* conidiophores are absent on material from nature and form only in culture, and the stanjehughesia-like synanamorph is the only anamorphic phenotype present, i.e., *Z. clavulata*, *Z. iberica*, *Z. ramifera*, *Z. xylophila*. In addition, some strains derived from ascospores lack both *Zanclospora* and stanjehughesia-like conidiophores, and only the phaeostalagmus-like synanamorph is formed in culture, namely *Z. phaeostalacta*, *Z. sylvatica* and *Z. tropicalis*. Although the anamorph of *Z. jonesii* is unknown, brown, sinuous, cylindrical ‘setae’ arising from the base of ascomata and in their vicinity were described and illustrated by Perera et al. ([86], Figure 5g) in the protologue. These presumed setae match the stanjehughesia-like synanamorph observed in other *Zanclospora* species.

Although the stanjehughesia-like conidiophores only mimic conidia of *Sporidesmium* and its segregates, we can compare both groups in terms of the formation of phialides on these morphologically similar structures or in their life cycle. Phialidic synanamorphs are rare in *Sporidesmium* and similar taxa. *Stanjehughesia hormiscioides* (teleomorph *Umbrinosphaeria*, *Chaetosphaeriaceae*) form a chloridium-like synanamorph when grown in culture [101]. Kirk [102] described a phialidic synanamorph accompanying *Sporidesmium clarkii*; discrete phialides producing filiform, bent microconidia are born directly on conidia or separate conidiophores, solitarily or on compact branches.

In the phylogenetic tree inferred from the four combined loci (Figure 3), *Z. novae-zelandiae* and three other morphologically similar species, i.e., *Z. clavulata*, *Z. falcata* and *Z. iberica*, were resolved as closely related but separate species lineages. Except for *Z. clavulata*, which forms only microconidia, they are remarkably similar in macroconidia, conidiogenous cell and conidiophore morphology. Ornamentation of the setiform, apical part of the conidiophores of *Z. novae-zelandiae* [1], originally unique to this species, is newly described in *Z. falcata*. Moreover, the excrescences on the conidiophore surface do not develop in culture. Molecular data suggest that *Z. novae-zelandiae* is a species complex. The four-gene phylogeny and morphological comparison of specimens tentatively identified as *Z. novae-zealandiae* with the holotype of this species facilitated their correct identification and allowed interpretation of the type material. As a result, a new species, *Z. falcata*, was designated and separated from *Z. novae-zelandiae* by primarily anamorphic features and *Z. novae-zelandiae* was epitypified. Given the known broad geographical distribution and the described variability in conidial size and conidiophore characters of *Z. novae-zelandiae*, this molecular study is the first to suggest that it is an unexplored complex that may contain other cryptic species.

### 4.2. Global Biogeography of Zanclospora

*Zanclospora* was identified as a low-diverse genus comprising 12 verified species and a relatively low number of phylotypes inferred from the environmental DNA (Figure 3, Figure 5 and Figure 6). We could not link most of the phylotypes between ITS1 (6) and ITS2 (14) datasets, except for three, which contain the whole ITS, but we assume that due to their geography and ecology (Figure 7) they overlap or some represent known but so far not sequenced species. In our study, the minimal number of taxa from environmental DNA data, roughly corresponding to the level of species, was 14 (Figure 6). Data from NCBI GenBank and UNITE databases contributed to this diversity study by only one sequence (DQ124120) belonging to the phylotype ITS1-ENV5 (Figure 5). These results confirm GlobalFungi, the most comprehensive atlas of global fungal distribution, as a powerful tool for diversity studies.

Short reads attributable to *Zanclospora* were extremely rare. Theoretically, it could be due to the PCR amplification bias, resulting in an underestimation of studied fungi. In general, *Zanclospora* can be amplified and sequenced under standard conditions with commonly used ITS primers. On the contrary, data mining of sequence-only members of the *Chaetosphaeriaceae* resulted in tens of thousands of hits so PCR bias appears unlikely as an explanation for the lack of *Zanclospora* sequences in these datasets (Tables S2 and S3). We, therefore, expect that our findings reflect reality rather than being due to limitations of PCR and massively parallel sequencing.

We can further confirm that *Zanclospora* inhabits a spectrum of substrates including decaying bark, wood and fallen leaves, but also living roots and more often soil, which was not yet known. Our data mining confirms fully the pattern of distribution known from classical studies and expands its distribution and ecology. Known centres of geographical distribution (New Zealand, Central America and Caribbean, South America) were confirmed from metabarcoding data and also have shown that Southeast Asia represents another hotspot of *Zanclospora* diversity. In conclusion, based on accepted species, most of which are known from the type collection only, and data from the GlobalFungi database, we have demonstrated that *Zanclospora* is a very rare genus of worldwide distribution, living in humid natural forests (mostly temperate rainforest and tropical rainforest zones) in soil and on decaying plant matter. Our study is the first application of the GlobalFungi database for diversity, biogeography and ecology survey of a group of fungi.

The present phylogenies of metabarcoding based on ITS1 or ITS2 for the majority of sequences demonstrate the importance of environmental DNA sequences in phylogeny-based taxonomic studies. There has been much discussion in the mycological community on using metagenomic DNA from environmental samples as holotypes and in general for taxon naming [103,104,105,106,107,108,109,110]. In addition, the first studies on naming DNA-based taxa have already been published, e.g., De Beer et al. [111], Lücking and Moncada [112], Kalsoom Khan et al. [113]. The ITS1 and ITS2 phylotypes attributed to *Zanclospora* represent either new lineages or already described species that have not yet been sequenced. They expand the known geographical distribution and ecology and help to estimate the number of existing species; the number of recovered phylotypes almost doubles the number of known *Zanclospora* species. Using metabarcoding data, we gathered most information on the distribution, ecology and phylogeny of *Zanclospora* except morphology. However, *Zanclospora* includes three anamorphic phenotypes, which represent most of the morphological variability of the genus. We, therefore, prefer to define the environmental sequences of the ‘dark’ *Zanclospora* taxa as phylotypes. We hope that future sampling and increased efforts to cultivate these fascinating fungi will reveal new connections between cultivable fungi and their counterparts identified from environmental DNA.

### 4.3. Zanclospora and Its Allies

*Zanclospora* can be compared to *Cryptophiale* [15], *Cryptophialoidea* [16] and *Kionochaeta* [17], all members of the *Chaetosphaeriaceae*, which form separate lineages (Figure 2). They share pigmented, mononematous, setiform conidiophores with phialides arranged in fertile zones and hyaline conidia. The main distinguishing characters are the arrangement of phialides on the conidiophore, branching pattern of the conidiogenous apparatus and presence or absence of a shield-shaped plate. In *Cryptophiale*, *Cryptophialoidea* and *Zanclospora* the phialides are discrete, sessile, while in *Kionochaeta* they are integrated, disposed at the apex of conidiophores or branches. *Zanclospora* is the only of these four genera with phialides arranged vertically along the main axis of the conidiophore in whorls and around the entire conidiophore perimeter. The phialides have a poorly defined collarette and are not obscured by a shield or any sterile structure. *Cryptophiale*, on the other hand, encompasses fungi with the fertile region composed of sessile phialides with indistinct collarettes that arise at a right angle to the conidiophore axis, arranged in one or two palisade rows, and covered and partly enclosed by a shield-shaped plate of sterile, fused cells. The conidiophores are apically sterile, branched or unbranched, or with lateral branches. *Cryptophiale* species with phialides with a well-defined collarette arranged on only one side of the conidiophore and lacking the protecting shield were segregated into *Cryptophialoidea* (*Cr*.) by Kuthubutheen and Nawawi [16]. Molecular data, which are available only for non-type strains of *Cr. fasciculata* [114] and two *Cryptophiale* [75], suggest a close relationship of both genera. The fundamental character of *Kionochaeta* is the branching pattern of the conidiogenous apparatus. It comprises compact or loosely arranged branches bearing conidiogenous cells, irregularly branched or in a penicillate fashion. The conidiophores are unbranched or with lateral branches inserted in or above the fertile region.

In the ITS-28S phylogeny of the *Chaetosphaeriaceae* (Figure 2), the closest relatives to *Zanclospora* were *Chaetosphaeria minuta* [12] and a clade containing *Cryptophiale*, *Cryptophialoidea*, *Kionochaeta ivorensis* and *Conicomyces*. In the arrangement and morphology of phialides, the anamorph of *Ch. minuta* resembles *Cryptophialoidea* [16]. The majority of species of *Cryptophialoidea* are monophialidic, e.g., *Cr. ramosa*, *Cr. secunda*, the type species, or *Cr. uncispora* [16,114,115], mono- or rarely polyphialidic in *Cr. fasciculata* [24,114], or polyphialidic in *Cr. manifesta* [87]. Moreover, some *Cryptophialoidea* have phialides arranged in discrete unilateral bundles (*Cr. fasciculata, Cr. manifesta*, *Cr. ramosa*), while in other species the phialides are evenly distributed along the conidiophore. The conidia vary in shape and septation; they are 0–1-septate, fusiform, falcate or apically hooked. The lack of molecular data does not allow assessing the taxonomic value of these features in *Cryptophialoidea*. The separate position of *Cr. fasciculata* and *Ch. minuta* with the cryptophialoidea-like anamorph suggests further variability of this small genus, which currently includes five species [116]. The ex-type strain of *K. ivorensis* [17,117] is shown as a sister to *Cryptophiale* and *Cryptophialoidea*, while *K. ramifera* [87], the type species of the genus, and two other *Kionochaeta* form a separate, monophyletic lineage (Figure 2). The grouping of *K. ivorensis* suggests even wider phenotypic plasticity of the *Cryptophiale* clade than has been described. *Conicomyces* [118], on the other hand, is morphologically well distinguishable from *Zanclospora*. The genus was introduced for synnematous, pigmented hyphomycetes with integrated phialides and septate, setulate conidia.

In the phylogenetic analysis of the combined 18S, 28S and *rpb2* sequences (Figure 1), the ex-type strain of *Z. stellata* clustered in the Vermiculariopsiellales on a single branch basal to a clade comprising *Tubulicolla* [42] and *Vermiculariopsiella* [9]. Therefore, *Z. stellata* is excluded from *Zanclospora* into a new genus, *Stephanophorella*. Members of the Vermiculariopsiellales are saprobic, dematiaceous hyphomycetes that form effuse colonies or sporodochia. *Stephanophorella* resembles *Zanclospora* in setiform conidiophores and the arrangement of sessile, lateral phialides, but differs mainly in well-defined collarettes and the dark, opaque, setiform part of the conidiophore with branches inserted in a stellate fashion at the apex.

### 4.4. Selenosporella and Morphologically Similar Taxa

Due to the newly discovered morphological characters of conidiogenous cells, *Z. urewerae* was found to be conspecific with *Selenosporella verticillata* [94] and excluded from *Zanclospora*. A new genus *Brachiampulla* is proposed for *Z. urewerae* and its systematic placement is resolved with the four combined loci in the Xyladictyochaetaceae (Xylariales). *Brachiampulla*, based on *B. verticillata*, includes saprobes on fallen leaves, which are morphologically reminiscent of *Selenosporella*.

**Figure 21:**
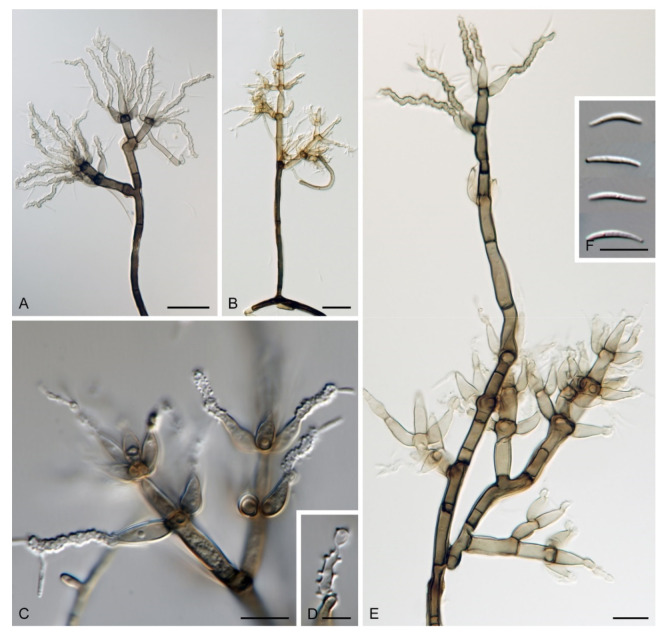
*Selenosporella curvispora* (CBS 102623). (**A**,**B**,**E**) Conidiophores. (**C**,**D**) Conidiogenous cell, in detail. (**E**) Conidia. Images: (**A**–**E**) on CMA after 8 weeks. Bars: (**A B**) = 20 μm; (**C**,**E**,**F**) = 10 μm; (**D**) = 5 μm.

The generic concept of *Selenosporella* [97,119], typified by *S. curvispora*, is morphologically heterogeneous and the genus is polyphyletic based on known morphotypes, available molecular data and known links of *Selenosporella* spp. and selenosporella-like fungi in various taxonomic groups, e.g., [20,61,67,120,121,122,123,124,125,126,127]. *Selenosporella curvispora* was described from dead leaves of *Juncus effusus* from Ireland [119] and validated by MacGarvie [97]. A non-type strain of *S. curvispora* CBS 102623 (Figure 21), collected on fallen leaves of an unidentified host in Spain was examined [on CMA: conidiophores 195–600 μm, dark brown, verticillate above; conidiogenous cells polyblastic, 11–21.5 × 3–4.5 μm (venter 7–10.5 μm long, the upper part above venter with minute denticles, sympodially extending 4–11.5 × 2–2.5 μm, elongating even more upon ageing up to 24.5 μm), venter light brown, rachis hyaline becoming light olivaceous brown; conidia 9.5–11.5 × 1, lunate, tapering apically, truncate at the base with a scar]. The placement of *S. curvispora* was confirmed in the Helminthosphaeriaceae (Figure 1). Interestingly, the *Selenosporella* phenotype is widespread in this family. The Helminthosphaeriaceae accommodate several genera with different modes of conidiogenesis such as tretic (*Diplococcium*), holoblastic (*Endophragmiella*) and holoblastic-denticulate (*Selenosporella* and selenosporella-like). Members of the family include *Helminthosphaeria* with the *Diplococcium* anamorph and several *Diplococcium* are known to form a selenosporella-like synanamorph [127,128]. Other species experimentally linked with the selenosporella-like synanamorphs belong to the family, for example, *Endophragmiella dimorphospora* [20,129], *Echinosphaeria canescens*, *Hilberina punctata* [121,122] and *Ruzenia spermoides* [61,130].

MacGarvie [97] described the conidiogenous cells of *S. curvispora* as polyphialidic, which was confirmed by Ellis [131], Sutton and Hodges [94,95,96]. In addition, Ellis [131] described the conidiogenous cells with minute protruding collarettes and coined the term ‘denticular collarette’. These observations were, however, in contrast with Matsushima [18], who interpreted the conidiogenous cells as denticulate. Onofri and Castagnola [132] studied *S. curvispora* (collection on a dead leaf from the primary rain forest, Ivory Coast) with electron microscopy and reported the conidiogenesis holoblastic-denticulate on sympodially elongating conidiogenous cells. It is difficult to observe these delicate structures on conidiogenous cells with light microscopy.

Although Sutton and Hodges [94] described the conidiogenous cells of *B. verticillata* as polyphialidic, they added that: “conidiogenesis could well be holoblastic rather than enteroblastic”.

We agree with Sutton and Hodges [94] to interpret the structures surrounding the conidiogenous loci of *B. verticillata* as minute collarettes on polyphialidic conidiogenous cells. A similar mode of conidiogenesis was described in *Xyladictyochaeta*, a sister genus of *Brachiampulla* in the Xyladictyochaetaceae [9]. It is likely that *Selenosporella* and selenosporella-like fungi accommodated in several phylogenetically different groups vary in the mode of conidiogenesis. In addition, *Selenosporella* and *Brachiampulla* share similar morphology of the conidiogenous cells that are ampulliform to lageniform, undetermined with sympodially extending apex, arranged in whorls along the conidiophore and conidia that are hyaline, unicellular, lunate or falcate.

Within the Xylariales, *Brachiampulla* is comparable to *Selenodriella* [133,134] in pigmented macronematous conidiophores, ampulliform, lateral, polyblastic conidiogenous cells arranged in whorls or terminally, and unicellular, hyaline conidia, but *Selenodriella* differs in holoblastic-denticulate conidiogenesis. *Ceratocladium* [135], represented by *C. polysetosum* [136] in our phylogeny, shares with *Brachiampulla* polyblastic, discrete, lateral, ampulliform conidiogenous cells and unicellular, hyaline conidia, but differs in the presence of setae and conidiogenous cells growing on climbing fertile hyphae. The species was resolved as a separate lineage without affinity to any known families. Its relationship to morphologically similar *Circinotrichum* was investigated by Hernández-Restrepo et al. [9]. A similar arrangement of sympodial conidiogenous cells found in *Brachiampulla* occurs, for example, in *Umbellidion* [137]. In *Umbellidion* (genera *incertae sedis*), the conidiogenous cells are cylindrical to lageniform and form only the apical whorl, occasionally the pigmented conidiophore proliferates and a second whorl is developed.

## 5. Conclusions

*Zanclospora* is pleomorphic genus rarely encountered on decaying bark, wood or leaf litter. Several species added to the genus have broadened its boundaries, but the generic concept has never been evaluated with molecular DNA data. Our knowledge of *Zanclospora* biogeography is minimal; the field records are unverified with molecular data, and only one or a handful of collections have been recorded for each species. Using six genetic markers, *Zanclospora* was shown to be polyphyletic, with three distantly related lineages in the Sordariomycetes. *Zanclospora* s. str. was resolved as a strongly supported monophyletic clade in the *Chaetosphaeriaceae*. Based on the results of phylogenetic analyses and phenotypic data, two new segregate genera, *Brachiampulla* and *Stephanophorella*, were proposed for *Z. urewerae* and *Z. stellata*, respectively. They represent two distantly related lineages in the Xylariales and Vermiculariopsiellales.

*Zanclospora* produces teleomorphs previously classified in the genus *Chaetosphaeria*. However, more frequently *Zanclospora* produces anamorphs characterised by erect, pigmented, setiform, often branched conidiophores, sessile monophialides with indistinct collarettes arranged in whorls and tightly appressed to the conidiophores or branches, and hyaline, aseptate macroconidia (on the natural substrate; falcate to horseshoe-shaped to obovoid) and microconidia (in culture; usually clavate to ellipsoidal) without setulae. Seventeen species are accepted, 12 of which have been verified with DNA sequence data. We have discovered variability in anamorphic characteristics associated with three anamorphic stages, of which phaeostalagmus- and stanjehughesia-like are newly described. Phylogenetic analyses of environmental ITS1 and ITS2 sequences retrieved from the GlobalFungi database provided insight into the global biogeography of *Zanclospora*. Seven and 15 phylotypes have been identified in samples derived from soil, dead wood and roots. The field records verified by DNA data indicated two main diversity cores in Australasia and Caribbean/Central America. Environmental ITS data suggested Southeast Asia as a third hotspot of *Zanclospora* diversity. Interestingly, environmental sequences of these fungi were completely missing in Europe and North America, which are the best-sampled continents in the GlobalFungi database.

Our study demonstrated the importance of in vitro studies to assess anamorphic plasticity and systematics of *Zanclospora* and the use of environmental sequences to expand our knowledge on biogeography and unknown interspecific diversity. It has also confirmed that different phenotypes distinguished within *Zanclospora* are phylogenetically distinct. We hope that future sampling and increased efforts to cultivate these fascinating fungi will reveal new connections between cultivable fungi and their counterparts identified from environmental DNA. Although we addressed issues related to evaluating taxonomic diagnostic criteria at the generic level, we were unable to obtain all species to assess their phylogenetic relationships. They are retained in *Zanclospora* based on morphology.

## Figures and Tables

**Table 1:** Taxa, isolate information and GenBank accession numbers for sequences. New sequences determined for this study and taxonomic novelties are given bold.

Taxon	Strain	Status	Country	Host	Substrate	GenBank					
						ITS	28S	18S	*tef1-α*	*tub2*	*rpb2*
***Brachiampulla verticillata***	ICMP 15065	P	New Zealand	*Weinmannia racemosa*	dead leaf	**MW144418**	**MW144402**	**MW151684**	**MW147322**	—	**MW147336**
***B. verticillata***	ICMP 15993		New Zealand	unidentified	dead leaf	**MW144419**	**MW144403**	**MW151685**	**MW147323**	—	**MW147337**
*Chaetosphaeria minuta*	S.M.H. 3396	T	Panama	unidentified	decaying wood	**MW144420**	AF466075	—	—	—	—
*Kionochaeta ramifera*	MUCL 39164, CBS 193.95		Cuba	unidentified	leaf	**MW144421**	**MW144404**	—	—	—	—
*Selenosporella curvispora*	CBS 102623		Spain	unidentified	decaying wood	—	**MW144405**	**MW151686**	—	—	**MW147338**
***Stephanophorella stellata***	CBS 101301, FMR 6481	T	Nigeria	unidentified	dead leaves	MH862729	MH874336	**MW151687**	—	—	**MW147339**
***Zanclospora aurea***	ICMP 23703, CBS 147013	T	New Zealand	unidentified	decaying wood	**MW144422**	**MW144406**	—	**MW147324**	**MW147343**	—
***Z. clavulata***	CBS 146967, FMR 12186	T	Portugal	unidentified	decaying wood	KY853481	KY853545	—	**MW147325**	**MW147344**	—
***Z. falcata***	ICMP 23702, CBS 147012	T	New Zealand	unidentified	decaying wood	**MW144423**	**MW144407**	**MW151688**	**MW147326**	**MW147345**	**MW147340**
*Z. iberica*	CBS 130426, FMR 11584	T	Spain	unidentified	decaying wood	KY853480	KY853544	**MW151689**	**MW147327**	**MW147346**	**MW147341**
*Z. iberica*	CBS 130280, FMR 11022		Spain	unidentified	plant debris	**MW144424**	**MW144408**	—	**MW147328**	**MW147347**	—
***Z. lateriphiala***	CBS 147014		USA	unidentified	decaying wood	**MW144425**	**MW144409**	—	**MW147329**	**MW147348**	—
***Z. lateriphiala***	ILLS121427		USA	unidentified	decaying wood	**MW144426**	**MW144410**	—	—	—	—
***Z. lateriphiala***	ILLS121428		USA	unidentified	decaying wood	JN673039	JN673039	—	—	**MW147349**	—
***Z. lateriphiala***	S.M.H. 2629-1	T	USA	unknown	decaying wood	—	AF466070	—	—	AF466031	—
***Z. lateriphiala***	S.M.H. 3320		USA	unidentified	decaying wood	**MW144427**	AF466072	—	—	AF466033	—
***Z. lateriphiala***	S.M.H. 3294		USA	unidentified	decaying wood	**MW144428**	AF466071	—	—	AF466032	—
*Z. novae-zelandiae*	ICMP 15781	E	New Zealand	*Nothofagus solandri* var. *cliffortioides*	decaying wood	**MW144429**	**MW144411**	**MW151690**	**MW147330**	**MW147350**	**MW147342**
*Z. novae-zelandiae*	ICMP 15112		New Zealand	*Nothofagus* sp.	decaying wood	**MW144430**	**MW144412**	—	**MW147331**	**MW147351**	—
***Z. phaeostalacta***	ICMP 15137, CBS 114554	T	New Zealand	unidentified	decaying wood	**MW144431**	**MW144413**	—	**MW147332**	**MW147352**	—
***Z. ramifera***	ICMP 15127		New Zealand	*Nothofagus* sp.	decaying wood	**MW144432**	**MW144414**	—	**MW147333**	**MW147353**	—
***Z. ramifera***	ICMP 22738, CBS 147101	T	New Zealand	unidentified	decaying wood	**MW144433**	**MW144415**	—	**MW147334**	**MW147354**	—
***Z. sylvatica***	S.M.H. 2893	T	Puerto Rico	unidentified	decaying wood	**MW144434**	AF279419	—	—	AF466043	—
***Z. tropicalis***	S.M.H. 1267	T	Puerto Rico	unidentified	decaying wood	**MW144435**	**MW144416**	—	—	AF466044	—
***Z. tropicalis***	S.M.H. 2250		Costa Rica	unidentified	decaying wood	**MW144436**	AF466080	—	—	AF466045	—
***Z. xylophila***	ICMP 22737	T	New Zealand	unidentified	decaying wood	**MW144437**	**MW144417**	—	**MW147335**	**MW147355**	—

Notes: T, E, P denote ex-type, ex-epitype and ex-paratype strains.

**Table 2:** A synopsis table of *Zanclospora.*

Teleomorphic Characters:							
Taxon	Substrate	Country	Teleomorph				
				Asci	Ascospores		
				Size (μm)	Size (μm)	Septation	Shape
*Z. aurea*	decaying wood	New Zealand	present	142–185(–192) × 16.5–20.5	28.5–35.5 × 7–8.5(–9)	3-septate	fusiform
*Z. austroamericana*	bark	Brazil	unknown	n/a	n/a	n/a	n/a
*Z. bicolorata*	decaying leaf	Ecuador	unknown	n/a	n/a	n/a	n/a
*Z. bonfinensis*	decaying leaves	Brazil	unknown	n/a	n/a	n/a	n/a
*Z. brevispora var. brevispora*	bark	New Zealand	present	63–75 × 5–7, stipe 10–14 × 3–5	8–10 × 3–4	1-septate	broadly ellipsoidal
*Z. brevispora var. transvaalensis*	decaying wood	South Africa	unknown	n/a	n/a	n/a	n/a
*Z. clavulata*	plant debris	Portugal	unknown	n/a	n/a	n/a	n/a
*Z. falcata*	decaying wood	New Zealand	present	(104–)112–125(–132) × 11–13.5	23.5–28.5(–30) × 4.5–5.5	3-septate	fusiform
*Z. iberica*	plant debris	Spain	unknown	n/a	n/a	n/a	n/a
*Z. jonesii*	decorticated wood	Thailand	present	69–90 × 8.5–11	16.2–17.7 × 2.8–3.6	3-septate	fusiform, bent
*Z. lateriphiala*	decaying wood	USA	present	95–113 × 10–12.5	18–24 × 4.5–6	(1–)3-septate	fusiform
*Z. mystica*	dead leaves	Ivory Coast	unknown	n/a	n/a	n/a	n/a
*Z. novae-zelandiae*	decaying wood	New Zealand	unknown	(120–)126–139(–148) × 11–12(–13)	25–29.5(–31) × 4–5	3-septate	fusiform
*Z. phaeostalacta*	decaying wood	New Zealand	present	96–127 p. sp. × 12–15(–16)	(28–)30–38(–40) × 5–6(–8)	5–7-septate	fusiform
*Z. ramifera*	decaying wood	New Zealand	present	98–125 × (10.5–)11–12.5	17–24(–25.5) × 5.5–7	3-septate	fusiform
*Z. sylvatica*	decaying wood	USA	present	95–115 × 8.7–10.7	13–20 × 4–5.5	3-septate	fusiform
*Z. tropicalis*	decaying wood	USA	present	100–138 × 10–12.5	19–26 × 3.2–6.3	3-septate	fusiform, bent
*Z. xylophila*	decaying wood	New Zealand	present	107–130(–141) × 12.5–14(–14.5)	23–28(–31) × (4–)4.5–5.5(–6)	3–5-septate	fusiform
Anamorphic Characters:							
**Taxon**	***Zanclospora* Anamorph**						
			**Conidiophores**			**Macroconidia**	
	**On Natural Substrate (μm)**		**Branches**	**Apex**	**In Culture (μm)**	**Size (μm)**	**Shape**
*Z. aurea*	478–568 × 4.5–6, 5–6.5 at FZ		present, similar to main stalk	smooth	absent	15–23(–24) × 3–4.5	falcate/ horseshoe-shaped
*Z. austroamericana*	up to 260 × 6		absent	smooth	n/a	12–19 × 2–3	falcate
*Z. bicolorata*	not observed		absent	smooth	≤ 300 × 10–17	2–4 × 1–1.5	suballantoid
*Z. bonfinensis*	110–210 × 3.5–6		absent	verrucose	n/a	3–5.5 × 1–2	bacilliform
*Z. brevispora var. brevispora*	100–175(–220) × 5.4–7		absent	smooth	n/a	5.4–8(–9.4) × 1.4–2	narrowly obovoid
*Z. brevispora var. transvaalensis*	up to 140 × 10, 5–6 at FZ		absent	smooth	n/a	8–10 × 2.5	narrowly obovoid to clavate
*Z. clavulata*	not observed		absent *	smooth*	33–68 × 3–4, 3–4 at FZ	not observed	n/a
*Z. falcata*	210–350(–520) × 5–7.5, 6–8 at FZ		present, similar to main stalk	with excrescences	80−120 × 5−6(−7.5), 7−8 at FZ	18–24.5 × 2.5–3	falcate
*Z. iberica*	not observed		present, similar to main stalk *	smooth*	128–318 × 4.5–6, 6.5–9 at FZ	(12.5–)15.5–25 × 2–3 *	falcate
*Z. jonesii*	not observed		n/a	n/a	n/a	n/a	n/a
*Z. lateriphiala*	240–284 × 5–6.5		present, similar to main stalk	smooth	56–120 × 2.5–3	15–25 × 2.5–4	falcate
*Z. mystica*	135–175 × 4.5		present, setiform	smooth	n/a	12.5–16.5 × 1.5–2.5	falcate
*Z. novae-zelandiae*	360–450 × 5.5–7, 5.5–6.5 at FZ		present, similar to main stalk	with excrescences	277–380 × 4.5–5.5(–6), 6.5–8 at FZ	24–28.5 × (2–)2.5–3 16–24 × (1.5–)2–3 *	falcate
*Z. phaeostalacta*	not observed		n/a	n/a	n/a	n/a	n/a
*Z. ramifera*	not observed		present, similar to main stalk	smooth	(30–)55–235 × 2–3.5, 3.5–6 at FZ	n/a	n/a
*Z. sylvatica*	not observed		n/a	n/a	n/a	n/a	n/a
*Z. tropicalis*	not observed		n/a	n/a	n/a	n/a	n/a
*Z. xylophila*	not observed		n/a	smooth	n/a	n/a	n/a
Synanamorphic Characters:							
**Taxon**	**stanjehughesia-like Synanamorph**			*Zanclospora* Conidiophores or phaeostalagmus-like Synanamorph In Vitro **			
	**Conidiophores**			**Conidiophores**	**Microconidia**		
	**On Natural Substrate (μm)**		**In Culture (μm)**		**Size (μm)**	**Shape**	**Reference**
*Z. aurea*	not observed		not observed	present	5–6 × 1.5–2.5	clavate	This study
*Z. austroamericana*	not observed		n/a	n/a	n/a	n/a	[3]
*Z. bicolorata*	not observed		n/a	n/a	n/a	n/a	[10]
*Z. bonfinensis*	not observed		n/a	n/a	n/a	n/a	[8]
*Z. brevispora var. brevispora*	not observed		n/a	n/a	n/a	n/a	[1]
*Z. brevispora var. transvaalensis*	not observed		n/a	n/a	n/a	n/a	[5]
*Z. clavulata*	not observed		135–155 × 3–3.5, 6–7 at MS	present	3.5–7 × 0.7–1	clavate to fusiform	This study
*Z. falcata*	155–270 × (4–)5–6.5, (9–)11–13 at MS		210–310 × 4.5–6.5, 8.5–11 at MS	present	5−6 × 1(–1.5)	clavate to oblong-clavate	This study
*Z. iberica*	83–180 × 9–11		340–660(–920) × (3.5)4.5–6, 9–14 at MS	present	(5–)6–10.5 × 1.5–2	clavate to oblong-clavate	[9]
*Z. jonesii*	present, described as setae		n/a	n/a	n/a	n/a	[86]
*Z. lateriphiala*	not observed		not observed	present	not observed	not observed	[12]
*Z. mystica*	not observed		n/a	n/a	n/a	n/a	[4]
*Z. novae-zelandiae*	364–675 × 4.5–5, 8–10.5 at MS		605–800 × 5–5.5, 6.5–7.5 at MS	present	5–8 × 1.5–2	clavate to oblong-clavate	This study
*Z. phaeostalacta*	not observed		not observed	present	3–7 × 2–2.5	ellipsoidal, apiculate	[85]
*Z. ramifera*	155–195 × 4–5, 10.5–12(–15) at MS		53–154 × 3–3.5, 3.5–7.5 at MS	present	5–6 × 1.5	clavate to oblong-clavate	This study
*Z. sylvatica*	not observed		not observed	present	5–8 × 1.2–1.5	cylindrical-clavate	[12]
*Z. tropicalis*	not observed		not observed	present	5–8 × 1.2–1.5	narrowly oblong	[12]
*Z. xylophila*	180–382 × 5–6, 7.5–10 at MS		not observed	present	(5–)6–8 × 1.5–2	clavate to oblong-clavate	This study

Notes: * Macroconidia and conidiophores in culture; ** Strongly reduced *Zanclospora* conidiophores or conidiophores reminiscent of phaeostalagmus-like formed in vitro; MS—Midsection of the stanjehughesia-like conidiophores; FZ—Fertile zone of the *Zanclospora* conidiophores.

## Data Availability

All sequences generated in this study were submitted to GenBank (ITS: MW144418–MW144437; 28S: MW144402–MW144417; 18S: MW151684–MW151690; *tef1-α*: MW147322–MW147335; *rpb2*: MW147336–MW147342; *tub2*: MW147343–MW147355).

## Supplementary Materials

The following are available online at https://www.mdpi.com/article/10.3390/microorganisms9040706/s1, Table S1: Taxa of the Sordariomycetes, their collection numbers and accession numbers for sequences retrieved from GenBank, Table S2: A list of environmental samples attributable to *Zanclospora* with references to all studies in the GlobalFungi database, Table S3: Taxa related to *Zanclospora* and comparison of their BLAST search in the GlobalFungi database, Table S4: Estimates of evolutionary divergence between ITS rDNA, 28S rDNA, *tub2* and *tef1-α* sequences, Table S5: The biogeography, substrate and habitat affinity of *Zanclospora* and outgroup taxa inferred from the GlobalFungi database, Table S6: Published records of *Zanclospora* with host, substrate, country of the collection and references.
