# Supplementary material for: Phylogeny, Global Biogeography and Pleomorphism of Zanclospora
Source: Microorganisms. 2021 Mar 29;9(4):706. doi: 10.3390/microorganisms9040706 (PMC8066784; doi:10.3390/microorganisms9040706)
Supplement: Supplementary file 1 [file microorganisms-09-00706-s001.zip › Supplementary Tables/Supplementary Table S1.pdf]

Table S1. Taxa of the Sordariomycetes, their collection numbers and accession numbers for sequences retrieved from GenBank.

Taxon	Strain	Status	GenBank accession numbers				
			ITS	28S	18S	*tef1-α*	*rpb2*
Acrocordiella occulta	CBS 140500	E	KT949893	KT949893	—	—	—
Adautomilanezia caesalpiniae	CC-LAMIC 102/12	T	KX821777	KU170671	—	—	—
Adelosphaeria catenata	CBS 138679	T	—	KT278707	KT278692	—	KT278743
Achaetomium macrosporum	CBS 532.94		—	KX976699	—	—	KX976797
Amphibambusa bambusicola	MFLUCC 11-0617	T	KP744433	KP744474	—	—	—
Amphisphaeria flava	MFLUCC 18-0361	T	MH971224	MH971234	—	—	—
Amphisphaeria thailandica	MFLU 18-0794	T	MH971225	MH971235	—	—	MK033640
Anacacumisporium appendiculatum	HMAS 245593	T	KP347129	KT001553	—	—	—
Annulohypoxylon michelianum	CBS 119993		KX376320	KY610423	—	—	KY624234
Annulusmagnus triseptatus	CBS 131483		—	GQ996540	JQ429242	—	JQ429258
Anthostoma decipiens	CBS 133221		KC774565	KC774565	—	—	—
Anungitiomyces stellenboschiensis	CPC 34726	T	MK876376	MK876415	—	—	—
Apiorhynchostoma curreyi	UAMH 11088		—	JX460989	KY931894	—	KY931926
Arthrinium hysterinum	ICMP 6889		MK014874	MK014841	—	MK017951	DQ368649
Arthrinium pseudoparenchymaticum	SICAUCC 18-0008		MK346319	MK346321	—	MK359205	MK359207
Ascitendus austriacus	CBS 131685		—	GQ996539	GQ996542	—	JQ429257
Ascovaginospora stellipala	P513A	T	—	U85088	U85087	—	—
Astrosphaeriella erumpens	S.M.H. 1291			AF279410	—	—	AY641073
Atractosphaeria verruculosa	CBS 132040	T	—	KT991659	KT991641	—	KT991648
Atractosphaeria decumbens	CBS 139032	T	—	KT991658	KT991640	—	KT991647
Bactrodesmium abruptum	CBS 145967		—	MN699410	MN699367	MN704315	MN704290
Bactrodesmium diversum	CBS 142448		—	MN699412	MN699369	MN704317	MN704292
Barbatosphaeria barbirostris	CBS 121149		—	EF577059	KM492851	—	KM492903
Barbatosphaeria dryina	CBS 127691		—	KM492864	KM492852	—	KM492904
Barrmaelia macrospora	CBS 142768	T	KC774566	KC774566	—	MF489005	MF488995
Barrmaelia moravica	CBS 142769	E	MF488987	MF488987	—	MF489006	MF488996
Beltrania pseudorhombica	CBS 138003	T	MH554124	KJ869215	—	MH554558	MH555032
Beltraniopsis neolitseae	CBS 137974	T	KJ869126	KJ869183	—	—	—
Biscogniauxia nummularia	MUCL 51395	E	KY610382	KY610427	—	—	KY624236
Bombardia bombarda	AFTOL-ID 967		—	DQ470970	DQ471021	—	DQ470923
Brunneodinemasporium brasiliense	CBS 112007	T	JQ889272	JQ889288	—	—	—
Brunneodinemasporium jonesii	GZCC 16-0050	T	KY026058	KY026055	—	—	—
Brunneosporella aquatica	HKUCC 3708		—	AF132326	—	—	—
Cacumisporium capitatum	FMR 11339		—	HF677176	—	—	—
Cainia anthoxanthis	MFLUCC 15-0539	T	KR092787	KR092777	—	—	—
Calosphaeria pulchella	CBS 115999	T	—	AY761075	AY761071	—	GU180661
Calvolachnella guaviyunis	CBS 134695	T	KJ834524	KJ834525	—	—	—
Camarops microspora	CBS 649.92		—	AY083821	DQ471036	—	DQ470937
Camillea obularia	ATCC 28093		AF201714	KY610429	—	—	—
Castanediella acaciae	CBS 139896	T	KR476728	MH878661	—	—	—
Castanediella cagnizarii	MUCL 41095		KC775732	KC775707	—	KJ476985	—
Castanediella ramosa	MUCL 39857		KC775736	KC775711	—	KJ476989	—
Catenularia cubensis	MFLUCC 18-1331		MK828638	MK835840	—	—	—
Chaetosphaeria catenulata	S-891	T	—	MK835838	MK834746	—	MN156523
Chaetosphaeria curvispora	CBS 113644		—	GU180636	AY502933	—	GU180655
Chaetosphaeria chlorotunicata	S.M.H. 1565	T	—	AF466064	—	—	—
Chaetosphaeria innumera	M.R. 1175		AF178551	AF178551	—	—	—
Chaetosphaeria mangrovei	MCD 069		MG813821	MG813820	—	—	—
Chaetosphaeria myriocarpa	CBS 264.76		AF178552	AF178552	—	—	—
Chaetosphaeria pygmaea	M.R. 1365		AF178545	AF178545	—	—	—
Chaetosphaeria submersa	MFLUCC 18-1342	T	MK828634	MK835835	—	—	—
Chalara breviclavata	S.M.H. 2018		—	AY017372	—	—	—
Chloridium caesium	CBS 102339		AF178564	AF178564	—	—	—
Chloridium gonytrichii	MFLUCC 16-1095		—	MK835822	MK834737	—	MN156516
Chloridium virescens	CBS 152.53		MH857142	MH868678	—	—	—
Cephalotheca foveolata	IFM 53377, UAMH 10952		—	AB178269	AB278171	—	KC408407
Ceratocladium polysetosum	FMR 10750	T	KY853430	KY853490	—	—	—
Ceratostomella cuspidata	ICMP 17629		—	FJ617558	KT991642	—	KT991651
Ceratostomella pyrenaica	CBS 129343		—	KY931835	KY931893	—	KY931863
Clypeophysalospora latitans	CBS 141463	T	KX820250	KX820261	—	—	—
Codinaea assamica	CBS 242.66		MH858788	MH870426	—	—	—

Codinaea lambertiae	CBS 143419	T	MG386052	MG386105	—	—	—
Codinaea simplex	CBS 966.69		AF178559	AF178559	—	—	—
Codinaeopsis gonytrichodes	CBS 593.93		AF178556	AF178556	—	—	—
Collodiscula japonica	CBS 124266		JF440974	JF440974	—	—	—
Conicomyces pseudotransvaalensis	HHUF 29956	T	LC001710	LC001708	—	—	—
Coniocessia cruciformis	CBS 125769	T	MH863750	MH875218	—	—	—
Coniocessia minima	CBS 125765	T	MH863746	MH875214	—	—	—
Coniochaeta discoidea	SANK 12878		—	AY346297	—	—	AY780191
Coniochaeta ostrea	CBS 507.70		—	DQ470959	DQ471007	—	DQ470909
Cordana abramovii	MFLU 18-1454		—	MK835800	MK834763	—	—
Cordana lignicola	MFLUCC 17-1332	T	—	MK835797	MK834761	—	MN156527
Cryptendoxyla hypophloia	FR 58		—	AF096190	AF096175	—	—
Cryptophiale hamulata	MFLU 17-1975	T	—	MG386756	—	—	—
Cryptophiale udagawae	GZCC 18-0047		MN104608	MN104619	—	—	—
Cryptophialoidea fasciculata	MFLU 18-1499		MH758195	MH758208	—	—	—
Cryptosphaeria eunomia var. fraxini	CBS 223.87		KT425231	KT425295	—	—	KT425361
Cryptovalsa rabenhorstii	CBS 125574		KC774567	KC774567	—	—	—
Cylindrium elongatum	CBS 115974		KM231853	KM231733	—	KM231989	KM232429
Cylindrium grande	CBS 145578		MK876385	MK876426	—	MK876496	MK876482
Daldinia concentrica	CBS 113277		AY616683	KY610434	—	—	KY624243
Delonicicola siamense	MFLUCC 15-0670	T	MF167586	MF158345	—	—	MF158346
Dendrophoma cytisporoides	CBS 144107		MT118234	MT118205	—	—	—
Diaporthe phaseolorum	FAU 458, NRRL 13736		—	U47830	L36985	—	AY641036
Diaporthe disciformis	CBS 197.49			DQ470964	DQ471012	DQ471085	DQ470915
Diatrypella vulgaris	CBS 128329		MH864880	MH876328	—	—	—
Dictyochaeta callimorpha	ICMP 15130		MT454483	MT454498	—	—	—
Dictyochaeta cangshanensis	MFLUCC 17-2214	T	MK828632	MK835832	—	—	—
Dictyochaeta ellipsoidea	MFLUCC 18-1574	T	MK828628	MK835828	—	—	—
Dictyochaeta fuegiana	ICMP 15153	T	MT454487	EF063574	—	—	—
Dictyochaeta querna	CBS 145503		MT454489	MT454503	—	—	—
Dictyochaeta septata	CBS 143386	E	MH107889	MH107936	—	—	—
Dictyochaeta terminalis	GZCC 18-0085	T	MN104613	MN104624	—	—	—
Dinemasporium americanum	CBS 127127	T	JQ889274	JQ889290	—	—	—
Dn.pseudoindicum	CBS 127402	T	JQ889277	JQ889293	—	—	—
Distoseptispora adscendens	HKUCC 10-820		—	DQ408561	—	—	DQ435092
Distoseptispora leonensis	HKUCC 10-822		—	DQ408566	—	—	DQ435089
Echinosphaeria canescens	S.M.H. 4791		—	AY436403	—	—	—
Ellisembia aurea	CBS 144403	T	MH836375	MH836376	—	—	—
Endophragmiella dimorphospora	FMR 12150		—	KY853502	HF937351	—	—
Endoxyla operculata	UAMH 11085		—	JX460992	KY931895	—	KY931927
Entosordaria perfidiosa	CBS 142773	E	MF488993	MF488993	—	MF489012	MF489003
Eucalyptostroma eucalypti	CBS 142074	T	KY173408	KY173500	—	—	—
Exserticlava vasiformis	TAMA 450		—	AB753846	—	—	—
Fasciatispora arengae	MFLUCC 15-0326a		MK120275	MK120300	—	MK890790	MK890794
Fasciatispora cocoes	MFLUCC 18-1445		MN482680	MN482675	—	MN481516	MN481517
Fragosphaeria purpurea	CBS 133.34		—	AF096191	AF096176	—	—
Furfurella luteostiolata	CBS 143620	T	MK527842	MK527842	—	MK523302	MK523273
Gaeumannomyces graminis	AR 3401, M 57		—	AF362557	JF414874	—	—
Gelasinospora tetrasperma	CBS 178.33		—	DQ470980	DQ471032	—	DQ470932
Graphostroma platystoma	CBS 270.87		JX658535	DQ836906	—	DQ836915	KY624296
Helicoascotaiwania lacustris	CBS 145963	T	—	MN699430	MN699382	MN704329	MN704304
Helminthosphaeria carpathica	S.M.H. 3908		—	AY346282	—	—	—
Helminthosphaeria clavariarum	S.M.H. 4609		—	AY346283	—	—	—
Helminthosphaeria corticiorum	J.F. 04225		—	KF765607	—	—	—
Helminthosphaeria mammillata	A.N.M. 986		—	KF765609	—	—	—
Helminthosphaeria odontiae	A.N.M. 928		—	KF765610	—	—	—
Helminthosphaeria tomaculum	S.M.H. 2485	T	—	KF765613	—	—	—
Helminthosphaeria triseptata	J.F. 04015		—	KF765614	—	—	—
Hilberina caudata	S.M.H. 1542		—	KF765615	—	—	—
Hilberina munkii	S.M.H. 1531		—	KF765616	—	—	—
Hypocopra rostrata	NRRL 66178		KM067909	KM067909	—	—	—
Hyponectria buxi	UME 31430			AY083834	—	—	—
Hypoxylon fragiforme	MUCL 51264	E	KC477229	KM186295	—	—	KM186296
Idriella lunata	CBS 204.56	T	KP859044	KP858981	—	—	—

Induratia thailandica	MFLUCC 17-2669	T	MK762707	MK762714	—	—	MK791283
Infundibulomyces cupulatus	BCC 11929	T	EF113976	EF113979	—	—	—
Infundibulomyces oblongisporus	BCC 13400	T	EF113977	EF113980	—	—	—
Iodosphaeria honghensis	MFLU 19-0719	T	MK737501	MK722172	—	—	MK791287
Iodosphaeria tongrenensis	MFLU 15-0393		KR095282	KR095283	—	—	—
Jattaea algeriensis	CBS 120871	T	—	EU367457	EU367462	—	HQ878603
Jobellisia fraterna	S.M.H. 2863		—	AY346285	—	—	—
Jobellisia luteola	S.M.H. 2753		—	AY346286	—	—	—
Kionochaeta castaneae	GZCC 18-0025	T	MN104610	MN104621	—	—	—
Kionochaeta ivoriensis	CBS 374.76	T	MH860988	MH872758	—	—	—
Kionochaeta microspora	GZCC 18-0036	T	MN104607	MN104618	—	—	—
Kramasamuha sibika	CPC 36725		—	MN794356	—	—	—
Kretzschmaria deusta	CBS 163.93		KC477237	KY610458	—	—	KY624227
Lanspora coronata	J.K. 4839A		—	U46889	DQ470996	—	DQ470899
Lasiosphaeria ovina	S.M.H. 1538, CBS 958.72		—	AF064643	AY083799	—	AY600292
Leiosphaerella praeclara	CBS 125586		JF440976	JF440976	—	—	—
Lentomitella vestita	PRA-12739		—	KY931820	KY931879	—	KY931848
Lepteutypa fuckelii	CBS 140409	T	KT949902	KT949902	—	MH554435	MH554918
Leptosillia acerina	CBS 143939	E	MK527849	MK527849	—	MK523310	MK523282
Leptosillia macrospora	CBS 143627	E	MK527853	MK527853	—	MK523314	MK523286
Leptosporella arengae	MFLUCC 15-0330	T	MG272255	MG272246	—	—	—
Leptosporella bambusae	MFLUCC 12-0846	T	KU940134	KU863122	—	—	—
Leptosporella gregaria	S.M.H. 4867		—	HM171288	—	—	—
Lopadostoma gastrinum	CBS 134632	N	KC774584	KC774584	—	—	—
Lopadostoma turgidum	CBS 133207	E	KC774618	KC774618	—	—	KC774563
Macgarvieomyces borealis	CBS 461.65	T	—	DQ341511	DQ341489	—	—
Magnaporthe grisea	Ina168, 70-15		—	AB026819	DQ493955	—	—
Melanotrigonum ovale	CBS 138743	T	—	KT278709	KT278696	—	KT278745
Melogramma campylosporum	CBS 141086		JF440978	JF440978	—	—	—
Menispora ciliata	CBS 122131, ICMP 18253	T	EU488736	GU180637	GU180614	—	GU180659
Menispora tortuosa	DAOM 231154		KT225527	AY544682	—	—	—
Menisporopsis dushanensis	GZCC 18-0084	T	MN104615	MN104626	—	—	—
Menisporopsis theobromae	MFLUCC 15-0055		KX609957	KX609954	—	—	—
Microdochium lycopodinum	CBS 125585	T	JF440979	JF440979	—	—	KP859125
Mirannulata samuelsii	S.M.H. 1880	T	—	AY578353	—	—	—
Multiguttulispora sympodialis	MFLUCC 18-0153	T	MN104606	MN104617	—	—	—
Muscodor yunnanensis	WS38		MG866046	MG866038	—	—	MG866059
Myrmecridium flexuosum	CBS 398.76	T	—	EU041825	—	—	—
Myrmecridium schulzeri	CBS 100.54		—	EU041826	—	—	—
Nawawia filiformis	MFLUCC 17-2394		MH758196	MH758209	—	—	—
Neophysalospora eucalypti	CBS 138864	T	KP004462	KP004490	—	—	—
Neopseudolachnella acutispora	MAFF 244358	T	AB934065	AB934041	—	—	—
Neopseudolachnella magnispora	MAFF 244359	T	AB934066	AB934042	—	—	—
Neotracylla pini	CBS 146010	T	—	MN567636	—	—	—
Neurospora calospora	CBS 198.55		—	MH868987	—	—	—
Nothodactylaria nephrolepidis	CBS 146078	T	MN562132	MN567639	—	—	MN556809
Ophiostoma piliferum	CBS 158.74		—	DQ470955	DQ471003	—	DQ470905
Oxydothis metroxylonicola	MFLUCC 15-0281	T	KY206774	KY206763	KY206769	KY206778	KY206781
Oxydothis palmicola	MFLUCC 15-0806	T	KY206776	KY206765	—	KY206780	KY206782
Paliphora intermedia	CBS 896.97	I	MH862682	EF204501	—	—	—
Papulosa amerospora	J.K. 5547F		—	DQ470950	DQ470998	—	DQ470901
Phaeoacremonium fraxinopennsylvanicum	CBS 128920		—	HQ878595	HQ878600	—	HQ878609
Phaeoacremonium minimum	CBS 246.91	T	—	AB278174	AB278174	—	—
Phaeostalagmus cyclosporus	CBS 663.70		MH859892	MH871680	—	—	—
Phialosporostilbe scutiformis	MFLUCC 17-0227	T	MH758194	MH758207	—	—	—
Phlogicylindrium eucalypti	CBS 120080	T	DQ923534	DQ923534	—	—	MH554893
Phlogicylindrium uniforme	CBS 131312	T	JQ044426	JQ044445	—	—	—
Phomatospora bellaminuta	J.K. 5543N		—	FJ176857	FJ176803	—	FJ238345
Phyllachora chloridis	MFLU 15-0173		—	MF197499	MF197505	—	—
Pleurostoma ootheca	CBS 115329	T	—	AY761079	AY761074	—	HQ878606
Pleurostoma richardsiae	CBS 270.33	T	—	AY761080	AY761066	—	HQ878607
Pleurotheciella erumpens	CBS 142447	T	—	MN699435	MN699387	MN704334	MN704311
Polynema podocarpi	CBS 144415	T	MH327797	MH327833	—	—	—
Pseudapiospora corni	CBS 140736	N	KT949907	KT949907	—	—	—

Pseudodactylaria brevis	MFLUCC 16-0032	T	—	MH262310	—	—	—
Pseudodactylaria xanthorrhoeae	CBS 143414	T	—	MG386117	—	—	—
Pseudodinemasporium fabiforme	CBS 140010	T	KR611889	KR611906	—	—	—
Pseudolachnea fraxini	CBS 113701	T	JQ889287	JQ889301	—	—	—
Pseudolachnea hispidula	MAFF 244365		AB934072	AB934048	—	—	—
Pseudolachnella asymmetrica	MAFF 244366		AB934073	AB934049	—	—	—
Pseudolachnella scolecospora	MAFF 244379		AB934086	AB934062	—	—	—
Pseudomassaria chondrospora	CBS 125600		JF440981	JF440981	—	—	—
Pseudosporidesmium lambertiae	CBS 143169	T	MG386034	MG386087	—	—	—
Pseudotruncatella arezzoensis	MFLUCC 14-0988	T	MG192320	MG192317	—	—	—
Pseudotruncatella bolusanthi	CBS 145532	T	MK876407	MK876448	—	—	—
Pyrigemmula aurantiaca	CBS 126743	T	HM241692	HM241692	—	—	—
Raffaelea ambrosiae	CBS 185.64		—	EU984297	AY497518	—	—
Rattania setulifera	GUFCC 15-501	T	GU191794	HM171322	—	—	—
Requienella seminuda	CBS 140502		KT949912	KT949912	—	MK523328	MK523300
Rhamphoria pyriformis	CBS 139024		—	MG600397	MG600405	—	MG600401
Rhamphoriopsis muriformis	CBS 127683		—	MG600395	MG600403	—	MG600399
Rhodoveronaea varioseptata	CBS 123473		—	FJ617560	JX066710	—	JX066700
Robillarda sessilis	CBS 114312	E	KR873256	KR873284	—	—	—
Ruzenia spermoides	CBS 101621		—	AY436421	—	—	—
Seiridium marginatum	CBS 140403	E	KT949914	KT949914	—	MK523329	MK523301
Selenodriella cubensis	CBS 683.96	T	KP859053	KP858990	—	—	—
Selenodriella fertilis	CBS 772.83		KP859055	KP858992	—	—	—
Spadicoides hyalostoma	CBS 137793		—	KY931828	KY931885	—	KY931855
Sporidesmium minigelatinosa	NN 47497		—	DQ408567	—	—	DQ435090
Sporidesmium parvum	HKUCC 10836		—	DQ408558	—	—	—
Sporoschisma longicatenatum	MFLUCC 16-0180	T	KX505871	KX358077	—	—	—
Sporoschisma mirabile	FMR 11247		HF677174	HF677183	—	—	—
Stanjehughesia hormiscioides	CBS 102664		—	AF261069	—	—	—
Strelitziomyces knysnanus	CBS 146056	T	MN562135	MN567642	—	—	MN556810
Striatosphaeria castanea	CBS 145352	T	MT118244	MT118229	—	—	—
Striatosphaeria codinaeophora	M.R. 1230		AF178546	AF178546	—	—	—
Subsessila turbinata	MFLUCC 15-0831	T	KX762288	KX762289	—	KX762291	—
Synaptospora plumbea	A.N.M. 963		—	KF765620	—	—	—
Tainosphaeria jonesii	GZCC 16-0065	P	KY026060	KY026057	—	—	—
Tainosphaeria siamensis	MFLUCC 15-0607	T	KX609956	KX609953	—	—	—
Teracosphaeria petroica	ICMP 15111	T		EF063576	MT454479	—	MT454670
Thozetella fabacearum	MFLU 16-1021	T	KY212754	KY212762	—	—	—
Thozetella tocklaiensis	CBS 378.58	T	MH857817	MH869349	—	—	—
Togniniella acerosa	CBS 113648		—	AY761076	AY761073	—	GU180660
Torrentispora dubia	PRA-12746		—	KY931832	KY931889	—	—
Tracylla aristata	CBS 141404	E	KX306770	KX306795	—	—	—
Tracylla eucalypti	CBS 144429	T	MH327810	MH327846	—	—	—
Tubulicolla cylindrospora	MUCL 39171		—	EF063575	MT454478	—	MT454669
Valsa ambiens	AR 3516		—	AF362564	DQ862056	—	DQ862025
Vermiculariopsiella acaciae	CBS 141289	T	—	KX228314	—	—	—
Vermiculariopsiella dunnii	CBS 145538	T	—	MK876452	—	—	—
Vermiculariopsiella eucalypti	CBS 141281	T	—	KX228303	—	—	—
Vermiculariopsiella eucalypticola	CBS 143442	T	—	MG386123	—	—	—
Vermiculariopsiella lauracearum	CBS 145055	T	—	MK047487	—	—	—
Vermiculariopsiella pini	CBS 146009	T	—	MN567635	—	—	—
Vialaea insculpta	DAOM 240257		JX139726	JX139726	—	—	—
Vialaea minutella	BRIP 56959		KC181926	KC181924	—	—	—
Woswasia atropurpurea	CBS 133167	T	—	JX233658	JX233658	—	JX233659
Xyladictyochaeta lusitanica	CBS 142290	T	KY853479	KY853543	—	—	—
Xylaria hypoxylon	CBS 122620, OSC 100004		KY610407	KY610495	AY544692	—	KY624231
Xylochrysis lucida	CBS 135996	T	—	KF539911	KF539912	—	KF539913
Xylomelasma sordida	CBS 116000	T	—	AY761087	AY761090	—	KY931929
Zanclospora jonesii	MFLUCC 15-1015	T	KY212753	KY212761	—	KY212757	
Zanclospora lateriphiala	S.M.H. 2629-1	T	—	AF466070	—	AF466031	
Zygosporium mycophilum	CBS 894.69		MH859474	MH871255	—	—	—
Zygosporium pseudomasonii	CBS 146059	T	MN562147	MN567654	—	—	MN556815

Notes: T, E, I, N and P denote ex-type, ex-epitype, ex-isotype, ex-neotype and ex-paratype strains.
