# Supplementary material for: Phylogeny, Global Biogeography and Pleomorphism of Zanclospora
Source: Microorganisms. 2021 Mar 29;9(4):706. doi: 10.3390/microorganisms9040706 (PMC8066784; doi:10.3390/microorganisms9040706)
Supplement: Supplementary file 1 [file microorganisms-09-00706-s001.zip › Supplementary Tables/Supplementary Table S5.pdf]

Table S5. The biogeography, substrate and habitat affinity of *Zanclospora* and outgroup taxa inferred from the GlobalFungi database.

Species	Abundance				Geographical origin (% of samples)							Substrate (% of samples)				Biomes (% of samples)							Other data[4] (% of samples)		
	samples	studies	reads	freq. r.[1]	NA	SA	E	AF	A	AU	AN	BS	DW	R	O[2]	F	W	S	G	D	A/T	O[3]	MAT	MAP	pH
Z. clavulata	1	1	1	18.9	0	0	0	0	0	100	0	100	0	0	0	100	0	0	0	0	0	0	11.7	1026	4.1
Z. jonesii	1	1	2	3.7	0	0	0	0	100	0	0	100	0	0	0	100	0	0	0	0	0	0	22.9	1842	n.a.
ITS1-ENV1	3	1	4	25.9	0	0	0	0	100	0	0	0	100	0	0	100	0	0	0	0	0	0	15.4	2145	4.5
ITS1-ENV2	1	1	1	28.7	0	0	0	0	100	0	0	0	100	0	0	100	0	0	0	0	0	0	16.6	1856	5.0
ITS1-ENV3	5	1	25	10.9	0	0	0	0	100	0	0	100	0	0	0	100	0	0	0	0	0	0	22.9	1842	n.a.
ITS1-ENV4	2	1	8	6.4	0	0	0	0	100	0	0	100	0	0	0	100	0	0	0	0	0	0	22.9	2110	3.9
ITS1-ENV5	2	1	10	13.9	0	0	0	0	100	0	0	100	0	0	0	50	0	0	0	0	50	0	26.8	2301	4.7
ITS1-ENV6	1	1	1	115.2	100	0	0	0	0	0	0	0	0	100	0	100	0	0	0	0	0	0	15.1	2736	n.a.
ITS2-ENV1	1	1	1	20.4	0	0	0	0	0	100	0	100	0	0	0	100	0	0	0	0	0	0	10.2	1793	3.3
ITS2-ENV2	1	1	1	10.4	0	0	0	0	0	100	0	100	0	0	0	100	0	0	0	0	0	0	10.5	2578	3.5
ITS2-ENV3	1	1	2	37.7	0	0	0	0	0	100	0	100	0	0	0	100	0	0	0	0	0	0	11.7	1026	4.1
ITS2-ENV4	1	1	2	8.7	0	0	0	0	0	100	0	100	0	0	0	0	0	100	0	0	0	0	19.8	1945	n.a.
ITS2-ENV5	1	1	2	8.7	0	0	0	0	0	100	0	100	0	0	0	0	0	100	0	0	0	0	19.8	1945	n.a.
ITS2-ENV6	1	1	89	1814.5	0	0	0	0	100	0	0	0	100	0	0	100	0	0	0	0	0	0	14.8	2407	4.4
ITS2-ENV7	1	1	1	20.4	0	0	0	0	100	0	0	0	100	0	0	100	0	0	0	0	0	0	14.8	2407	4.4
ITS2-ENV8	1	1	129	2630	0	0	0	0	100	0	0	0	100	0	0	100	0	0	0	0	0	0	14.8	2407	4.4
ITS2-ENV9	1	1	1	20.5	0	100	0	0	0	0	0	100	0	0	0	100	0	0	0	0	0	0	24.2	3203	2.6
ITS2-ENV10	3	2	14	19	0	100	0	0	0	0	0	33.3	0	66.7	0	100	0	0	0	0	0	0	24.3	2596	2.7
ITS2-ENV11	1	1	1	18	0	0	0	0	100	0	0	100	0	0	0	100	0	0	0	0	0	0	23.2	2671	3.5
**GlobalFungi**	20009	207	6.48×10^8		20.2	3.4	44.2	1.2	14.7	12.3	4	54.9	6.5	17.9	19.1	61.8	3.9	3.1	15.5	5.2	6.4	4.1	4.9	1000–2000	4.7
**Outgroups**																									
Chaetosphaeria minuta	1	1	1	8	0	0	0	0	0	100	0	100	0	0	0	100	0	0	0	0	0	0	22.8	1969	4.5
Chloridium virescens	342	16	33286	88.2	7.8	0	78.4	0	13.9	0	0	87.4	6.1	6.1	0.3	55.6	2.4	2.9	24.9	0.29	2.1	3	7.7	1160	3.8
Cryptophiale udagawae	3	2	3	8.3	0	0	0	0	2	1	0	1	2	0	0	3	0	0	0	0	0	0	18.5	2085	5.0
Dictyochaeta fuegiana	14	7	885	180.1	21.4	0	14.3	0	64.3	0	0	35.7	0	64.3	0	78.6	0	0.1	7.1	0	7.1	0	13	1805	4.6
Dictyochaeta callimorpha	6	2	4	2	0	0	33.3	0	0	66.7	0	100	0	0	0	66.7	0	0	0	0	33.3	0	10.6	1263.3	3.4
Dictyochaeta querna	19	5	350	9.7	5.3	0	63.2	0	31.6	0	0	68.4	0	31.6	0	89.5	0	0	5.3	0	5.3	0	14.8	1178	5.1
Kionochaeta castaneae	1	1	1	3.7	0	0	0	0	100	0	0	100	0	0	0	100	0	0	0	0	0	0	23	1087	4.0
Kionochaeta microspora	18	7	219	35.1	5.6	0	0	0	83.3	11.1	0	57.1	35.7	7.2	0	100	0	0	0	0	0	0	11.7	1337	4.9
Kionochaeta ramifera	25	4	584	75.4	36	48	0	0	12	4	0	64	4	32	0	88	0	0.1	0	0	0	0	20.8	2619	3.9

Notes:

NA = North America, SA = South America, E = Europe, AF = Africa, A = Asia, AU = Australia, AN = Antarctica;

BS = bulk soil, DW = dead wood, R = root, O = others;

F = forest, W = woodland, S = shrubland, G = grassland, D = desert, A/T = anthropological/terrestrial, O = others;

1 Frequency of reads (% × 10^3) of the particular taxon across samples where the taxon was found.

2 Other substrates in the GlobalFungi database represent litter (3.43 % of all deposited samples), lichen (0.33 %), rhizosphere soil (1.5 %) and shoot (13.23 %).

3 Other biomes in the GlobalFungi databases represent freshwater (0.03 % of all samples), mangrove (0.01 %) marine (0.85 %) and polar desert (0.23 %), tundra (3 %).

4 Average values across all samples are presented. MAT: Mean Annual Temperature (°C), MAP: Mean Annual Precipitation (mm), pH (AVG).
