# Supplementary material for: Phylogeny, Global Biogeography and Pleomorphism of Zanclospora
Source: Microorganisms. 2021 Mar 29;9(4):706. doi: 10.3390/microorganisms9040706 (PMC8066784; doi:10.3390/microorganisms9040706)
Supplement: Supplementary file 1 [file microorganisms-09-00706-s001.zip › Supplementary Tables/Supplementary Table S6.pdf]

Table S6. Published records of *Zanclospora* with host, substrate, country of the collection and references.

Taxon	Country	Host	Substrate	Reference
Zanclospora aurea	New Zealand	unidentified	decaying wood	This study
Zanclospora austroamericana	Brazil	*Eucalyptus propinqua*	dead bark	[3]
Zanclospora bicolorata	Ecuador	unidentified	decaying leaves	[10]
Zanclospora bonfinensis	Brazil	unidentified	decaying leaves	[8]
Zanclospora brevispora var. brevispora	Brazil	unidentified	decaying leaves	[27]
	Brunei	*Pandanus* sp.	decaying leaves	[28]
	Cuba	unidentified	deadwood	[25]
	China	unidentified	submerged wood	[26]
	Kenya	unidentified	decaying wood	[24]
	New Zealand	*Nothofagus solandri* var. *cliffortioides*	decaying wood and bark	[1, 11]
	Seychelles	*Pandanus seychellarum*	decaying leaves	[28]
Zanclospora brevispora var. transvaalensis	South Africa	unidentified	decaying wood	[5]
Zanclospora clavulata	Portugal	unidentified	decaying wood	This study
Zanclospora falcata	New Zealand	unidentified	decaying wood	This study
Zanclospora iberica	Spain	unidentified	decaying wood, plant debris	[9], this study
Zanclospora indica	Brazil	unidentified	decaying leaves	[93]
	Cuba	*Nectandra coriacea*	decaying leaves	[25, 91]
	India	*Gymnosporangia emarginata*	decaying leaves	[2]
	Ivory Coast	unidentified	decaying leaves	[92]
Zanclospora jonesii	Thailand	unidentified	decaying wood	[86]
Zanclospora lateriphiala	USA	unidentified	decaying wood	[12, 21], this study
Zanclospora mystica	Ivory Coast	unidentified	dead leaves	[4]
Zanclospora novae-zelandiae	New Zealand	*Libocedrus bidwii* , *Nothofagus fusca* , *N. solandri* var. *cliffortioides, N. truncata, Nothofagus* sp.	decaying wood and bark	[1], this study
Zanclospora cf. novae-zelandiae	Brazil	unidentified	decaying wood and bark	[93]
	Cuba	unidentified	decaying wood	[25]
	Japan	*Fagus crenatus*	decaying wood and bark	[20]
	Taiwan	unidentified	decaying wood	[22]
	Vietnam	unidentified	decaying bark	[23]
Zanclospora phaeostalacta	New Zealand	unidentified	decaying wood	[85]
Zanclospora ramifera	New Zealand	*Nothofagus* sp., unidentified broadleaf tree	decaying wood	This study
Zanclospora sylvatica	Puerto Rico	unidentified	decaying wood	[12]
Zanclospora tropicalis	Puerto Rico	unidentified	decaying wood	[12]
	Costa Rica	unidentified	decaying wood	[12]
Zanclospora xylophila	New Zealand	unidentified	decaying wood	This study
